# AARS1-mediated lactylation of H3K18 and STAT1 promotes ferroptosis in diabetic nephropathy

**DOI:** 10.1038/s41418-025-01587-4

**Published:** 2025-09-23

**Authors:** Jia Hong, Hongjiao Xu, Lang Yu, Zhuang Yu, Xiangyuan Chen, Zhipeng Meng, Jiali Zhu, Jinbao Li, Minmin Zhu

**Affiliations:** 1 Department of Anesthesiology, Shanghai General Hospital, Shanghai Jiao Tong University School of Medicine, Shanghai, China; 2 Department of Anesthesiology, Shanghai General Hospital of Nangjing Medical University, Shanghai, China; 3 Department of Anesthesiology, Huzhou Central Hospital, Affiliated Central Hospital of HuZhou University, No.1558 Sanhuan North Road, Huzhou, Zhejiang China; 4 Department of Anesthesiology, Shanghai Pudong Hospital; Fudan University Pudong Medical Center, Shanghai, China

**Keywords:** Endocrine system and metabolic diseases, Metabolic disorders

## Abstract

Diabetic nephropathy (DN) is the primary cause of end-stage renal disease worldwide. Recent studies have revealed that lactate-mediated histone lactylation, which functions as a novel epigenetic modification, is involved in the occurrence and development of diabetes-related complications. However, little is known about the role of lactyltransferase in DN. Alanyl-tRNA synthetase 1 (AARS1) was identified as a novel lactyltransferase that modulates histone H3-lysine-18 lactylation (H3K18la). In the present study, we determined whether AARS1-mediated H3K18la participates in the pathogenesis of DN. More importantly, we explored the potential mechanism involved. A mouse DN model consisting of both wild-type and alanyl-tRNA synthetase (AARS1) heterozygote (AARS1^+/–^) mice was utilized in this study. Transcriptomic and lipidomic analyses, combined with a variety of molecular biological methodologies, were employed to elucidate the potential mechanism by which AARS1 regulates ferroptosis in DN. Our results indicated that the increases in AARS1 and H3K18la expression were involved in kidney dysfunction and renal cell death via the modulation of ferroptosis in the DN model. Moreover, AARS1 induced lipid peroxidation by increasing fatty acid elongase-5 (ELOVL5) transcription, ultimately contributing to ferroptosis induction. Furthermore, AARS1 interacted with signal transducer and activator of transcription 1 (STAT1) to jointly regulate ELOVL5 transcription. Additionally, treatment with the STAT1-specific inhibitor fludarabine delayed DN progression. In addition, we observed that AARS1 modulated the lactylation of both STAT1 and H3K18 to regulate ELOVL5 transcription, thus triggering ferroptosis. Inhibition of AARS1-induced lactylation via β-alanine attenuated ferroptosis in DN model mice and hyperglycaemic cells. The present study showed that AARS1 induced the lactylation of H3K18 and STAT1 to regulate ELOVL5 transcription, thus triggering ferroptosis in a diabetic nephropathy model.

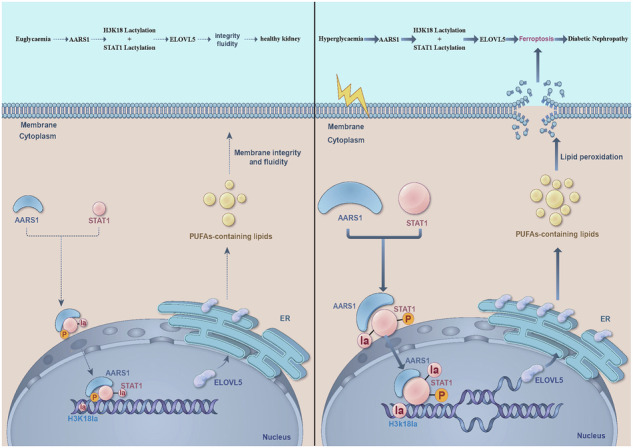

## Introduction

Diabetic nephropathy (DN) is one of the most detrimental complications of diabetes mellitus [1], and 20–40% of individuals who require renal replacement therapy are diagnosed with DN, leading to more than 950,000 deaths worldwide annually [2]. When DN progresses to renal failure, patient treatment costs and mortality both increase sharply, and few effective treatment approaches for DN are available [3, 4]. Hence, exploring new molecular pathways related to DN and identifying potential therapeutic targets to prevent or mitigate renal dysfunction are important.

The processes that reportedly participate in DN include the endothelial‒mesenchymal transition [5], mitochondrial damage [6], endothelial inflammation [7], and apoptosis [8], among others [9]. In addition to the above attributes, ferroptosis is also reported to be an important characteristic of DN that leads to renal cell death [10]. Moreover, glomerular endothelial cells and proximal tubular epithelial cells are vulnerable to ferroptosis [11, 12]. As a novel type of iron-dependent programmed cell death [13], ferroptosis primarily occurs when polyunsaturated fatty acids (PUFAs) on the cell membrane are catalysed by divalent iron or esteroxylase, thus resulting in liposomal peroxidation and ultimately cell death [14]. Consistently, the PUFA biosynthesis pathway was found to play an important role in ferroptosis [15]. Moreover, the susceptibility of phospholipid-polyunsaturated fatty acids to peroxidation and their close association with ferroptosis make them key factors in DN [15, 16]. ELOVL fatty acid elongase-5 (ELOVL5) is one of seven ELOVL family members expressed in mammals [17]. Previous studies have indicated that ELOVL5 is involved in ferroptosis [15]. However, the exact mechanism by which ELOVL5 modulates ferroptosis in DN has not yet been studied.

Epigenetics plays a crucial role in diabetes-related complications [18]. Our previous studies demonstrated that both histone methylation and histone acetylation are involved in the occurrence and development of DN [19, 20]. Recent studies have revealed that lactate-mediated histone lactylation, which functions as a novel epigenetic modification, is also involved in the occurrence and development of diabetes-related complications [21, 22]. Moreover, histone H3-lysine 18 lactylation (H3K18la) is considered a tissue-specific active enhancer [23]. These studies [21–23] strongly suggest that lactate-mediated H3K18la may be involved in the occurrence and progression of diabetes-related complications. However, little is known about the role of lactyltransferases or delactyltransferases in DN. Exploring potential lactyltransferases and delactyltransferases is crucial and urgent, and new therapeutic strategies for DN are needed. Recently, alanyl-tRNA synthetase 1 (AARS1) was identified as a novel lactyltransferase that modulates H3K18la and nonhistone lactylation [24, 25]. In the present study, we determined whether AARS1-mediated H3K18la and nonhistone lactylation participated in the pathogenesis of DN. More importantly, we also explored the potential mechanism involved.

## Results

### The expression of AARS1 and H3K18la was increased in DN patients and models

**Fig. 1:**
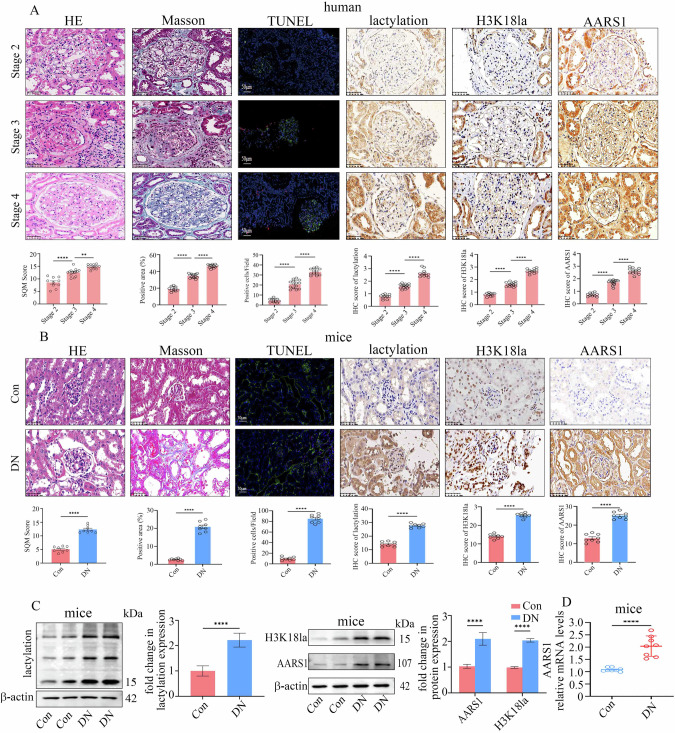
AARS1 and H3K18la expression is increased in diabetic nephropathy (DN) patients and mice. **A** Representative images of HE staining, Masson’s trichrome staining, TUNEL staining, and IHC staining for AARS1, lactylation, and H3K18la in renal biopsy samples from the participants in the present study (scale bar: 50 μm). The degree of damage to the kidney tissue structure, fibrosis, and kidney cell death, as well as the levels of AARS1, lactylation, and H3K18la, were increased in the kidneys of DN patients as the DN stage progressed. **B** Representative images of HE staining, Masson’s trichrome staining, TUNEL staining, and IHC staining for AARS1, lactylation, and H3K18la in renal biopsy samples from the control (Con) and DN model mice used in the present study (scale bar: 50 μm). The degree of damage to the kidney tissue structure, fibrosis, and kidney cell death, as well as the levels of AARS1, lactylation, and H3K18la, were increased in the kidneys of DN model mice. **C** Western blotting assays revealed that the protein levels of AARS1, lactylation, and H3K18la were increased in the kidneys of DN model mice. **D** qPCR assays indicated that the mRNA levels of AARS1 were increased in the kidneys of DN model mice. (*P < 0.05, **P < 0.01, ***P < 0.001, and ****P < 0.0001).

The characteristics of the DN patients included in this study are shown in Supplementary Table 1. The DN patients were grouped into stage 2, stage 3 or stage 4 according to the estimated glomerular filtration rate (eGFR). Haematoxylin–eosin (HE) staining of the samples from DN patients is shown in Fig. 1A. Masson’s trichrome staining revealed greater collagen deposition and interstitial fibrosis as the DN stage progressed (Fig. 1A). Consistently, the TUNEL assay results indicated that cell death increased in the kidneys of DN patients as the DN stage progressed (Fig. 1A). In addition, cell death was observed in both renal tubules and glomeruli (Fig. 1A). Previous studies [21–23] have suggested that H3K18la may be involved in the occurrence and progression of diabetes-related complications. Moreover, AARS1 was verified as a novel lactyltransferase that modulates H3K18la [24, 25]. Therefore, we detected the levels of AARS1, lactylation and H3K18la in the kidneys of DN patients. Our data indicated that the levels of AARS1, lactylation, and H3K18la were increased in the kidneys of DN patients as the DN stage progressed (Fig. 1A).

The serum biochemical indices of the mice used in the present study are shown in Supplementary Table 2. HE and Masson’s trichrome staining revealed significant changes in the kidneys of DN mice (Fig. 1B). TUNEL staining suggested an increase in cell death in the kidneys of DN mice (Fig. 1B). Moreover, the protein levels of AARS1, lactylation, and H3K18la were increased in the kidneys of DN mice (Fig. 1B, C). Consistently, the mRNA levels of AARS1 were increased in the kidneys of DN mice (Fig. 1D).

In addition, high-glucose treatment increased AARS1, lactylation, and H3K18la levels in human glomerular endothelial cells (HGECs) and human renal tubular epithelial (HK-2) cells (Supplementary Fig. 1A, B). Moreover, cell death was enhanced in hyperglycaemic cells (Supplementary Fig. 1C). Furthermore, mannitol treatment, which was used as an osmotic control, had no effect on AARS1, lactylation, or H3K18la levels or on cell death in hyperglycaemic cells (Supplementary Fig. 1A–C). These data indicated that the levels of AARS1, lactylation, and H3K18la were increased in DN patients and models.

### AARS1 upregulation participated in the development of DN

**Fig. 2:**
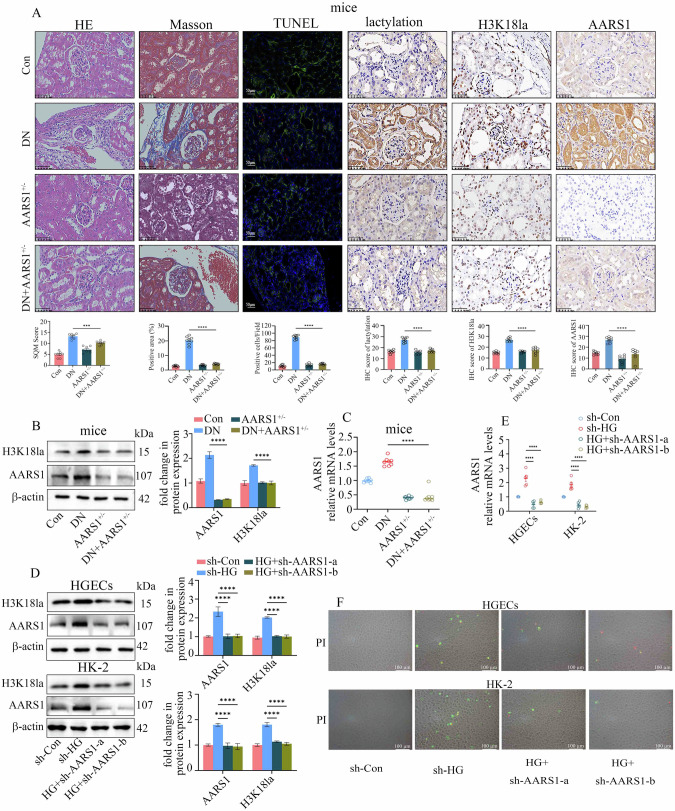
AARS1 upregulation participated in the development of diabetic nephropathy (DN). **A** Representative images of HE staining, Masson’s trichrome staining, TUNEL staining, and IHC staining for AARS1, lactylation, and H3K18la in renal biopsy samples from the control (Con), DN, AARS1^+/–^, and AARS1^+/–^ DN model mice used in the present study (scale bar: 50 μm). Compared with DN model mice, AARS1^+/–^ DN model mice presented less damage to the kidney tissue structure, alleviated fibrosis, decreased kidney cell death, and reduced AARS1, lactylation, and H3K18la levels. **B** Compared with those in DN model mice, the protein levels of AARS1 and H3K18la were decreased in the kidneys of AARS1^+/–^ DN model mice. **C** Compared with those in DN model mice, the mRNA levels of AARS1 were decreased in the kidneys of AARS1^+/–^ DN model mice. **D** High-glucose treatment increased the levels of AARS1 and H3K18la, changes that were reversed by AARS1 silencing in HGECs and HK-2 cells. **E** High-glucose treatment increased the AARS1 mRNA level in HGECs and HK-2 cells, which was reversed by AARS1 silencing. **F** A PI assay indicated that high-glucose treatment increased cell death, which was reversed by AARS1 silencing in HGECs and HK-2 cells (scale bar: 100 μm). (*P < 0.05, **P < 0.01, ***P < 0.001, and ****P < 0.0001).

We used AARS1 heterozygous (AARS1^+/–^) mice to establish a DN model (AARS1^+/-^DN mice) and further explore the function of AARS1 in DN (Fig. 2A–C). Compared with DN mice, AARS1^+/-^ DN mice presented less damage to the kidney tissue structure; alleviated fibrosis; decreased kidney cell death; reduced AARS1, lactylation, and H3K18la levels (Fig. 2A‒C); and improved kidney function (Supplementary Table 2). Consistent with these findings, silencing AARS1 decreased the expression of AARS1 and H3K18la in hyperglycaemic cells (Fig. 2D, E). Moreover, silencing AARS1 reversed the high-glucose-mediated death of HGECs and HK-2 cells (Fig. 2F). Furthermore, Gln-AMS, an AARS1 inhibitor, inhibited AARS1 and H3K18la expression in a concentration- and time-dependent manner (Supplementary Fig. 1D, E). Furthermore, Gln-AMS treatment decreased the death of hyperglycaemic cells (Supplementary Fig. 1F). These data indicate the involvement of AARS1-induced H3K18la in the development of DN.

### AARS1 upregulation is involved in the pathogenesis of DN by triggering ferroptosis

**Fig. 3:**
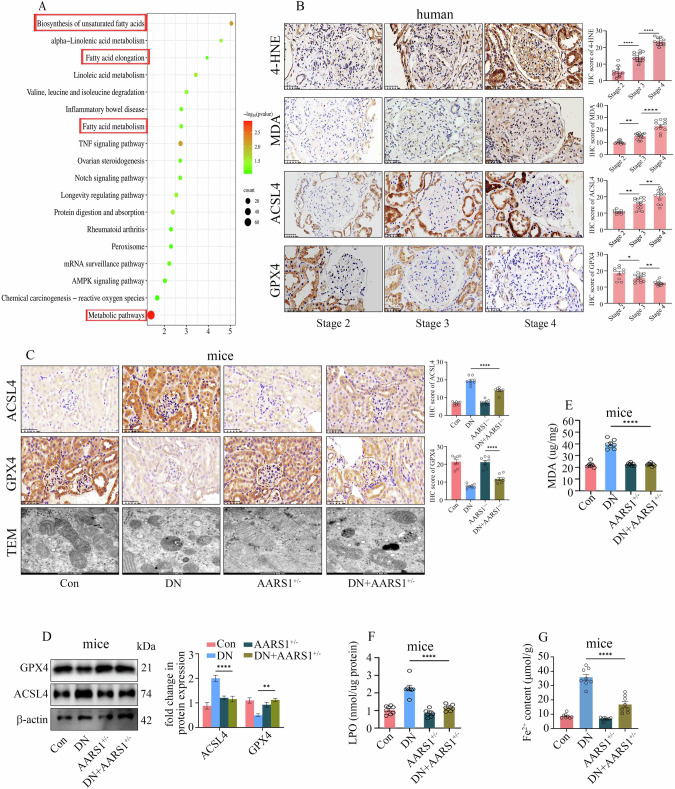
AARS1 upregulation is involved in the pathogenesis of diabetic nephropathy (DN) by triggering ferroptosis. **A** Molecular functions of the differentially expressed genes according to the Kyoto Encyclopedia of Genes and Genomes (KEGG) analysis. **B** Representative images of IHC staining for 4-HNE, MDA, ACSL4, and GPX4 in renal biopsy samples from the participants in the present study (scale bar: 50  μm). IHC revealed that the levels of 4-HNE, MDA, and ACSL4 gradually increased, whereas the level of GPX4 gradually decreased in the kidneys of DN patients as the DN stage progressed. **C** Representative images of IHC staining for ACSL4 and GPX4 and transmission electron microscopy (TEM) images of renal biopsy samples from the control (Con), DN, AARS1^+/–^, and AARS1^+/–^ DN model mice used in the present study (scale bar: 50 μm for IHC; scale bar: 500 nm for TEM). Compared with DN model mice, AARS1^+/–^ DN model mice presented reduced ACSL4 expression, upregulated GPX4 level,s and increased mitochondrial ridges and mitochondrial volumes. **D** Western blotting assays revealed that compared with DN model mice, AARS1^+/–^ DN model mice presented decreased ACSL4 levels and increased GPX4 expression in the kidney. **E** Compared with those in DN model mice, the MDA levels in the kidneys of AARS1^+/-^ DN model mice were decreased. **F** Compared with those in DN model mice, LPO levels were decreased in the kidneys of AARS1^+/–^ DN model mice. **G** Compared with that in DN mice, the Fe^2+^ content was decreased in the kidneys of AARS1^+/–^ DN model mice. (*P < 0.05, **P < 0.01, ***P < 0.001, and ****P < 0.0001).

An RNA-seq analysis of mouse kidneys was performed to further explore the mechanism by which AARS1-induced H3K18la participates in the pathogenesis of DN. Our data revealed that in the kidneys of DN mice, KEGG pathways related to the biosynthesis of unsaturated fatty acids, fatty acid elongation, fatty acid metabolism, and metabolic pathways were enriched (Fig. 3A, Supplementary Table 3). Previous studies have indicated that the PUFA biosynthesis pathway plays a crucial role in ferroptosis [15]. Similarly, the levels of 4-hydroxy-2-nonenal (4-HNE), malondialdehyde (MDA), and acyl-CoA synthetase long-chain family member 4 (ACSL4) gradually increased, whereas the level of glutathione peroxidase 4 (GPX4) gradually decreased in the kidneys of DN patients as the DN stage progressed (Fig. 3B), indicating that ferroptosis participated in the development of DN. We further verified whether ferroptosis was involved in the progression of DN by treating DN mice and high-glucose-exposed cells with the ferroptosis inhibitor ferrostatin-1 (Fer-1). Fer-1 alleviated kidney tissue damage, inhibited ACSL4 expression, and increased GPX4 levels (Supplementary Fig. 2A, B). Moreover, according to transmission electron microscopy (TEM), the features of ferroptosis, such as a dense mitochondrial structure with significantly reduced mitochondrial ridges and a smaller volume of mitochondria, were observed in DN mice, which were attenuated upon Fer-1 treatment (Supplementary Fig. 2A). Furthermore, Fer-1 also reduced kidney MDA and lipid peroxide (LPO) deposition, decreased the Fe^2+^ content, and ameliorated renal dysfunction in DN mice (Supplementary Fig. 2C–E; Supplementary Table 2). In hyperglycaemic cells, Fer-1 decreased cell death in hyperglycaemic cells in a time- and concentration-dependent manner (Supplementary Fig. 3A). Moreover, Fer-1 reduced ACSL4 expression and enhanced GPX4 levels in hyperglycaemic cells (Supplementary Fig. 3B). C11-BODIPY 581/591 (a lipid peroxidation probe) fluorescence staining revealed that Fer-1 inhibited high-glucose-mediated lipid peroxidation (Supplementary Fig. 3C). JC-1 is a mitochondrial membrane potential (MMP) probe, and fluorescence staining indicated that Fer-1 treatment ameliorated the high glucose-mediated destruction of the MMP in HGECs and HK-2 cells (Supplementary Fig. 3D). Fer-1 treatment decreased high-glucose-induced MDA levels (Supplementary Fig. 3E). Fluorescence staining for FeRhoNox-1, a specific Fe^2+^ probe, showed that Fer-1 treatment decreased the Fe^2+^ content in hyperglycaemic cells (Supplementary Fig. 3F). The fluorescence staining for MitoSOX Red, a mitochondria-specific superoxide indicator, indicated that Fer-1 treatment reduced mitochondrial superoxide accumulation (Supplementary Fig. 3G).

We detected ferroptosis-related indicators in DN models to further confirm that AARS1-induced H3K18la regulates ferroptosis in DN. Our data indicated that ACSL4 expression was decreased, GPX4 levels were increased, ferroptosis was improved in TEM images, the MDA and LPO contents were decreased, and the Fe^2+^ content was reduced in the kidneys of the AARS1^+/–^ DN mice compared with the kidneys of the DN mice (Fig. 3C–G). Similarly, the levels of ferroptosis markers were increased in hyperglycaemic HGECs and HK-2 cells, which were reversed by AARS1 silencing (Supplementary Fig. 4). Consistently, Gln-AMS treatment inhibited ferroptosis in hyperglycaemic cells (Supplementary Fig. 5). These data indicate that AARS1-induced H3K18la expression participates in DN by triggering ferroptosis.

### AARS1-induced H3K18la participates in ferroptosis in DN models via the modulation of ELOVL5 transcription

Next, we explored the exact mechanism by which AARS1-induced H3K18la participates in ferroptosis in individuals with DN. The RNA-seq analysis revealed that ELOVL5, trans-2,3-enoyl-CoA reductase (TECR), and 3-hydroxyacyl-CoA dehydratase 1 (HACD1) are involved in multiple pathways, including the biosynthesis of unsaturated fatty acids, fatty acid metabolism, fatty acid elongation and metabolism pathways (Supplementary Fig. 6A, Supplementary Table 3). Among the three genes, only ELOVL5 was present in the H3K18la ChIP-seq results (Supplementary Table 4). ChIP assays confirmed that AARS1 and H3K18la were enriched only in the promoter region of ELOVL5 via primer 3 (P3), and re-ChIP assays indicated that AARS1 and H3K18la occupied the same region of the ELOVL5 promoter (Supplementary Fig. 6B). ELOVL5 expression levels gradually increased in the kidneys of DN patients as the DN stage progressed (Supplementary Fig. 6C). Consistently, the expression of ELOVL5 was upregulated in DN model mice (Supplementary Fig. 6D–F) and hyperglycaemic cells (Supplementary Fig. 6G, H). The inhibition of ELOVL5 expression attenuated ferroptosis in hyperglycaemic HGECs and HK-2 cells (Supplementary Fig. 7). An analysis of lipid metabolism in ELOVL5-silenced cells treated with high glucose revealed a decrease in the production of PUFAs, including arachidonic acid (AA) (Supplementary Fig. 8A, Supplementary Table 5). Moreover, IF data indicated that ELOVL5 was located mainly in the endoplasmic reticulum (Supplementary Fig. 8B). Furthermore, incubating ELOVL5-silenced cells with AA reversed the protective effect of si-ELOVL5 on high-glucose-mediated ferroptosis (Supplementary Fig. 8C–H). In addition, ELOVL5 silencing attenuated ferroptosis mediated by FINO_2_ (a ferroptosis inducer) (Supplementary Fig. 9). These data indicate that ELOVL5 triggers ferroptosis by regulating PUFA synthesis in the endoplasmic reticulum in individuals with DN.

**Fig. 4:**
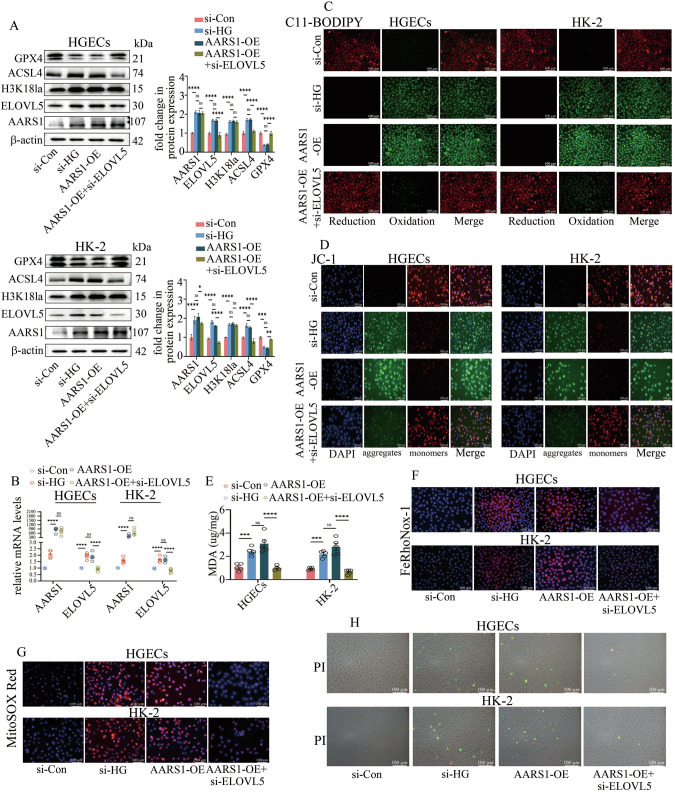
AARS1 modulates ELOVL5 transcription to mediate ferroptosis in hyperglycaemic cells. **A** Western blotting assays indicated that AARS1 overexpression increased H3K18la, ELOVL5, and ACSL4 levels and decreased GPX4 levels in cells. Moreover, ELOVL5 silencing reversed the AARS1 overexpression-mediated increase in ACSL4 expression and decrease in GPX4 expression. **B** Results of the qPCR analysis of AARS1 and ELOVL5 levels in HGECs and HK-2 cells. **C** A C11-BODIPY 581/591 fluorescence probe was used to detect lipid peroxidation levels in HGECs and HK-2 cells. The results indicated that AARS1 overexpression increased lipid peroxidation levels, which was reversed by ELOVL5 silencing (scale bar: 100 μm). **D** A JC-1 fluorescence probe was used to detect changes in the mitochondrial membrane potential (MMP) of HGECs and HK-2 cells. Our results showed that the probes in the AARS1-overexpressing group were mainly green fluorescent monomers. In contrast, after the intervention with si-ELOVL5, the probes were converted into red fluorescent polymers, indicating that ELOVL5 silencing attenuated the destruction of the MMP in AARS1-overexpressing cells (scale bar: 100 μm). **E** MDA levels were elevated in AARS1-overexpressing cells, which were reversed via ELOVL5 silencing. **F** A FeRhoNox-1 fluorescent probe was used to detect the Fe^2+^ content in the cells. Our results revealed that FeRhoNox-1 fluorescence was increased in AARS1-overexpressing cells but was decreased after ELOVL5 silencing (scale bar: 100 μm). **G** The red fluorescence intensity of MitoSOX, a mitochondria-specific superoxide indicator, was significantly increased in AARS1-overexpressing cells, but was reversed by ELOVL5 silencing (scale bar: 100 μm). **H** A PI assay indicated that AARS1 overexpression increased cell death, and this change was reversed by ELOVL5 silencing in HGECs and HK-2 cells (scale bar: 100 μm). (*P < 0.05, **P < 0.01, ***P < 0.001, and ****P < 0.0001).

Next, we determined whether AARS1 mediated ferroptosis in individuals with DN by modulating ELOVL5 transcription. Our data showed that AARS1 overexpression increased ELOVL5 expression (Fig. 4A, B) and promoted ferroptosis (Fig. 4) in cells. Moreover, the effect of AARS1 overexpression on ferroptosis was abrogated by ELOVL5 silencing (Fig. 4). Furthermore, the inhibition of AARS1 expression attenuated ELOVL5 levels in the kidneys of DN model mice (Supplementary Fig. 10A–C) and in hyperglycaemic cells (Supplementary Fig. 10D–G). These data indicated that AARS1 promoted ferroptosis by increasing ELOVL5 transcription in the DN models.

### AARS1 directly interacted with signal transducer and activator of transcription 1 (STAT1) both in vivo and in vitro

**Fig. 5:**
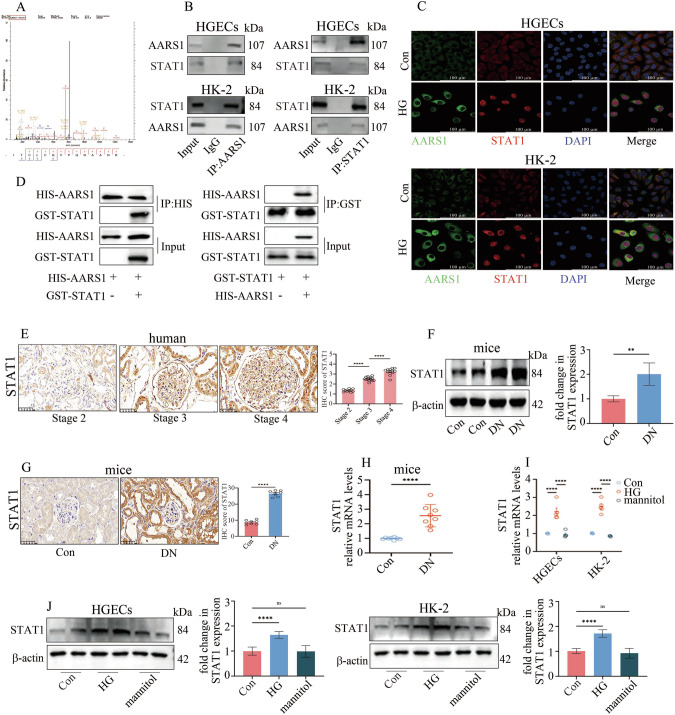
AARS1 directly interacts with STAT1. **A** A mass spectrometry assay indicated that STAT1 may bind to AARS1. **B** The interaction between STAT1 and AARS1 in HGECs and HK-2 cells was verified by Co-IP. **C** STAT1 and AARS1 were colocalized in HGECs and HK-2 cells, as detected via an immunofluorescence assay (scale bar = 100 μm). **D** The direct interaction between STAT1 and AARS1 was verified by GST pull-down assays. **E** IHC data indicating that STAT1 levels in renal biopsy samples from diabetic nephropathy (DN) patients gradually increased as the DN stage progressed (scale bar: 50 μm). **F** Western blotting assays revealed that the STAT1 protein level was increased in the kidneys of DN model mice. **G** IHC data indicating that STAT1 levels are increased in the kidneys of DN model mice (scale bar: 50 μm). **H** qPCR revealed that the STAT1 mRNA level was increased in the kidneys of DN model mice. **I** qPCR assay results indicating that the STAT1 mRNA level was increased in hyperglycaemic cells. **J** Western blotting assays indicated that the STAT1 protein level was increased in hyperglycaemic cells. (*P < 0.05, **P < 0.01, ***P < 0.001, and ****P < 0.0001).

Histone modifications in gene promoter regions often coincide with transcription factor binding, which leads to the transcription of downstream genes. A mass spectrometry analysis was performed in the present study to identify the transcription factors that interact with AARS1. Our data indicated that STAT1 may interact with AARS1 (Fig. 5A, Supplementary Table 6). Co-IP and IF results revealed that STAT1 bound to AARS1 in HGECs and HK-2 cells (Fig. 5B, C), and the direct binding between AARS1 and STAT1 was further confirmed by a GST pull-down assay (Fig. 5D). Furthermore, STAT1 expression was evaluated in renal biopsy samples from DN patients and DN models. Our data indicated that STAT1 levels gradually increased as the DN stage progressed (Fig. 5E). Consistently, STAT1 expression was upregulated in the kidneys of DN model mice (Fig. 5F–H) and in hyperglycaemic cells (Fig. 5I, J).

### STAT1 participates in ferroptosis by regulating ELOVL5 transcription in DN models

We performed both loss-of-function and gain-of-function experiments to determine the effects of STAT1 on ELOVL5 expression and ferroptosis. STAT1 silencing attenuated ELOVL5 levels and inhibited ferroptosis in hyperglycaemic HGECs and HK-2 cells (Supplementary Fig. 11). Moreover, STAT1 overexpression upregulated ELOVL5 expression and activated ferroptosis in HGECs and HK-2 cells, changes that were abrogated by ELOVL5 silencing (Supplementary Fig. 12). ChIP and luciferase assays were performed in this study to determine whether STAT1 directly modulates ELOVL5 transcription. ChIP assays revealed that STAT1 was enriched in the ELOVL5 promoter, and re-ChIP assays revealed that STAT1 and AARS1 occupied the same region of the ELOVL5 promoter (Supplementary Fig. 13A). Next, JASPAR (https://jaspar.genereg.net/) was used to predict the potential binding site for STAT1 in the ELOVL5 promoter region, which is shown in Supplementary Table 7. The motif logo and position weight matrix are shown in the upper and lower panels, respectively (Supplementary Fig. 13B). Then, we constructed a plasmid in which the two binding sites were mutated and found that ELOVL5 transcription was significantly inhibited upon the mutation of these sites (Supplementary Fig. 13C). Therefore, we suggest that STAT1 directly binds to the ELOVL5 promoter region and participates in the modulation of ELOVL5 transcription.

**Fig. 6:**
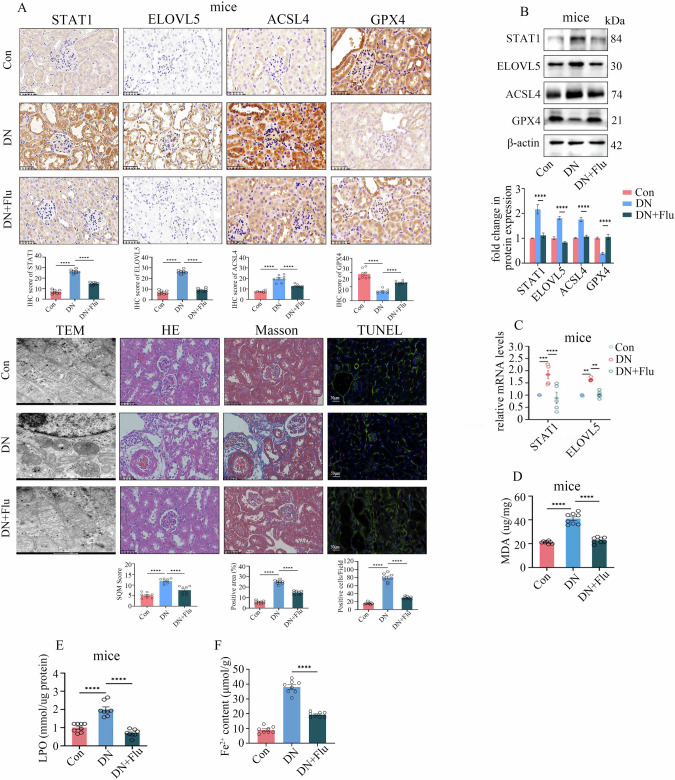
The STAT1 inhibitor fludarabine (Flu) inhibits ferroptosis and improves renal dysfunction in diabetic nephropathy (DN) model mice. **A** Representative images of HE staining, Masson’s trichrome staining, TUNEL staining, IHC staining for STAT1, ELOVL5, ACSL4, and GPX4, and transmission electron microscopy (TEM) of renal biopsy samples from the control (Con), DN, and DN+Flu mice (scale bar: 50 μm for HE, Masson, TUNEL, and IHC; scale bar: 500 nm for TEM). Compared with DN mice, DN+Flu mice presented less kidney tissue damage, fibrosis, and cell death, reduced STAT1, ELOVL5, and ACSL4 expression, upregulated GPX4 levels, and increased mitochondrial ridges and volumes in kidney tissues. **B** Western blotting assays indicated that, compared with DN mice, DN+Flu mice presented decreased STAT1, ELOVL5, and ACSL4 levels and increased GPX4 expression in kidney tissues. **C** qPCR assays indicated that compared with DN mice, DN+Flu mice presented decreased STAT1 and ELOVL5 mRNA expression in kidney tissue. **D** Compared with those in DN mice, the MDA levels were decreased in the kidneys of DN+Flu mice. **E** Compared with those in DN mice, LPO levels were decreased in the kidneys of DN+Flu mice. **F** Compared with that in DN mice, the Fe^2+^ content was decreased in the kidneys of DN+Flu mice. (*P < 0.05, **P < 0.01, ***P < 0.001, and ****P < 0.0001).

We further determined whether STAT1 is a potential target for inhibiting ferroptosis in DN. For the cellular experiments, we used fludarabine (Flu), a STAT1-specific inhibitor. The findings revealed that fludarabine inhibited STAT1 expression in a concentration and time-dependent manner (Supplementary Fig. 14A). Moreover, Flu not only inhibited STAT1 expression but also decreased ELOVL5 levels (Supplementary Fig. 14B, C), thus leading to the suppression of ferroptosis downstream (Supplementary Fig. 14B–I). Consistently, the intraperitoneal injection of fludarabine in DN model mice effectively downregulated STAT1 and ELOVL5 expression (Fig. 6A–C), impeded ferroptosis (Fig. 6A–F), reduced kidney injury (Fig. 6A), and substantially improved kidney dysfunction (Supplementary Table 2). These data indicate that a STAT1 inhibitor may hold considerable clinical value in protecting against DN.

### AARS1 modulates the lactylation of both STAT1 and H3K18 to regulate ELOVL5 transcription and trigger ferroptosis

**Fig. 7:**
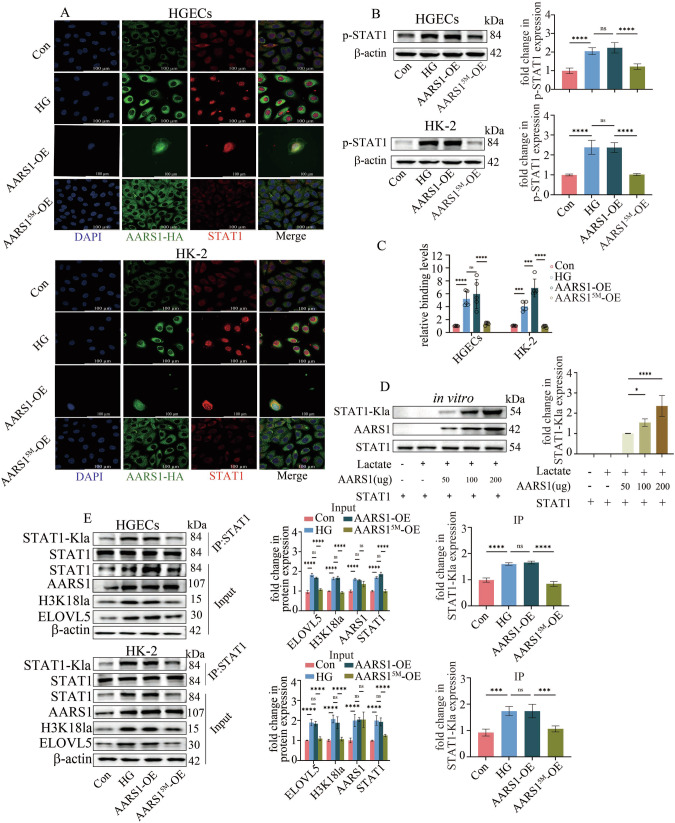
AARS1 modulates the lactylation of both STAT1 and H3K18 to regulate ELOVL5 transcription and trigger ferroptosis. **A** Immunofluorescence assay results indicated that AARS1 overexpression promoted STAT1 nuclear translocation in HGECs and HK-2 cells. However, AARS1^5M^ did not have these effects (scale bar: 100 μm). **B** Western blotting assays indicated that AARS1 overexpression increased STAT1 phosphorylation in HGECs and HK-2 cells. However, AARS1^5M^ did not have these effects. **C** ChIP‒qPCR revealed that AARS1 overexpression enhanced the binding of STAT1 to the ELOVL5 promoter region in HGECs and HK-2 cells. However, AARS1^5M^ did not have these effects. **D** AARS1 lactylated STAT1 with lactate in vitro. **E** Co-IP assays indicated that AARS1 induced STAT1 lactylation in HGECs and HK-2 cells. Moreover, AARS1 overexpression increased STAT1, H3K18la and ELOVL5 levels in HGECs and HK-2 cells. However, AARS1^5M^ did not have these effects. (*P < 0.05, **P < 0.01, ***P < 0.001, and ****P < 0.0001).

Next, we determined whether AARS1 affects STAT1 transcriptional activity. Our data indicated that the overexpression of AARS1 promoted STAT1 nuclear translocation (Fig. 7A), increased STAT1 phosphorylation (Fig. 7B), and enhanced the binding between STAT1 and the ELOVL5 promoter region (Fig. 7C). However, AARS1^5M^, which lacks lactyltransferase activity [24], did not have these effects (Fig. 7A–C). These data indicate that AARS1 may increase STAT1 transcriptional activity via the regulation of STAT1 lactylation. Both in vitro and in vivo lactylation assays were employed in this study to verify our hypothesis. Our in vitro lactylation study indicated that AARS1 utilized lactate to induce STAT1 lactylation in a concentration-dependent manner (Fig. 7D). AARS1 was also confirmed to induce STAT1 lactylation in HGECs and HK-2 cells (Fig. 7E). Moreover, AARS1 overexpression increased STAT1, H3K18la and ELOVL5 expression (Fig. 7E) and promoted ferroptosis in HGECs and HK-2 cells (Supplementary Fig. 15). However, AARS1^5M^ did not have these effects (Fig. 7E; Supplementary Fig. 15). We further elucidated the critical role of AARS1-mediated STAT1 lactylation in STAT1 transcriptional function by mutating the STAT1 lactylation sites, as reported in a previous study [26]. Our data revealed that the STAT1^K193^ site was the most important lactylation site regulated by AARS1 (Supplementary Fig. 16A). Additionally, mutation of the STAT1^K193^ site reversed AARS1 overexpression-induced the promotion of STAT1 nuclear translocation, increase of STAT1 phosphorylation and enhancement of the binding between STAT1 and the ELOVL5 promoter region (Supplementary Fig. 16B–D). These findings suggest that in a high-glucose environment, AARS1 lactylates STAT1 at the K193 site, thereby increasing STAT1 transcriptional activity. Our data indicate that AARS1 modulates the lactylation of both STAT1 and H3K18 to regulate ELOVL5 transcription, thus triggering ferroptosis in DN.

### β-alanine mitigated ferroptosis in DN model mice and hyperglycaemic cells via the inhibition of AARS1-induced lactylation

**Fig. 8:**
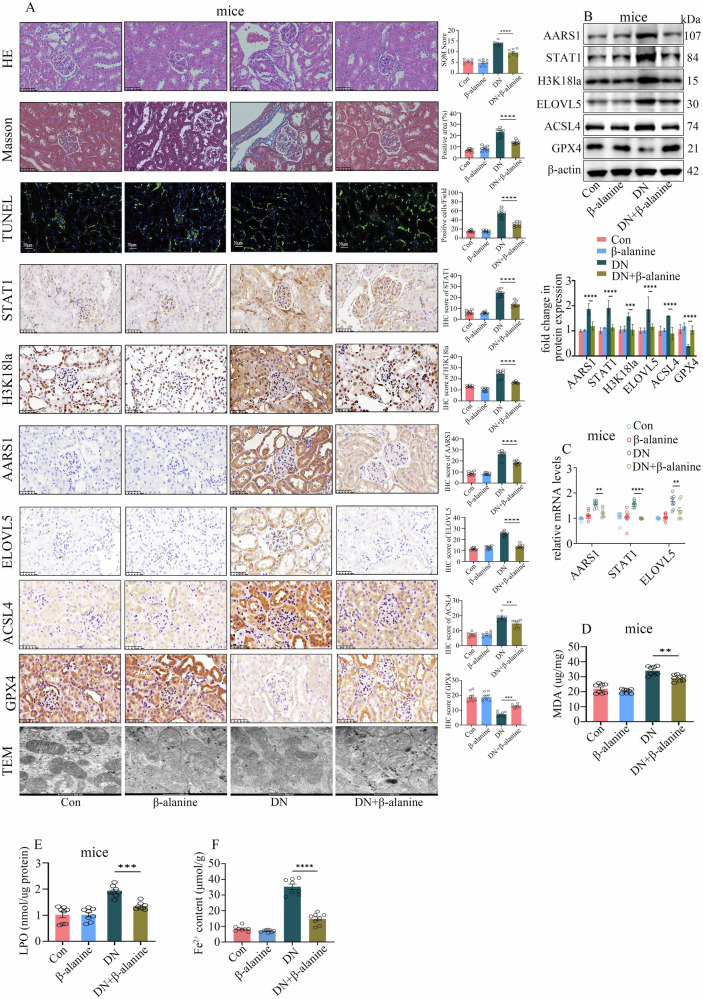
β-alanine mitigates ferroptosis in DN model mice via the inhibition of AARS1-induced lactylation. **A** Representative images of HE staining, Masson’s trichrome staining, TUNEL staining, IHC staining for AARS1, H3K18la, STAT1, ELOVL5, ACSL4, and GPX4, and transmission electron microscopy (TEM) of renal biopsy samples from the control (Con), β-alanine, DN, and DN+β-alanine model mice (scale bar: 50 μm for HE, Masson, TUNEL, and IHC; scale bar: 500 nm for TEM). Compared with DN mice, DN+β-alanine mice presented less kidney tissue damage, fibrosis, and cell death; reduced AARS1, H3K18la, STAT1, ELOVL5, and ACSL4 expression; upregulated GPX4 levels; and increased mitochondrial ridges and volumes. **B** Western blotting assays revealed that compared with DN mice, DN+β-alanine mice presented decreased AARS1, H3K18la, STAT1, ELOVL5, and ACSL4 levels and increased GPX4 expression in kidney tissues. **C** qPCR assays indicated that compared with DN mice, DN+β-alanine mice presented decreased AARS1, STAT1 and ELOVL5 mRNA expression in kidney tissues. **D** Compared with those in DN model mice, the MDA levels were decreased in the kidneys of DN+β-alanine model mice. **E** Compared with those in DN mice, LPO levels were decreased in the kidneys of DN+β-alanine model mice. **F** Compared with that in DN mice, the Fe^2+^ content was decreased in the kidneys of DN+β-alanine model mice. (*P < 0.05, **P < 0.01, ***P < 0.001, and ****P < 0.0001).

Considering that AARS1-mediated lactylation plays an important role in DN, the inhibition of AARS1-mediated lactylation may be a potential therapeutic target for DN. A previous study indicated that β-alanine can efficiently antagonize AARS1-induced lactylation by directly competing with lactate for binding to AARS1 [25]. Our in vitro lactylation study confirmed that β-alanine antagonized AARS1-induced H3K18 and STAT1 lactylation (Supplementary Fig. 17A, B). Next, β-alanine was used to treat DN models in the present study. Our data indicated that β-alanine inhibited high-glucose-induced STAT1 and H3K18 lactylation in a concentration- and time-dependent manner in hyperglycaemic cells (Supplementary Fig. 17C). Moreover, β-alanine treatment decreased AARS1, STAT1, H3K18la and ELOVL5 expression and inhibited ferroptosis in hyperglycaemic cells (Supplementary Fig. 18). Consistently, β-alanine treatment downregulated AARS1, STAT1, H3K18la and ELOVL5 expression (Fig. 8A–C), inhibited ferroptosis (Fig. 8A–F), reduced renal injury (Fig. 8A), and improved renal dysfunction (Supplementary Table 2) in DN model mice. These data indicate that inhibiting AARS1-induced lactylation via β-alanine may be an effective treatment strategy for DN.

## Discussion

The main finding of the present study was that AARS1-induced H3K18la promoted ferroptosis, thus participating in the occurrence and development of DN. Moreover, AARS1 interacted with STAT1 to trigger ferroptosis in DN via the modulation of ELOVL5 transcription. Notably, inhibiting AARS1-induced lactylation with β-alanine effectively attenuated ferroptosis, thus ameliorating renal dysfunction in DN models.

DN, which is a common and frequent microvascular complication of diabetes, has become the primary cause of end-stage renal disease worldwide [1]. Many pathophysiological disturbances, including oxidative stress, cell death, epigenetic modulation, and haemodynamic changes, occur in patients with DN [27]. Among the multiple pathophysiological disturbances, cell death has attracted increasing attention and is considered a direct factor involved in DN [28–30]. Recently, ferroptosis has been determined to play an important role in DN [10] because glomerular endothelial cells and proximal tubular epithelial cells are vulnerable to ferroptosis [11, 12]. Moreover, ferroptosis inhibition potentially exerts therapeutic effects on DN [29]. The core regulatory mechanisms of ferroptosis include iron, lipid, and cysteine metabolism [30]. Fatty acids are essential components of cellular lipid metabolism and perform important physiological functions [31]. Recently, the PUFA biosynthesis pathway has been reported to play an important role in ferroptosis [15]. In addition, the susceptibility of phospholipid-polyunsaturated fatty acids to peroxidation and their close association with ferroptosis make them key factors involved in DN [15, 16]. Therefore, the precise regulation of fatty acid metabolism, especially PUFA synthesis, is necessary to protect cells from ferroptosis [32]. In the present study, ELOVL5 expression gradually increased in patients as the DN stage progressed. Moreover, the level of ELOVL5 was also increased in hyperglycaemic cells and DN model mice. Previous studies have indicated that ELOVL5 participates in ferroptosis [15]. However, the exact mechanism by which ELOVL5 modulates ferroptosis in individuals with DN has not been studied. ELOVL5 plays crucial roles in the synthesis of ɤ-linolenic acid (18:3, n-6), dihomolinolenic acid (20:3, n-6) and arachidonic acid (20:4, n-6) [33], consistent with our lipidomic data. In addition, the inhibition of ELOVL5 attenuated ferroptosis in hyperglycaemic HGECs and HK-2 cells, which was reversed by exogenous arachidonic acid supplementation. These data indicated that ELOVL5 induced ferroptosis in hyperglycaemic HGECs and HK-2 cells by increasing the synthesis of PUFAs, such as arachidonic acid.

Epigenetic modifications have been reported to play crucial roles in the occurrence and progression of DN [18]. Our previous studies indicated that both histone methylation [19] and acetylation [20] play important roles in the development of DN. Recent studies have revealed that histone lactylation, which functions as a novel epigenetic modification, is also involved in the occurrence and development of diabetes-related complications [21, 22]. Lactate-mediated histone lactylation is involved in the regulation of gene transcription under hyperglycaemic conditions [34]. Moreover, H3K18la is a tissue-specific active enhancer [23]. These data indicate that lactate-mediated H3K18la is involved in the occurrence and progression of DN. However, little is known about the role of lactyltransferases in DN. Therefore, determining the regulatory enzymes of H3K18la is crucial to further explore the function and regulatory mechanism of H3K18la in DN. Previous studies identified AARS1 as a lactyltransferase both in vivo and in vitro [24, 25]. In the present study, the expression of AARS1 and H3K18la gradually increased in patients as the DN stage progressed. Similarly, AARS1 and H3K18la levels were increased in DN mice and hyperglycaemic cells. Moreover, the inhibition of AARS1 expression decreased H3K18la expression, attenuated ferroptosis, and ameliorated kidney injury in DN mice and hyperglycaemic cells. Consistently, AARS1 overexpression upregulated H3K18la levels and triggered ferroptosis in HGECs and HK-2 cells. However, AARS1^5M^, which lacks lactyltransferase activity, did not have these effects. Our data indicate that AARS1-mediated H3K18la plays an important role in DN. Next, we explored the exact mechanism by which AARS1-mediated H3K18la participated in ferroptosis in DN. A ChIP assay confirmed that H3K18la and AARS1 were enriched in the same region of the ELOVL5 promoter. Moreover, ferroptosis induced by AARS1 overexpression was reversed by si-ELOVL5 in HGECs and HK-2 cells. These data indicate that AARS1 induces H3K18la to modulate ELOVL5 transcription, thus participating in ferroptosis in individuals with DN.

Histone modification in the promoter region frequently coincides with transcription factor binding, thus initiating the transcription of downstream genes [35]. Through a mass spectrometry analysis, we determined that STAT1 may interact with AARS1. STAT1 is a member of the STAT family that acts as a signaling messenger and transcription factor, regulating the expression of genes associated with cell proliferation, oxidative stress, and apoptosis [36–38]. Co-IP and GST pull-down assays suggested that STAT1 could bind to AARS1. In addition, STAT1 expression gradually increased as the DN stage progressed. Moreover, STAT1 levels were increased in DN model mice and hyperglycaemic cells. Furthermore, STAT1 silencing inhibited ELOVL5 expression and attenuated ferroptosis in hyperglycaemic cells. STAT1 overexpression increased ferroptosis, which was reversed by ELOVL5 silencing. Therefore, STAT1 may hold potential therapeutic value in DN. Fludarabine is a STAT1 inhibitor that specifically blocks cytokine-induced activation of STAT1 and STAT1-dependent gene transcription [39]. Additionally, fludarabine specifically depletes STAT1 protein and mRNA levels without affecting the functions of other STATs [40]. Our cellular and animal experiments both revealed that fludarabine significantly downregulated STAT1 expression, inhibited ELOVL5 expression, inhibited ferroptosis, and ameliorated kidney dysfunction in DN models. These findings suggest that the STAT1-specific inhibitor fludarabine may be a therapeutic agent for DN.

STAT1 posttranslational modulation regulates STAT1 activity [41]. Through in vitro and in vivo lactylation experiments, we confirmed that STAT1 lactylation was regulated by AARS1. Moreover, when the level of STAT1 lactylation increased, the phosphorylation of STAT1 increased, the nuclear level of STAT1 increased, and its binding to the ELOVL5 promotor was increased upon AARS1 overexpression in cells. Further, the effects of AARS1 overexpression were similar to those of high-glucose treatment, whereas AARS1^5M^ had no effect. Further experiments revealed that the STAT1^K193^ site is the most important lactylation site regulated by AARS1. In addition, mutation of the STAT1^K193^ site reversed the activation of STAT1 transcriptional activity induced by AARS1 overexpression. Therefore, our study indicated that AARS1 affects STAT1 activity by modifying STAT1^K193^ lactylation.

Considering that AARS1 modulates the lactylation of both STAT1 and H3K18 to regulate ELOVL5 transcription and trigger ferroptosis in individuals with DN, we treated hyperglycaemic cells and DN model mice with β-alanine, which has been proven to antagonize AARS1-induced lactylation [25]. β-alanine is a limiting precursor of carnosine and is among the most commonly used sports supplements for improving athletic performance. A previous study indicated that β-alanine supplementation can increase exercise capacity in individuals with type 2 diabetes mellitus [42]. Moreover, carnosine was shown to exert nephroprotective effects via a decrease in urinary TGF-β levels and antioxidant activity [43, 44]. The present study revealed that β-alanine treatment downregulated H3K18la and ELOVL5 expression, inhibited ferroptosis, reduced renal injury, and improved renal dysfunction in DN mice and hyperglycaemic cells. Our results indicated that β-alanine may be an effective therapeutic strategy for DN via the inhibition of AARS1-induced lactylation.

This study has several limitations. First, the present study confirmed that AARS1 functions as a lactyltransferase in the regulation of H3K18la in individuals with DN; whether other lactyltransferases or delactyltransferases participate in DN also deserves further exploration. Second, an AARS1 inhibitor was not used in the animal experiments to determine its therapeutic effect on DN, which deserves further research. Third, analyzing AARS1 and H3K18la expression in DN patients stratified by eGFR, with diabetic patients without DN as controls, would provide more insightful results. In future studies, renal tissue samples from diabetic patients without DN should be used as the control to further elucidate the underlying mechanisms. Fourth, the current study exclusively enrolled patients with stage 2, 3, and 4 DN. Future investigations should strive to include patients from all stages of the disease to enhance the generalizability and comprehensiveness of the findings.

## Conclusions

Our data indicated that ELOVL5 modulates PUFA production to trigger ferroptosis in individuals with DN. Moreover, AARS1 acts as a lactyltransferase to modulate H3K18 and STAT1^K193^ lactylation to modulate ELOVL5 transcription, thus mediating ferroptosis in individuals with DN. In addition, the inhibition of AARS1-induced lactylation may be an effective treatment strategy for DN.

## Methods and materials

### Subjects

This study was conducted in accordance with the Declaration of Helsinki and received approval from the Huzhou Central Hospital Ethics Committee (Ethics number: 20191209-01). Patients diagnosed with DN participated in this study, and informed consent was obtained from all participants. The exclusion criteria included patients with advanced liver disease, renal failure, stroke, cancer, immune-related diseases, peripheral arterial disease, and other cardiovascular diseases. The estimated glomerular filtration rates (eGFRs) of the participants were calculated using the Chronic Kidney Disease Epidemiology Collaboration (CKD-EPI) formula. The classification of DN severity was categorized into stages 2, 3, or 4 based on the eGFR.

### Mouse DN model

The study received approval from the Shanghai First People’s Hospital Clinical Center Laboratory Animal Welfare and Ethics Committee (Ethical Approval Number: 2023AWS150). The mice were randomly allocated to the groups in this study. No blinding was performed in the present animal study. AARS1 heterozygous mice (AARS1^+/–^) on a C57BL/6 background were obtained from Jicui Biotechnology Limited (Jiangsu, China). Age-matched male wild-type and AARS1^+/–^ littermates that we bred were utilized at 2 months of age. The DN (n = 8) and AARS1^+/–^ DN (n = 8) groups were established using a previously described method [20]. Briefly, the mice underwent unilateral nephrectomy while they were anaesthetized following an intraperitoneal injection of 80 mg/kg ketamine and 5 mg/kg xylazine. Following the procedure, the mice were returned to the care facility for 15 weeks. Three weeks after nephrectomy, the mice that received an intraperitoneal injection of citrate buffer (0.1 M, pH 4.5) for 7 consecutive days were assigned to the control group (Con, n = 8). The DN group was placed on a high-sugar and high-fat diet for 15 weeks starting after nephrectomy. This dietary regimen was supplemented with an intraperitoneal injection of streptozotocin (STZ, 50 mg/kg for 7 consecutive days; HY-13753, MCE, China), which was administered for three weeks after nephrectomy. The DN model mice that were intraperitoneally injected with 0.65 mg/kg ferrostatin-1 (HY-100579, MCE, China), which was derived from one previous study [45], once a day after STZ injection were designated the DN+Fer-1 group (n = 8). The mice that were intraperitoneally injected with the STAT1 inhibitor fludarabine (HY-B0069, MCE, China) at 30 mg/kg, which was derived from one previous study [46], three times a week after the STZ injection were designated the DN+Flu group (n = 8). Finally, a 2.4 g/day dose of β-alanine has been demonstrated to enhance physical work capacity in humans [47]. When this dosage was adjusted for mice using the FDA-recommended conversion factor, it equated to approximately 418 mg/kg/day [48]. Prior research has indicated that a 4 g/day dose of β-alanine supplementation can significantly increase exercise capacity in individuals with Type 2 Diabetes Mellitus [42], which is roughly double the aforementioned dose [47, 48]. Therefore, the target dose for β-alanine in this study was set at 836 mg/kg/day. The mice that were orally administered β-alanine (HY-N0230, MCE, China) daily after the intraperitoneal injection of citrate buffer or STZ were designated the β-alanine group (n = 8) or DN+β-alanine group (n = 8).

### Cell culture and intervention

HGECs and HK-2 cells were obtained from Procell (Procell, China) and cultured in DMEM (CM-H061, CM-0109, Procell, China) for serial subcultivation. The cells cultured in normal glucose (5 mM) for 6 days were designated the control group (Con). The cells cultured in high-glucose medium (25 mM) for 6 days were designated the high-glucose (HG) group. Glucose (5 mM) plus mannitol (20 mM) was used as an osmotic control (mannitol) to mitigate the effects of elevated osmotic pressure resulting from the high-glucose treatment of cells. The AARS1 inhibitor Gln-AMS (HY-112861, MCE, China) was used in the cellular experiments. The cells were incubated with different concentrations of Gln-AMS for different time (24, 48, or 72 h). The optimal treatment concentration and duration for the significant inhibitory effects of Gln-AMS on 25 mM glucose-induced AARS1 expression were determined. Arachidonic acid (AA, 2.5 μM; 90010; Cayman Chemical, MI, USA) was used to treat si-ELOVL5 cells cultured with high glucose. Moreover, Fer-1 (HY-100579, MCE, China) was used to inhibit high glucose-mediated ferroptosis in cells. Furthermore, β-alanine (HY-N0230, MCE, China) was used to treat the cells in this study to inhibit AARS1-induced lactylation. In addition, fludarabine (HY-B0069, MCE, China) was used to inhibit STAT1 level. The optimal treatment concentration and duration for the significant effects of Fer-1, Flu, and β-alanine was determine. shRNAs, siRNAs, and plasmids were transfected into HGECs and HK-2 cells according to the manufacturer’s instructions. The amount of plasmid, siRNA, or shRNA relative to Lipofectamine 2000 reagent was 1 mg per 1.2 ml. A mutant AARS1^5M^ plasmid carrying a mutant form of AARS1, which loses lactyltransferase activity, was constructed [24]. An AARS1^5M^ overexpression plasmid (AARS1^5M^-OE) was used to verify the importance of AARS1-mediated lactylation in the modulation of ELOVL5 transcription and ferroptosis. To further elucidate the critical role of AARS1-mediated STAT1 lactylation in STAT1 transcriptional function, mutations in the STAT1 lactylation sites (K193, K209 and K685) were constructed, as reported in a previous study [26]. The sequences of si-STAT1 (Biotend, Shanghai, China) were as follows: siRNA-a, 5ʹ-CTGGATATATCAAGACTGA-3ʹ; and siRNA-b, 5ʹ-CCCUAGAAGACUUACAAGAUGAAU-3ʹ. The sequences of si-ELOVL5 (Biotend, Shanghai, China) were as follows: siRNA-a, 5ʹ-GGAACATTTTGATGCATCA-3ʹ; and siRNA-b, 5ʹ-GTACTACTTCTCCAAACTCAUTT-3ʹ. The sequences of sh-AARS1 (Biotend, Shanghai, China) were as follows: shRNA-a, 5ʹ-GGTGGATGACAGCAGTGAAGA-3ʹ; and sh-RNA-b, 5ʹ-GGACATCATTAATGAAGAAGA-3ʹ.

### Western blotting, immunoprecipitation, GST pull-down assays, Immunofluorescence (IF) staining and Quantitative real-time PCR (qPCR)

The Western blotting, immunoprecipitation, GST pull-down, IF and qPCR analysis was performed as described previously [19]. The primary antibodies used in this study are listed in Supplementary Table 8. For GST pull-down assay, His-AARS1 (Ag11151, Proteintech, Shanghai, China) and GST-STAT1 (Ag0199, Proteintech, Wuhan, China) fusion proteins were used in this study. For the IF assay, the nuclei were stained with DAPI (4083S; Cell Signaling Technology, MA, USA), and the endoplasmic reticulum was stained with ER-Tracker (C1041S, Beyotime, China). The sequences of the qPCR primers used in this study are listed in Supplementary Table 9.

### In vitro lactylation assay

The in vitro lactylation assay was performed by incubating the purified HIS-AARS1 protein (Ag11151, Proteintech, Shanghai, China) with recombinant human GST-STAT1 (1 μg, Ag0199, Proteintech, Wuhan, China) or GST-H3 (1 μg, ab159999, Abcam, Cambridge, UK) along with lactate (5 mM; HY-B2227, MCE, China) in incubation buffer (50 mM HEPEs, 25 mM KCl, 2 mM MgCl2, 4 mM ATP, pH 7.5) for 3 h at 37 °C. Lactate-mediated lactylation was inhibited by administering 2 mM or 10 mM β-alanine in in vitro experiments. The samples were subsequently denatured at 95 °C for 5 min and analysed via Western blotting.

### Haematoxylin-eosin (HE) staining, Masson’s trichrome staining, and immunohistochemistry (IHC)

The HE staining was performed as described previously [19]. Images of the tissues were captured using a bright-field microscope. Two independent observers, blinded to the experimental groups, evaluated kidney injury using a scoring system based on the extent of damage. Scores were assigned for four parameters: glomerular endothelial cell proliferation index, destruction of glomerular capillary collateral activity index, mesangial cell hyperplasia index, and renal interstitial inflammatory cell infiltration index. Each parameter was graded on a scale from 0 to 4 (0: 0%; 1: <10%; 2: 10–25%; 3: >25–50%; 4: >50%), and the total kidney injury score was obtained by summing these scores. Five non-overlapping fields were analyzed for each section, and the mean score across all sections within each group was used as the final kidney injury score.

The Masson’s trichrome staining and the IHC analysis was performed as described previously [19]. The primary antibodies used in the study are listed in Supplementary Table 8.

### Transmission electron microscopy (TEM)

TEM analysis was performed to observe ultrastructural changes. The samples were fixed with 2.5% glutaraldehyde in 0.1 M phosphate buffer (pH 7.4) at 4 °C overnight. After rinses with phosphate buffer, the samples were postfixed with 1% osmium tetroxide for 2 h, dehydrated in a graded ethanol series, and embedded in epoxy resin. Ultrathin sections (70–90 nm) were cut using an ultramicrotome, mounted on copper grids, and stained with 2% uranyl acetate and lead citrate. Images were acquired using a transmission electron microscope operated at an acceleration voltage of 80–120 kV.

### Malondialdehyde (MDA) and lipid peroxidation (LPO) assays

MDA levels were measured using a lipid peroxidation MDA assay kit (S0121S, Beyotime, China). LPO levels were determined with an LPO assay kit (Solarbio, Beijing, China). The protein concentrations of the cell and kidney lysates were measured using a BCA protein assay kit (P0010S, Beyotime, China). The ratios of MDA and LPO to the protein concentration were calculated.

### Lipid peroxidation analysis via C11-BODIPY 581/591 staining

The C11-BODIPY 581/591 (MX5211-1MG; Shanghai Maokang Biotechnology, Shanghai, China) staining analysis was performed as described previously [19].

### Fe^2+^ detection

The intracellular Fe^2+^ levels were assessed using the FeRhoNox-1 fluorescent probe (MX4558; Maokang Biotech, China), followed by nuclear counterstaining with Hoechst 33342 (C1029; Beyotime, China). Imaging was performed with a confocal microscope. Tissue Fe^2+^ levels were measured using a commercial Iron Assay Kit according to the manufacturer’s instructions (BC5415, Solarbio, China). Fresh tissues were homogenized in assay buffer, and the supernatant was collected after centrifugation. The reaction mixture was prepared with the detection reagent and incubated at room temperature. The Fe^2+^ levels were quantified by measuring the absorbance with a microplate reader and calculated using a standard curve.

### Mitochondrial membrane potential (MMP) detection

The mitochondrial membrane potential was measured using a JC-1 assay kit (C2003S, Beyotime, China) according to the manufacturer’s protocol. The cells were incubated with JC-1 staining solution at 37 °C for 20 min, followed by two washes with JC-1 buffer solution. The fluorescence signals of the JC-1 aggregates and monomers were captured using a confocal microscope at excitation/emission wavelengths of 525/590 nm and 490/530 nm, respectively. Hoechst 33342 (C1029; Beyotime, China) was used for nuclear counterstaining.

### MitoSOX™ red staining

The cells were stained with a 5 μM MitoSOX™ Red (HY-D1055, MCE, USA) working solution at 37 °C for 10–20 min in the dark. After staining, the cells were rinsed three times with PBS to eliminate the excess dye and subsequently counterstained with Hoechst 33342 (C1029, Beyotime, China) to label the nuclei. The mitochondrial superoxide levels were visualized using a fluorescence microscope with excitation at 510 nm and emission at 580 nm.

### PI and TUNEL assays

A PI kit (A211-01, Vazyme Biotech, China) was used to label early-stage dead cells as green and late-stage cells as red according to the manufacturer’s instructions. The images were captured with a fluorescence microscope.

The biopsy tissue was fixed at room temperature for 30 min and then washed with PBS before being immersed in 0.2% Triton X-100 buffer for 5 min. After washes with PBS and drying, 50 μL of the reaction mixture was added to each section, and the sections were subsequently placed in an incubator at 37 °C for 60 min according to the protocol of the In Situ Cell Death Detection Kit (TMR Red; 12156792910, Billerica, MA, USA). For the labeling of endothelial cells, kidney sections were incubated with a CD31 antibody (11265-1-AP; ProteinTech, Wuhan, China) at 4 °C overnight and then incubated with a fluorescent dye-conjugated secondary antibody (111-545-003; Jackson ImmunoResearch Laboratories, PA, USA) after washes with PBS.

### Chromatin immunoprecipitation (ChIP), re-ChIP, ChIP-qPCR and Dual-luciferase assay

The ChIP, re-ChIP, ChIP-qPCR and Dual-luciferase analysis was performed as described previously [19]. The ChIP sequences of the primers used in this study are listed in Supplementary Table 10.

### Pathway enrichment analysis

The Kyoto Encyclopedia of Genes and Genomes (KEGG) database was used to identify the enriched pathways of the differentially expressed genes. A two-tailed Fisher’s exact test was used in this analysis. These pathways were classified into hierarchical categories according to the KEGG website.

### Statistical analysis

Eight separate in vivo experiments and 5 separate in vitro experiments were conducted in this study, and statistical significance was determined using GraphPad Prism 8 Project software. The sample sizes of in vivo and in vitro experiments were determined by assessing high glucose- or hyperglycemia-induced H3K18la expression in pilot experiments. The outcomes are presented as the means ± standard deviations (SDs), and comparisons of the means of the two groups were conducted by two-tailed unpaired t tests. One-way ANOVA followed by Bonferroni-corrected pairwise comparisons were used to compare the means of more than 2 groups. A *P* value less than 0.05 was considered to indicate statistical significance. The figures were generated using GraphPad Prism 8.

## Supplementary information

supplemental figures

supplemental table 1

supplemental table 2

supplemental table 3

supplemental table 4

supplemental table 5

supplemental table 6

supplemental table 7

supplemental table 8

supplemental table 9

supplemental table 10

original WB blot

gray value of WB data

## Data Availability

The datasets used and/or analysed during the current study are available from the corresponding author on reasonable request.
