# Supplementary material for: AARS1-mediated lactylation of H3K18 and STAT1 promotes ferroptosis in diabetic nephropathy
Source: Cell Death Differ. 2025 Sep 23;33(3):589–604. doi: 10.1038/s41418-025-01587-4 (PMC13036035; doi:10.1038/s41418-025-01587-4)
Supplement: Supplementary file 1 — supplemental figures [file 41418_2025_1587_MOESM1_ESM.pdf]

Supplementary material

Supplementary Figure 1. AARS1 inhibition attenuates high-glucose-induced AARS1 and H3K18la expression, as well as cell death in HGECs and HK-2 cells

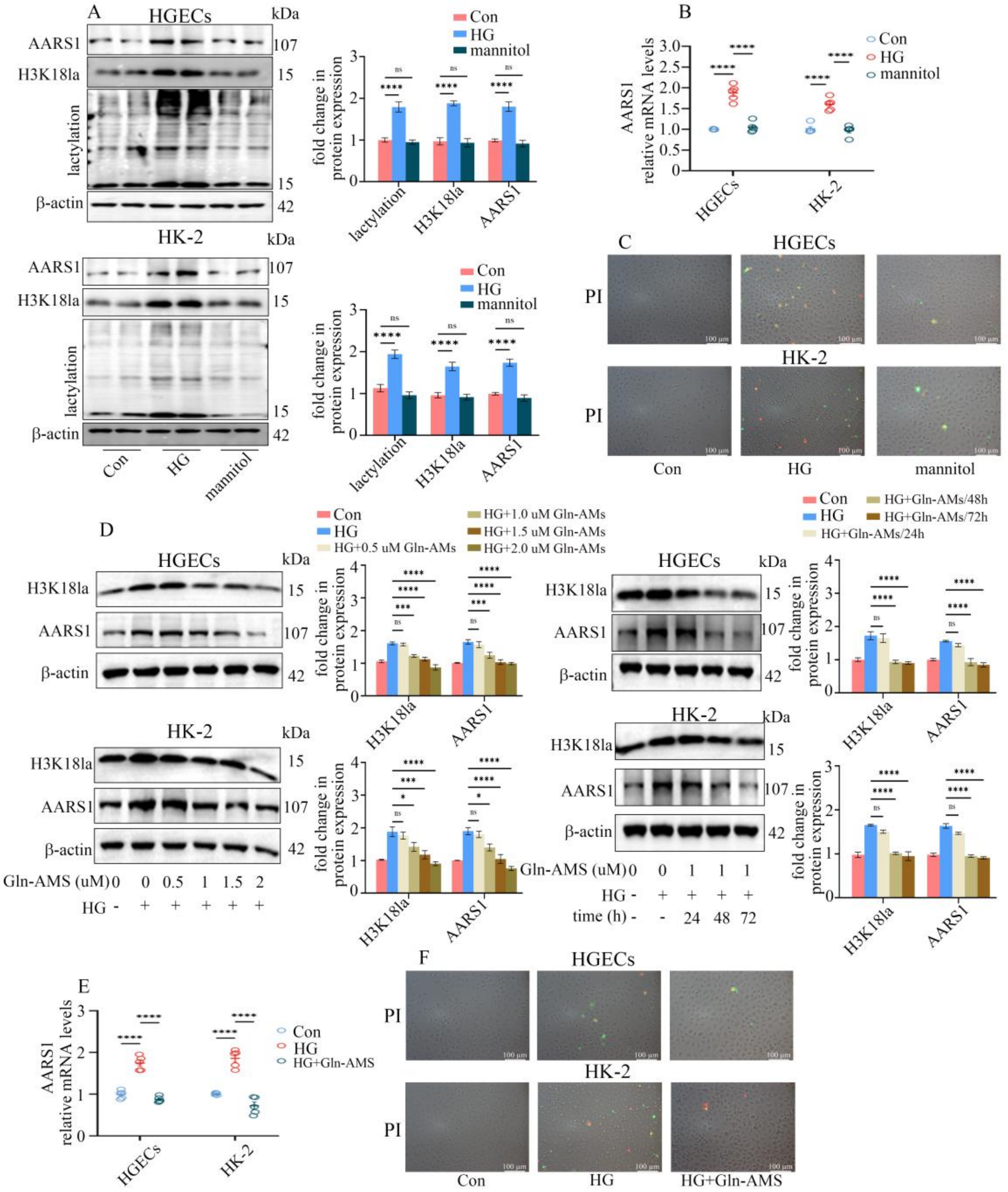

(A) Western blotting assays indicated that the levels of AARS1, lactylation and H3K18la were increased in hyperglycaemic cells. (B) qPCR assays indicated that the AARS1 mRNA levels were increased in hyperglycaemic cells. (C) A PI assay

indicated that high glucose concentrations increased the death of HGECs and HK-2 cells (scale bar: 100 μm). (D) Western blotting assays revealed that the levels of AARS1 and H3K18la were increased in hyperglycaemic cells, and these changes were reversed by the AARS1 inhibitor Gln-AMS in a concentration- and time-dependent manner. Incubation of cells with 1 μM Gln-AMS for 72 hours caused a significant reversal of high-glucose-induced AARS1 and H3K18la levels. (E) qPCR revealed that the AARS1 mRNA level was increased in hyperglycaemic cells, which was reversed by 1 μM Gln-AMS treatment. (F) A PI assay indicated that high glucose concentrations increased the death of HGECs and HK-2 cells, which was reversed by 1 μM Gln-AMS treatment (scale bar: 100 μm). (*P<0.05, **P<0.01, ***P<0.001, and ****P<0.0001.)

Supplementary Figure 2. Ferrostatin-1 (Fer-1) attenuates kidney injury in diabetic nephropathy (DN) model mice

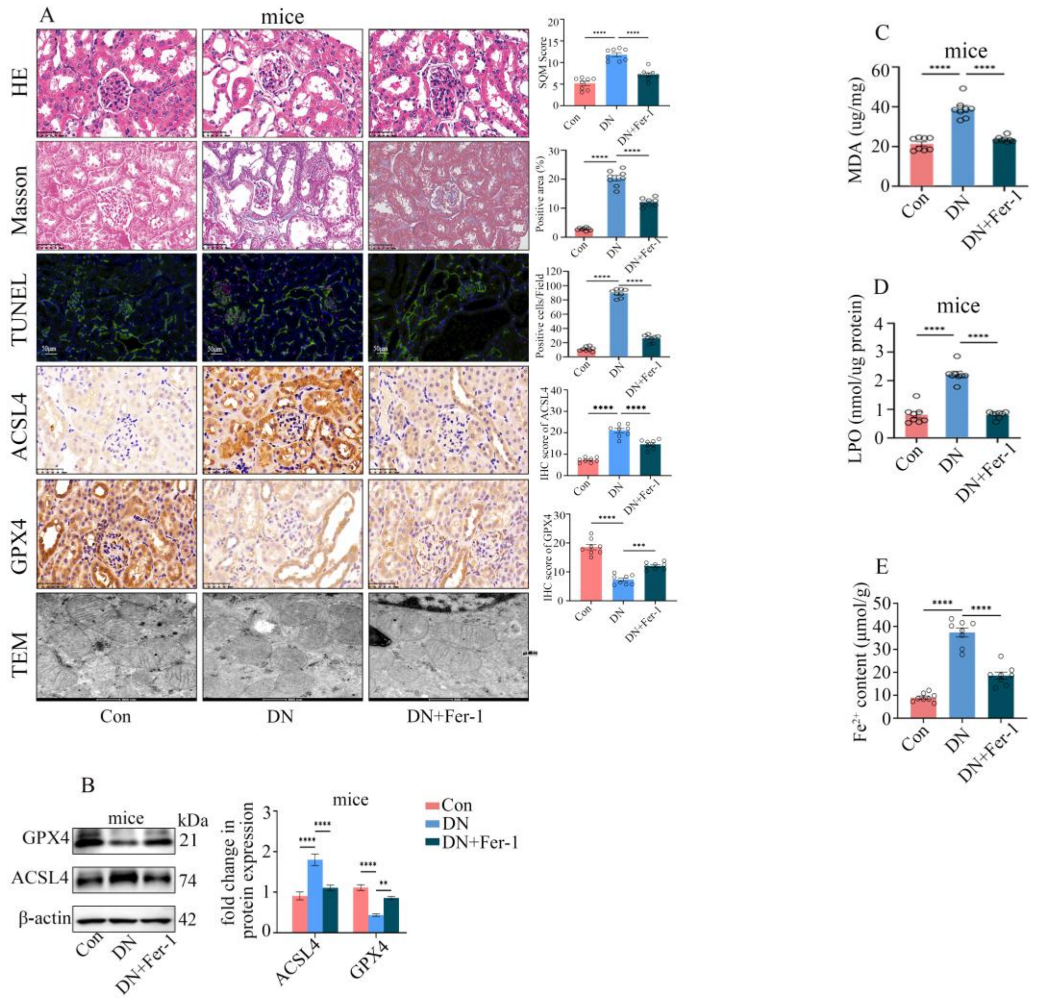

(A) Representative images of HE staining, Masson's trichrome staining, TUNEL staining, IHC staining for ACSL4 and GPX4, and transmission electron microscopy (TEM) of renal biopsy samples from the control (Con), DN and DN+Fer-1 mice used in the present study (scale bar: 50 μm for HE, Masson, TUNEL and IHC; scale bar: 500 nm for TEM). Compared with DN mice, Fer-1-treated mice presented less damage to the kidney tissue structure, alleviated fibrosis, decreased kidney cell death, reduced ACSL4 expression, upregulated GPX4 levels and increased mitochondrial ridges and mitochondrial volumes. (B) Western blotting assays indicated that Fer-1

treatment decreased the protein level of ACSL4 but increased the GPX4 level in kidney tissue from DN mice. (C) Malondialdehyde (MDA) levels were increased in the kidneys of DN mice, and this increase was reversed via Fer-1 treatment. (D) Lipid peroxidation (LPO) levels were increased in the kidneys of DN mice, and this increase was counteracted by the Fer-1 treatment. (E) The Fe^{2+} content was increased in the kidneys of DN mice, which was counteracted via Fer-1 treatment. (*$P<0.05$, **$P<0.01$, ***$P<0.001$, and ****$P<0.0001$.)

Supplementary Figure 3. Ferrostatin-1 (Fer-1) attenuates high-glucose-induced ferroptosis in HGECs and HK-2 cells

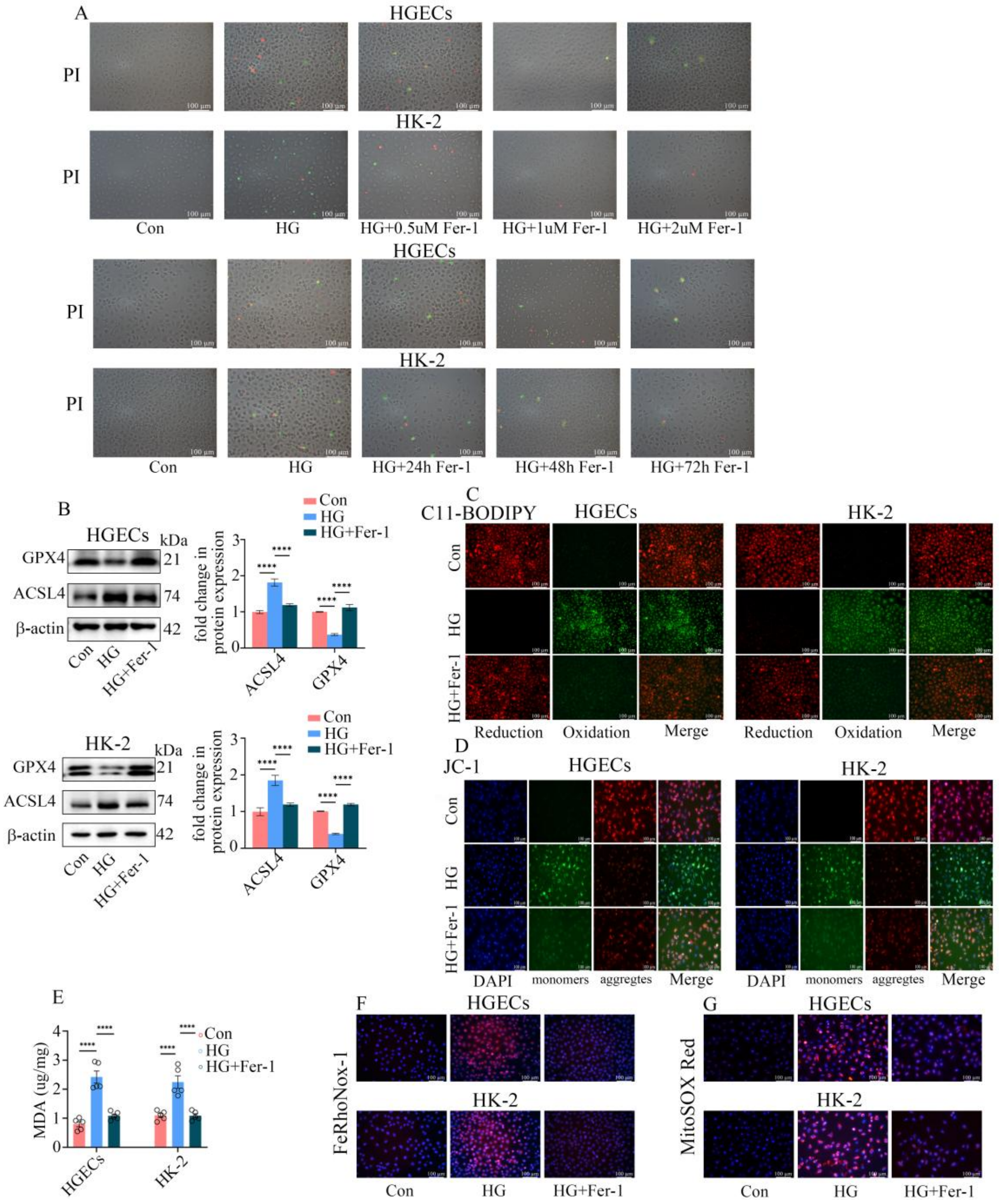

(A) A PI assay indicated that the high-glucose treatment increased the death of HGECs and HK-2 cells, which was reversed by the Fer-1 treatment in a time- and concentration-dependent manner (scale bar: 100 μm). Incubation of cells with 1 μM Fer-1 for 72 hours caused a significant reversal of high-glucose-mediated cell death.

(B) Western blotting assays indicated that Fer-1 treatment decreased the ACSL4 protein level but increased the GPX4 level in hyperglycaemic cells. (C) The C11-BODIPY 581/591 fluorescent probe was used to detect lipid peroxidation levels in HGECs and HK-2 cells. The results indicated that Fer-1 treatment decreased lipid peroxidation levels in hyperglycaemic cells (scale bar: 100 μm). (D) The JC-1 fluorescent probe was used to detect changes in the mitochondrial membrane potential (MMP) in HGECs and HK-2 cells. Our results showed that the probes in hyperglycaemic cells were mainly in the form of green fluorescent monomers. In contrast, after the Fer-1 intervention, the probes were converted into red fluorescent polymers, indicating that the Fer-1 treatment attenuated the destruction of the MMP in hyperglycaemic cells (scale bar: 100 μm). (E) Malondialdehyde (MDA) levels were increased in hyperglycaemic HGECs and HK-2 cells, and this increase was reversed by the Fer-1 treatment. (F) A FeRhoNox-1 fluorescent probe was used to detect the Fe^{2+} content in the cells. Our results showed that FeRhoNox-1 fluorescence was increased in hyperglycaemic cells but was decreased by Fer-1 treatment. These data indicated that the Fe^{2+} content was increased in hyperglycaemic cells, which was reversed by Fer-1 treatment (scale bar: 100 μm). (G) The red fluorescence intensity of MitoSOX, a mitochondria-specific superoxide indicator, was significantly increased in hyperglycaemic cells but was reversed by Fer-1 treatment (scale bar: 100 μm). (*$P<0.05$, **$P<0.01$, ***$P<0.001$, and ****$P<0.0001$.)

Supplementary Figure 4. AARS1 silencing attenuates high-glucose-induced ferroptosis in HGECs and HK-2 cells

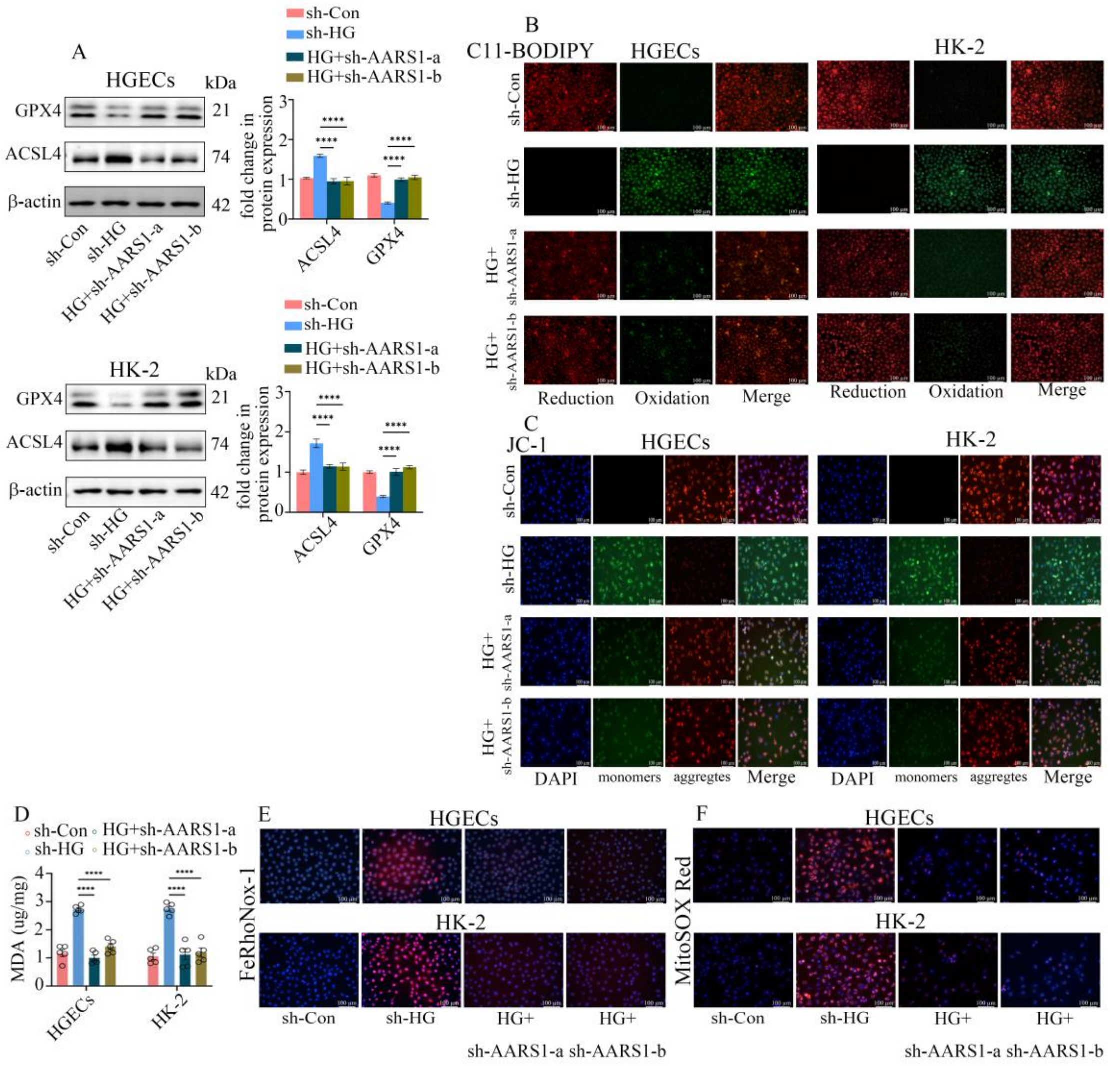

(A) Western blotting assays indicated that AARS1 silencing decreased the ACSL4 protein level but increased the GPX4 level in hyperglycaemic cells. (B) The C11-BODIPY 581/591 fluorescent probe was used to detect lipid peroxidation levels in HGECs and HK-2 cells. These results indicated that AARS1 silencing decreased lipid peroxidation levels in hyperglycaemic cells (scale bar: 100 μm). (C) The JC-1 fluorescent probe was used to detect changes in the mitochondrial membrane potential (MMP) in HGECs and HK-2 cells. Our results revealed that the probes in the high-

glucose treatment group were mainly green fluorescent monomers. In contrast, after the intervention with sh-AARS1, they were converted into red fluorescent polymers, indicating that AARS1 silencing attenuated the destruction of the MMP in hyperglycaemic cells (scale bar: 100 μm). (D) Malondialdehyde (MDA) levels were increased in hyperglycaemic HGECs and HK-2 cells, which was reversed by AARS1 silencing. (E) A FeRhoNox-1 fluorescent probe was used to detect the Fe^{2+} content in cells subjected to the corresponding treatments. Our results revealed that FeRhoNox-1 fluorescence was increased in hyperglycaemic cells but was decreased by the sh-AARS1 treatment. These data indicated that the Fe^{2+} content was increased in hyperglycaemic cells, but these changes were reversed by AARS1 silencing (scale bar: 100 μm). (F) The red fluorescence intensity of MitoSOX, a mitochondria-specific superoxide indicator, was significantly increased in hyperglycaemic cells, which was reversed by AARS1 silencing (scale bar: 100 μm). (*P<0.05, **P<0.01, ***P<0.001, and ****P<0.0001.)

Supplementary Figure 5. AARS1 inhibition attenuates high-glucose-induced ferroptosis in HGECs and HK-2 cells

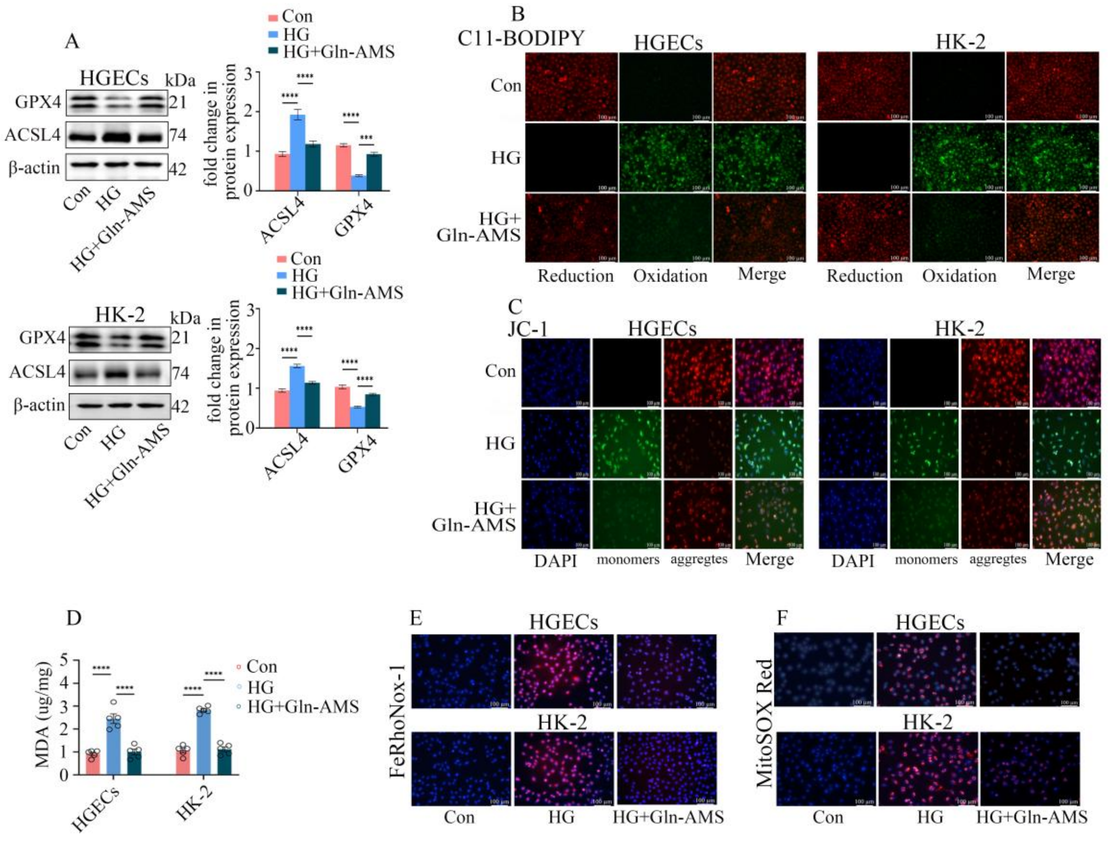

(A) Western blotting assays indicated that Gln-AMS treatment decreased the ACSL4 protein levels but increased GPX4 levels in hyperglycaemic cells. (B) The C11-BODIPY 581/591 fluorescent probe was used to detect lipid peroxidation levels in HGECs and HK-2 cells. The results indicated that the AARS1 inhibitor Gln-AMS decreased lipid peroxidation levels in hyperglycaemic cells (scale bar: 100 μm). (C) The JC-1 fluorescent probe was used to detect changes in the mitochondrial membrane potential (MMP) in HGECs and HK-2 cells subjected to the corresponding treatments. Our results revealed that the probes in the high-glucose treatment group were mainly green fluorescent monomers. In contrast, after the intervention with Gln-

AMS, they were converted into red fluorescent polymers, indicating that Gln-AMS treatment attenuated the destruction of the MMP in hyperglycaemic cells (scale bar: 100 μm). (D) Malondialdehyde (MDA) levels were increased in hyperglycaemic HGECs and HK-2 cells, which was reversed by the Gln-AMS treatment. (E) A FeRhoNox-1 fluorescent probe was used to detect the Fe^{2+} content in cells subjected to the corresponding treatments. Our results showed that FeRhoNox-1 fluorescence was increased in hyperglycaemic cells but was decreased by the Gln-AMS treatment. These data indicated that the Fe^{2+} content was increased in hyperglycaemic cells, which was reversed by the Gln-AMS treatment (scale bar: 100 μm). (F) The red fluorescence intensity of MitoSOX, a mitochondria-specific superoxide indicator, was significantly increased in hyperglycaemic cells, which was reversed by the Gln-AMS treatment (scale bar: 100 μm). (*P<0.05, **P<0.01, ***P<0.001, and ****P<0.0001.)

Supplementary Figure 6. The expression of ELOVL5, whose promoter is enriched with H3K18la, in diabetic nephropathy (DN) patients and DN models

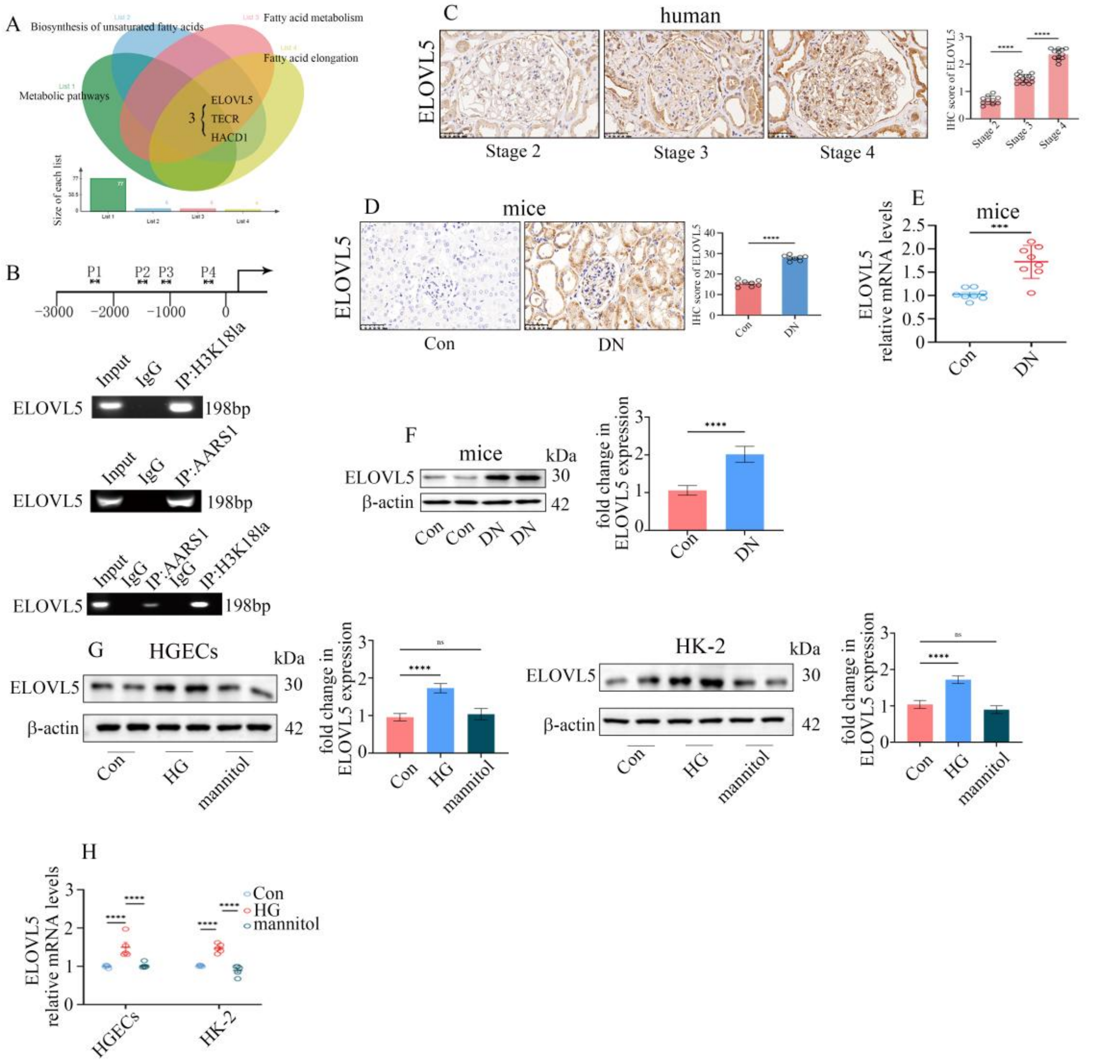

(A) Venn diagram of the genes involved in the biosynthesis of unsaturated fatty acids, fatty acid metabolism, fatty acid elongation and metabolic pathways. (B) ChIP assays verified that AARS1 and H3K18la were both enriched in the same region of the ELOVL5 promoter. (C) IHC data indicating that ELOVL5 levels in renal biopsy samples from DN patients gradually increased as the DN stage progressed (scale bar: 50 μm). (D) Representative images of IHC staining for ELOVL5 in renal biopsy samples from control (Con) and DN mice (scale bar: 50 μm). (E) qPCR assays

indicated that the ELOVL5 mRNA level was increased in the kidneys of DN mice. (F) Western blotting assays indicated that the ELOVL5 protein level was increased in the kidneys of DN mice. (G) Western blotting assays indicated that the ELOVL5 protein level was increased in hyperglycaemic cells. (H) qPCR assays indicated that the ELOVL5 mRNA level was increased in hyperglycaemic cells. (*P<0.05, **P<0.01, ***P<0.001, and ****P<0.0001.)

Supplementary Figure 7. ELOVL5 silencing inhibits high-glucose-induced ferroptosis in HGECs and HK-2 cells

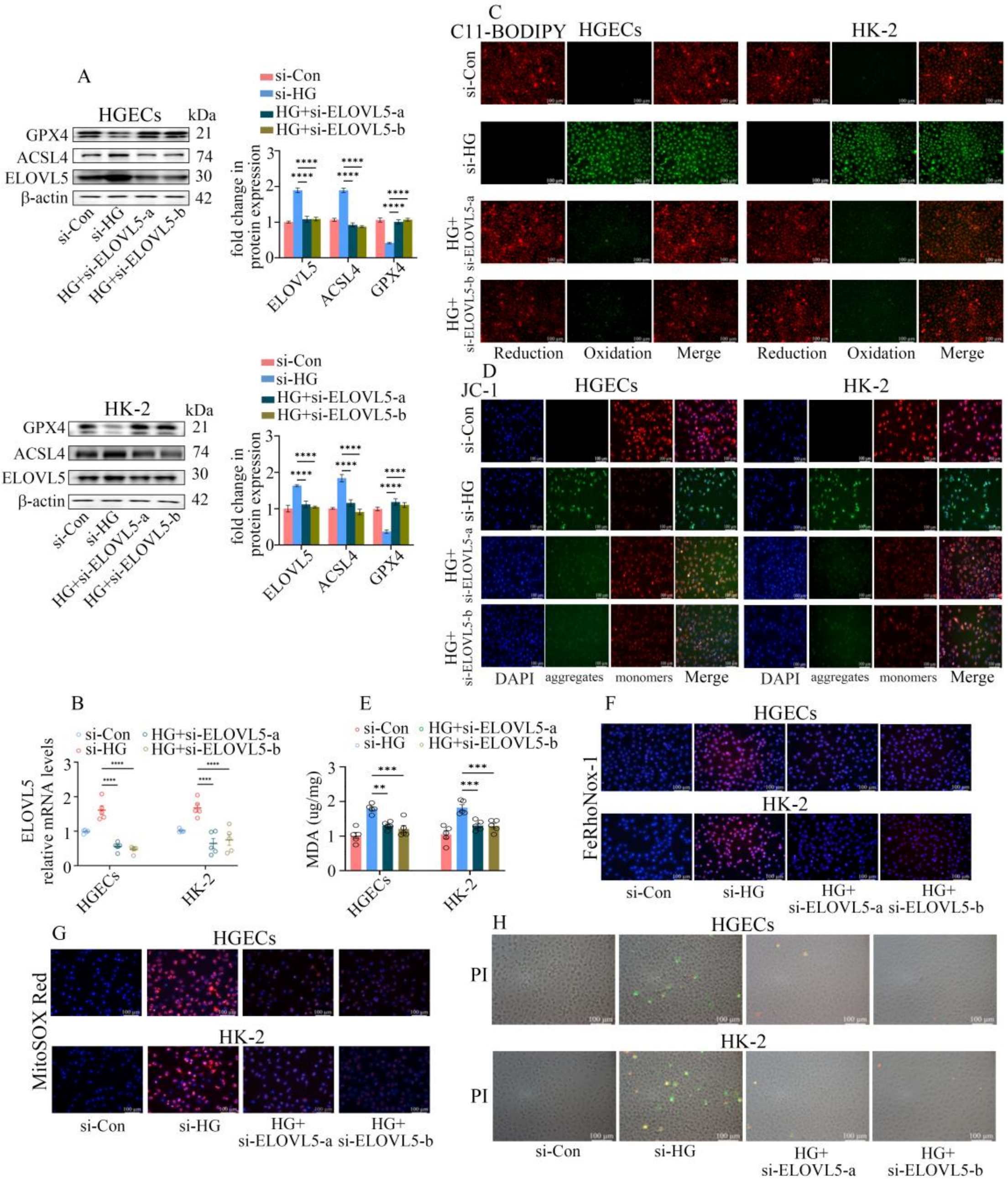

(A) Western blotting assays indicated that ELOVL5 silencing reversed the high-glucose-mediated increase in ACSL4 expression and decrease in GPX4 expression. (B) Results of the qPCR analysis of ELOVL5 levels in HGECs and HK-2 cells after the corresponding treatments. (C) The C11-BODIPY 581/591 fluorescent probe was used

to detect lipid peroxidation levels in HGECs and HK-2 cells. The results indicated that high glucose concentrations increased lipid peroxidation levels, which were reversed by ELOVL5 silencing in cells (scale bar: 100 μm). (D) The JC-1 fluorescent probe was used to detect changes in the mitochondrial membrane potential (MMP) in HGECs and HK-2 cells subjected to the corresponding treatments. Our results revealed that the probes in high-glucose-treated cells were mainly green fluorescent monomers. In contrast, after the intervention with si-ELOVL5, they were converted into red fluorescent polymers, indicating that ELOVL5 silencing attenuated the destruction of the MMP in hyperglycaemic cells (scale bar: 100 μm). (E) Malondialdehyde (MDA) levels were increased in hyperglycaemic cells, and these changes were reversed by ELOVL5 silencing. (F) A FeRhoNox-1 fluorescent probe was used to detect the Fe^{2+} content in cells subjected to the corresponding treatments. Our results revealed that FeRhoNox-1 fluorescence was increased in hyperglycaemic cells but was decreased upon ELOVL5 silencing. These data indicated that the Fe^{2+} content was increased in hyperglycaemic cells, which was reversed by ELOVL5 silencing (scale bar: 100 μm). (G) The red fluorescence intensity of MitoSOX, a mitochondria-specific superoxide indicator, was significantly increased in hyperglycaemic cells, which was reversed by ELOVL5 silencing (scale bar: 100 μm). (H) A PI assay indicated that the high-glucose treatment increased cell death, which was reversed by ELOVL5 silencing (scale bar: 100 μm). (*P<0.05, **P<0.01, ***P<0.001, and ****P<0.0001.)

Supplementary Figure 8. ELOVL5 mediates ferroptosis by regulating PUFA synthesis in hyperglycaemic HGECs and HK-2 cells

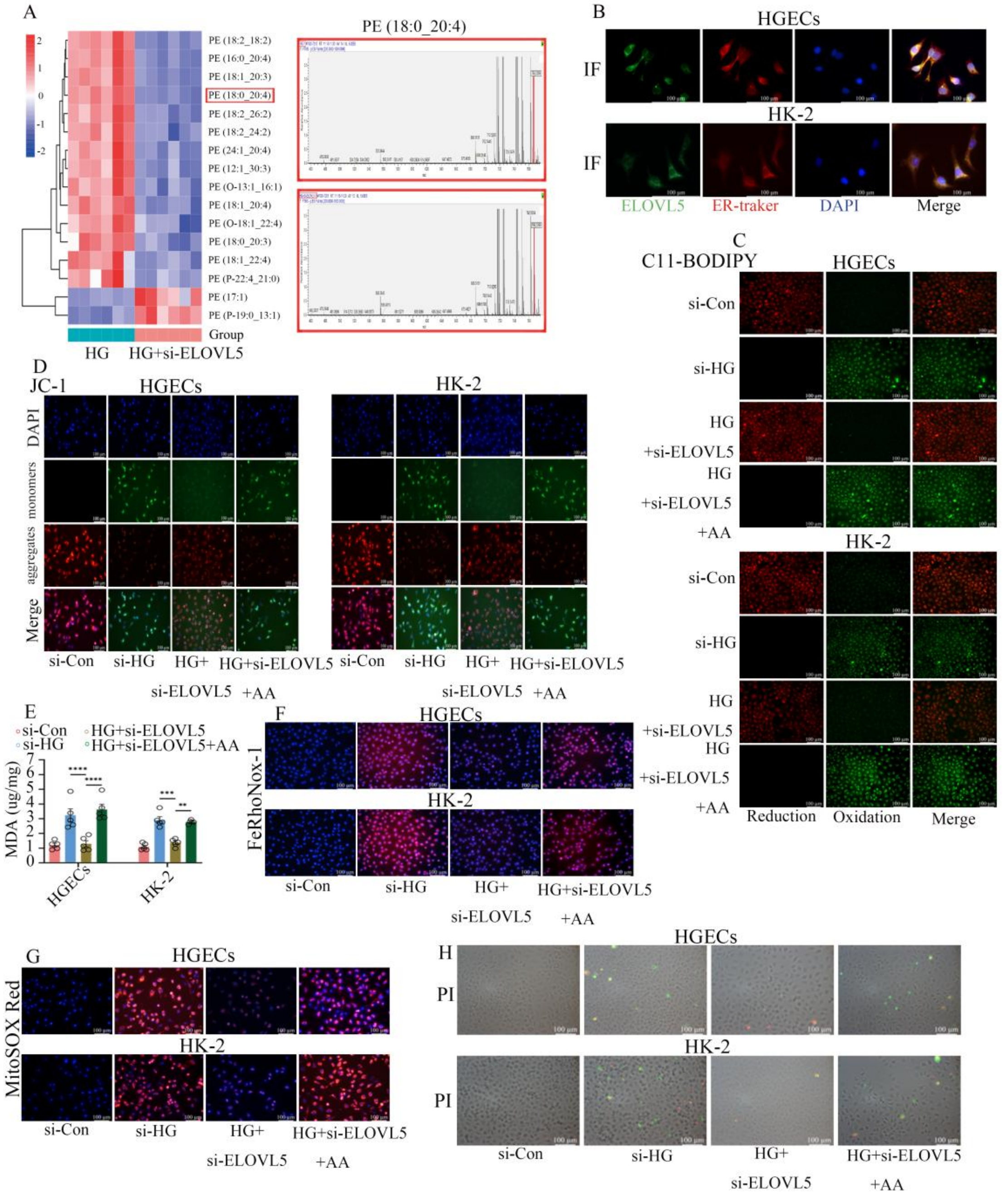

(A) ELOVL5-related lipid metabolism in HGECs was identified by mass spectrometry. (B) Immunofluorescence (IF) staining indicated that ELOVL5 was located mainly in the endoplasmic reticulum in cells (scale bar: 100 μm). (C) The C11-BODIPY 581/591 fluorescent probe was used to detect lipid peroxidation levels

in HGECs and HK-2 cells. The results indicated that incubating ELOVL5-silencing cells with AA reversed the protective effect of si-ELOVL5 on high-glucose-induced lipid peroxidation in cells (scale bar: 100 μm). (D) The JC-1 fluorescent probe was used to detect changes in the mitochondrial membrane potential (MMP) of HGECs and HK-2 cells subjected to the corresponding treatments. Our results indicated that incubating ELOVL5-silencing cells with AA reversed the protective effect of si-ELOVL5 on the high-glucose-mediated destruction of the MMP in cells (scale bar: 100 μm). (E) The incubation of ELOVL5-silencing cells with AA reversed the protective effect of si-ELOVL5 on high-glucose-induced changes in malondialdehyde (MDA) levels. (F) A FeRhoNox-1 fluorescence probe was used to detect the Fe^{2+} content in cells subjected to the corresponding treatments. Our results indicated that incubating ELOVL5-silencing cells with AA reversed the protective effect of si-ELOVL5 on the high-glucose-induced increases in the Fe^{2+} concentration (scale bar: 100 μm). (G) The incubation of ELOVL5-silencing cells with AA reversed the protective effect of si-ELOVL5 on the high-glucose-induced mitochondrial superoxide levels (scale bar: 100 μm). (H) A PI assay results indicating that incubating ELOVL5-silencing cells with AA reversed the protective effect of si-ELOVL5 on high-glucose-induced cell death (scale bar: 100 μm). (*P<0.05, **P<0.01, ***P<0.001, and ****P<0.0001.)

Supplementary Figure 9. ELOVL5 silencing inhibits FINO$_2$-induced ferroptosis in HGECs and HK-2 cells

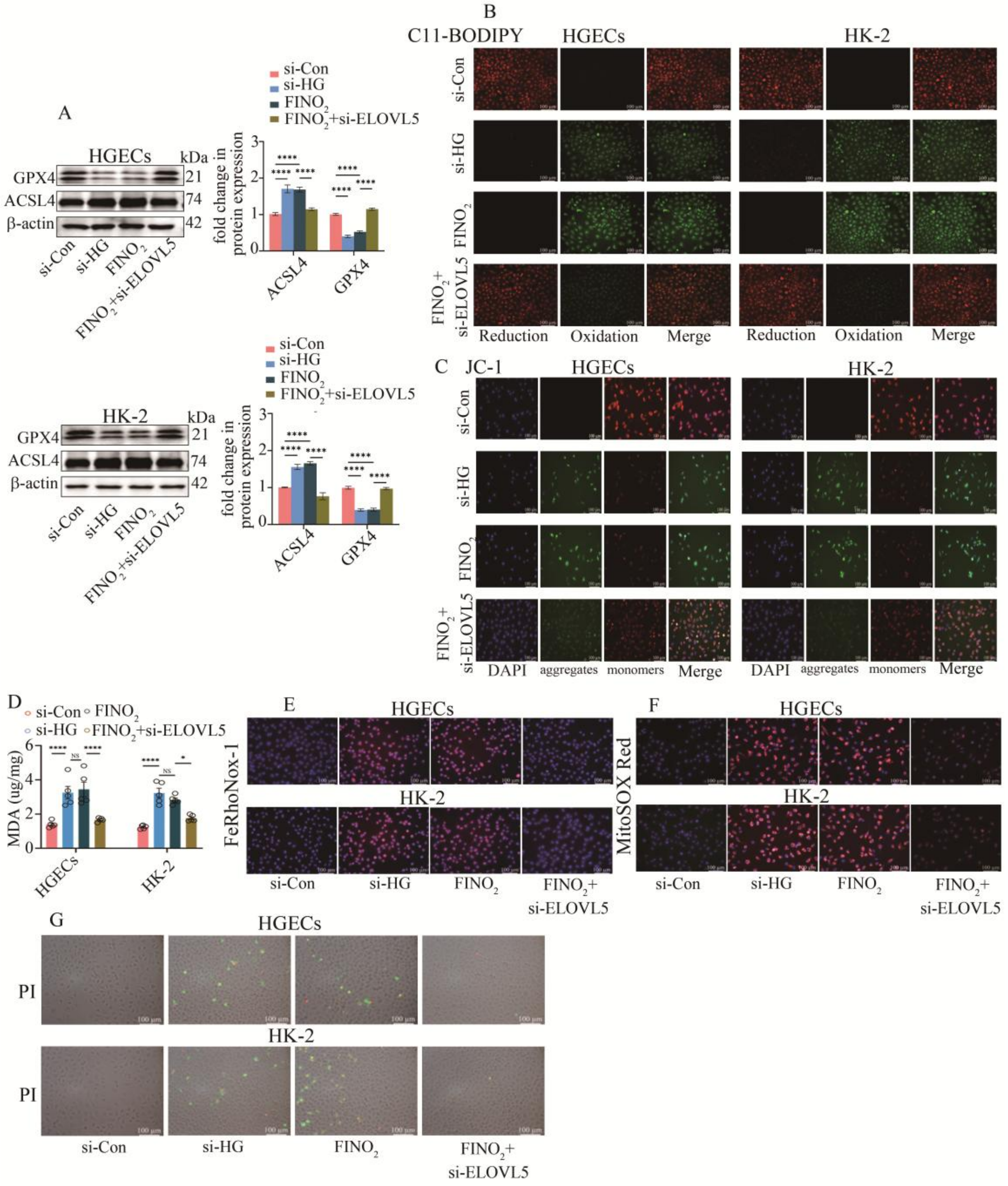

(A) Western blotting assays indicated that ELOVL5 silencing reversed the FINO$_2$-mediated increase in ACSL4 expression and decrease in GPX4 expression. (B) The C11-BODIPY 581/591 fluorescent probe was used to detect lipid peroxidation levels in HGECs and HK-2 cells. The results indicated that FINO$_2$ treatment increased lipid

peroxidation levels, which was reversed by ELOVL5 silencing in cells (scale bar: 100 μm). (C) The JC-1 fluorescent probe was used to detect changes in the mitochondrial membrane potential (MMP) in HGECs and HK-2 cells. Our results revealed that the probes used in the $FINO_2$-treated cells were mainly green fluorescent monomers. In contrast, after the intervention with si-ELOVL5, they were converted into red fluorescent polymers, indicating that ELOVL5 silencing attenuated the destruction of the MMP in $FINO_2$-treated cells (scale bar: 100 μm). (D) Malondialdehyde (MDA) levels were increased in $FINO_2$-treated cells, which was reversed by ELOVL5 silencing. (E) A FeRhoNox-1 fluorescent probe was used to detect the Fe^{2+} content in the cells. Our results revealed that FeRhoNox-1 fluorescence was elevated in $FINO_2$-treated cells but was decreased by ELOVL5 silencing. These data indicated that the Fe^{2+} content was increased in $FINO_2$-treated cells, which was reversed by ELOVL5 silencing (scale bar: 100 μm). (F) The red fluorescence intensity of MitoSOX, a mitochondria-specific superoxide indicator, was significantly increased in $FINO_2$-treated cells, which was reversed by ELOVL5 silencing (scale bar: 100 μm). (G) A PI assay indicated that the $FINO_2$ treatment increased cell death, which was reversed by ELOVL5 silencing (scale bar: 100 μm). (*P<0.05, **P<0.01, ***P<0.001, and ****P<0.0001.)

Supplementary Figure 10. Inhibition of AARS1 decreases ELOVL5 expression in DN models

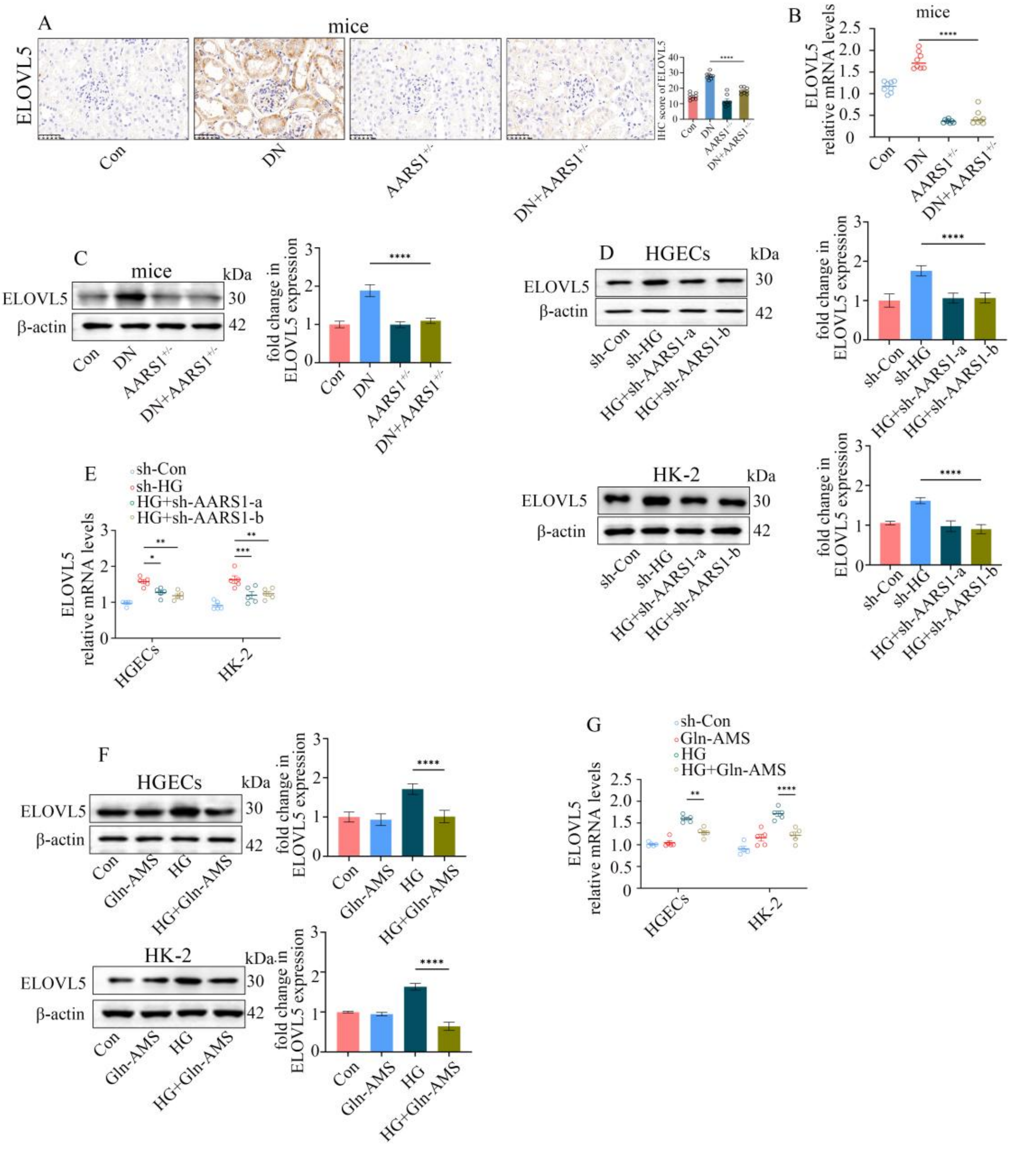

(A) IHC data indicating lower ELOVL5 levels in the kidneys of AARS1$^{+/-}$ DN model mice than in those of DN model mice (scale bar: 50 μm). (B) qPCR assays revealed that the ELOVL5 mRNA level was lower in the kidneys of AARS1$^{+/-}$ DN model mice than in those of DN mice. (C) Western blotting assays revealed that the ELOVL5 protein level was lower in the kidneys of AARS1$^{+/-}$ DN model mice than in those of

DN model mice. (D) Western blotting assays indicated that AARS1 silencing decreased ELOVL5 protein expression in hyperglycaemic cells. (E) qPCR assays indicated that AARS1 silencing decreased ELOVL5 mRNA levels in hyperglycaemic cells. (F) Western blotting assays indicated that the Gln-AMS treatment decreased ELOVL5 protein expression in hyperglycaemic cells. (G) qPCR assays indicated that the Gln-AMS treatment decreased ELOVL5 mRNA levels in hyperglycaemic cells. (*P<0.05, **P<0.01, ***P<0.001, and ****P<0.0001.)

Supplementary Figure 11. STAT1 silencing inhibits ELOVL5 expression and ferroptosis in hyperglycaemic HGECs and HK-2 cells

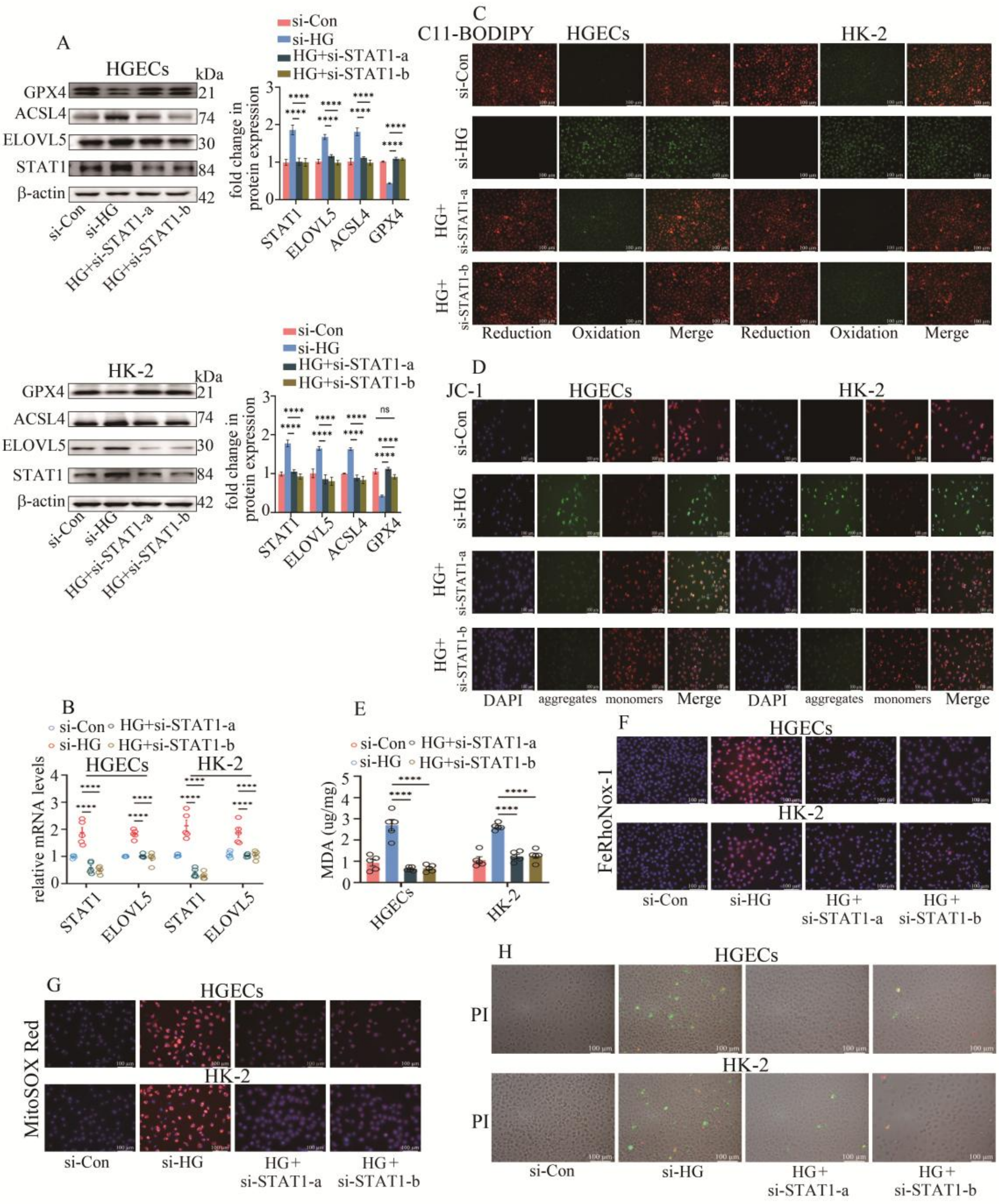

(A) Western blotting assays indicated that STAT1 silencing reversed the high-glucose-mediated increase in ELOVL5 and ACSL4 expression and decrease in GPX4 expression. (B) qPCR assays indicated that STAT1 silencing decreased ELOVL5 levels in hyperglycaemic HGECs and HK-2 cells. (C) The C11-BODIPY 581/591

fluorescent probe was used to detect lipid peroxidation levels in HGECs and HK-2 cells. The results indicated that high glucose treatment increased lipid peroxidation levels, which were reversed by STAT1 silencing in cells (scale bar: 100 μm). (D) The JC-1 fluorescent probe was used to detect changes in the mitochondrial membrane potential (MMP) in HGECs and HK-2 cells subjected to the corresponding treatments. Our results revealed that the probes in high-glucose-treated cells were mainly green fluorescent monomers. In contrast, after the intervention with si-STAT1, they were converted into red fluorescent polymers, indicating that STAT1 silencing attenuated the destruction of the MMP in hyperglycaemic cells (scale bar: 100 μm). (E) Malondialdehyde (MDA) levels were elevated in hyperglycaemic cells, which was reversed by STAT1 silencing. (F) A FeRhoNox-1 fluorescence probe was used to detect the Fe^{2+} content in cells subjected to the corresponding treatments. Our results showed that FeRhoNox-1 fluorescence was increased in hyperglycaemic cells but was decreased by STAT1 silencing. These data indicated that the Fe^{2+} content was increased in hyperglycaemic cells, which was reversed by STAT1 silencing (scale bar: 100 μm). (G) The red fluorescence intensity of MitoSOX, a mitochondria-specific superoxide indicator, was significantly increased in hyperglycaemic cells, which was reversed by STAT1 silencing (scale bar: 100 μm). (H) A PI assay indicated that the high-glucose treatment increased cell death, which was reversed by STAT1 silencing (scale bar: 100 μm). (*P<0.05, **P<0.01, ***P<0.001, and ****P<0.0001.)

Supplementary Figure 12. STAT1 modulates ELOVL5 transcription to mediate ferroptosis in hyperglycaemic cells

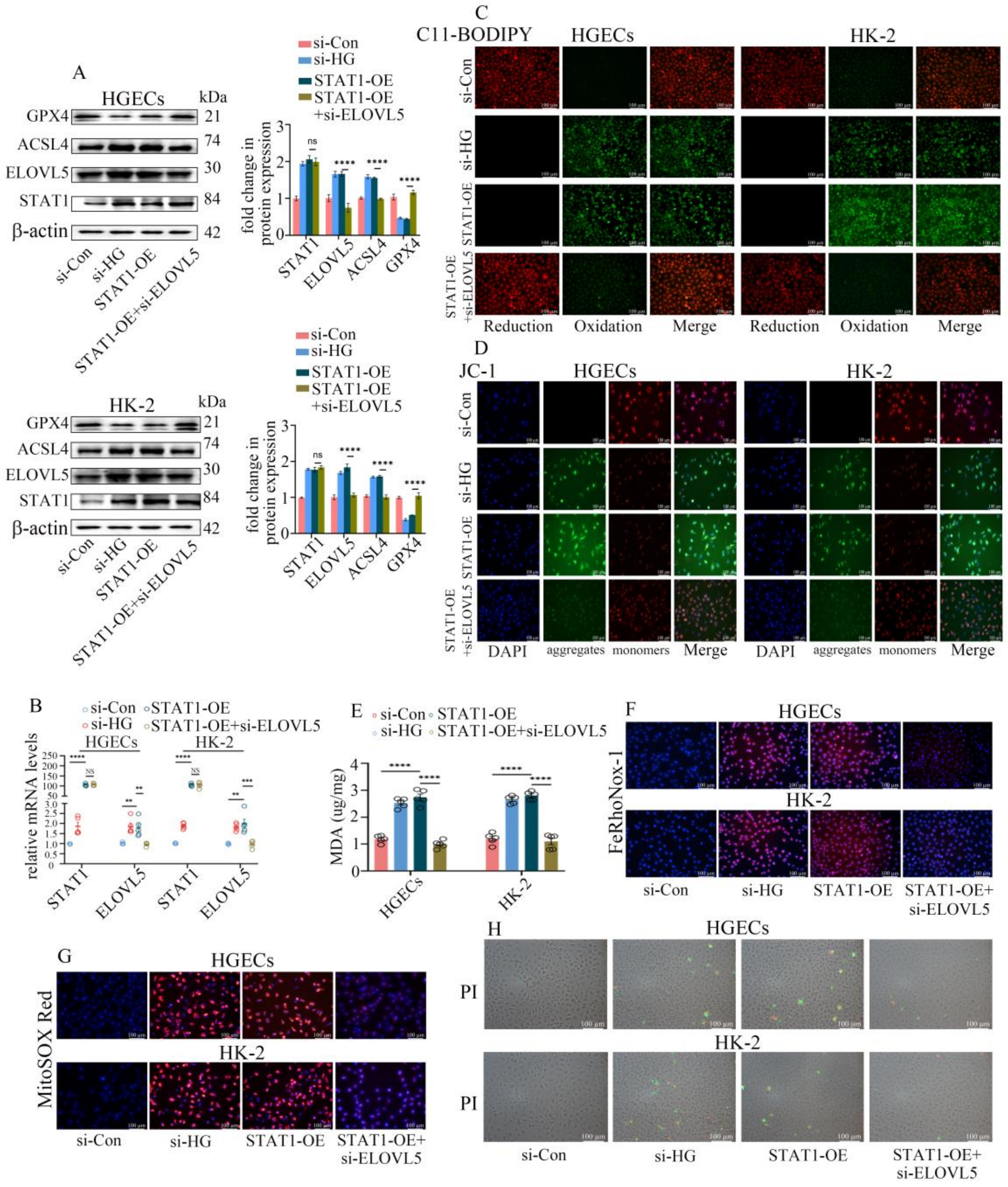

(A) Western blotting assays indicated that STAT1 overexpression increased ELOVL5 and ACSL4 expression and decreased GPX4 levels in cells. Moreover, ELOVL5 silencing reversed the STAT1 overexpression-mediated increase in ACSL4 expression and decrease in GPX4 expression. (B) Results of the qPCR analysis of STAT1 and

ELOVL5 levels in HGECs and HK-2 cells after the corresponding treatment. (C) The C11-BODIPY 581/591 fluorescent probe was used to detect lipid peroxidation levels in HGECs and HK-2 cells. The results indicated that STAT1 overexpression increased lipid peroxidation levels, which was reversed by ELOVL5 silencing (scale bar: 100 μm). (D) The JC-1 fluorescent probe was used to detect changes in the mitochondrial membrane potential (MMP) in HGECs and HK-2 cells subjected to the corresponding treatments. Our results revealed that the probes used in the STAT1-overexpressing cells were mainly green fluorescent monomers. In contrast, after the intervention with si-ELOVL5, they were converted into red fluorescent polymers, indicating that ELOVL5 silencing attenuated the destruction of the MMP in STAT1-overexpressing cells (scale bar: 100 μm). (E) Malondialdehyde (MDA) levels were increased in STAT1-overexpressing cells, which were reversed by ELOVL5 silencing. (F) A FeRhoNox-1 fluorescence probe was used to detect the Fe^{2+} content in cells subjected to the corresponding treatments. Our results revealed that FeRhoNox-1 fluorescence was increased in STAT1-overexpressing cells but was decreased by ELOVL5 silencing. These data indicated that the Fe^{2+} content was increased in STAT1-overexpressing cells, which was reversed by ELOVL5 silencing (scale bar: 100 μm). (G) The red fluorescence intensity of MitoSOX, a mitochondria-specific superoxide indicator, was significantly increased in STAT1-overexpressing cells, which was reversed by ELOVL5 silencing (scale bar: 100 μm). (H) PI staining indicated that STAT1 overexpression increased the death of HGECs and HK-2 cells, which was reversed by ELOVL5 silencing (scale bar: 100 μm). (*P<0.05, **P<0.01, ***P<0.001,

and ****P<0.0001.)

Supplementary Figure 13. STAT1 modulates ELOVL5 transcription in HGECs and HK-2 cells

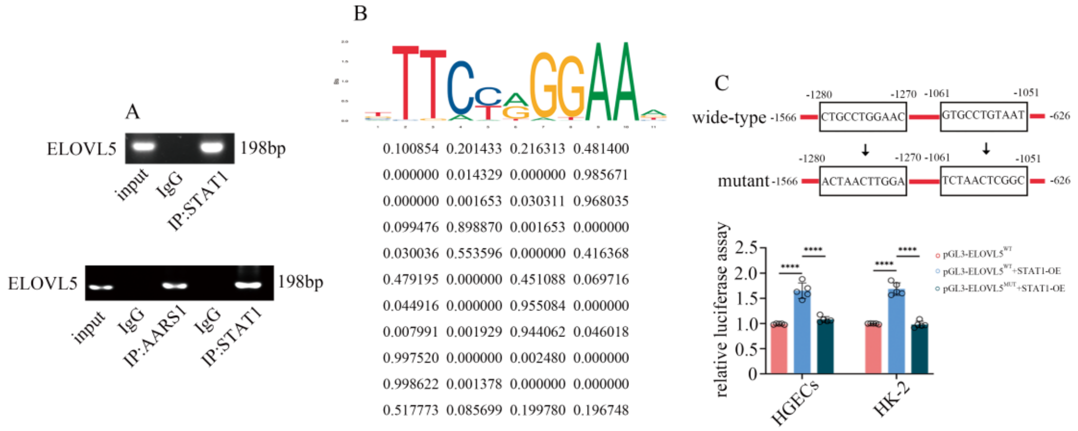

(A) ChIP assays indicated that STAT1 and AARS1 was enriched at the same ELOVL5 promoter region. (B) The predicted STAT1 binding site in the ELOVL5 promoter region. (C) The activity of the ELOVL5 promoter was detected after the corresponding treatments. (*P<0.05, **P<0.01, ***P<0.001, and ****P<0.0001.)

Supplementary Figure 14. The STAT1 inhibitor fludarabine (Flu) inhibits ferroptosis in hyperglycaemic cells

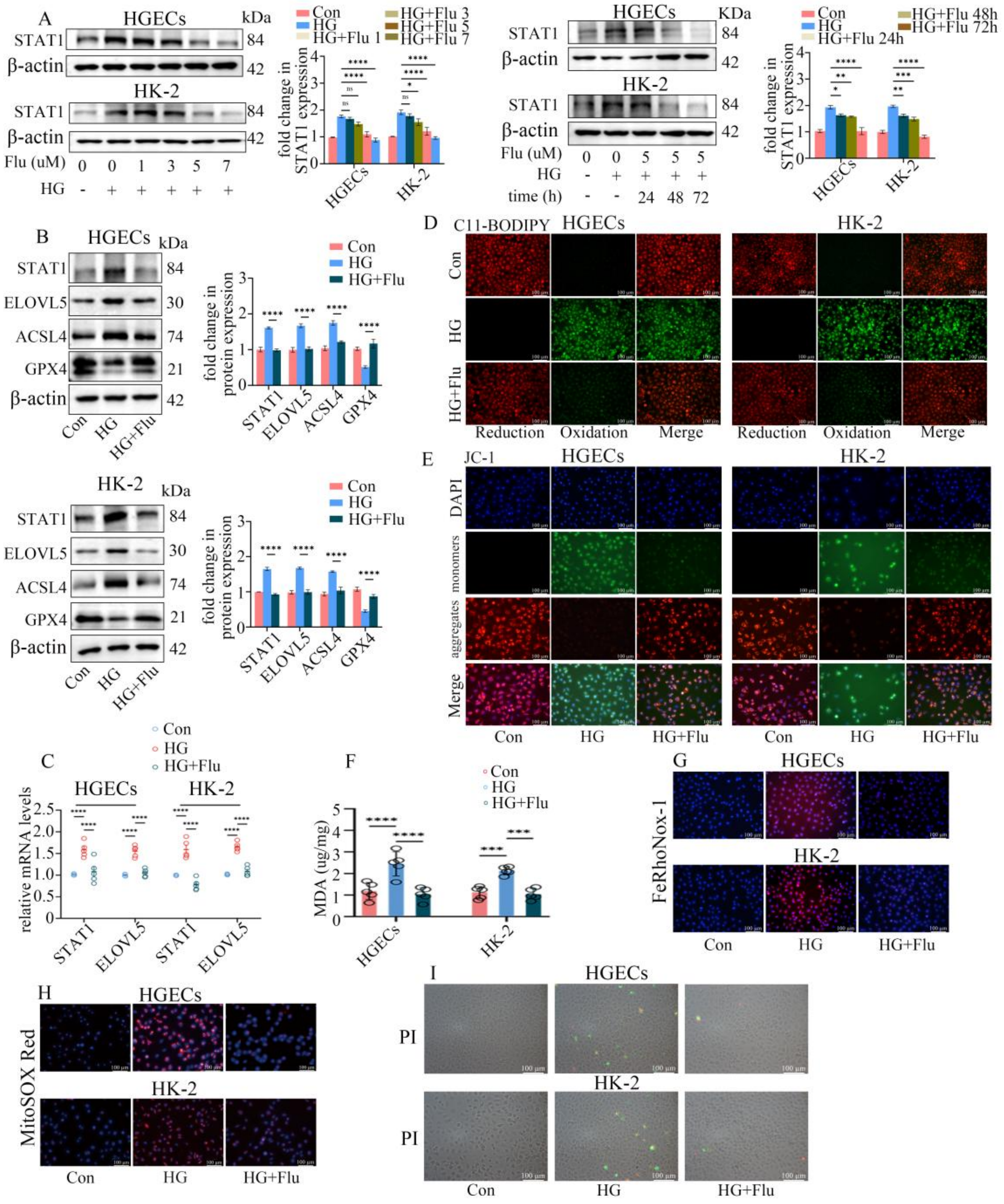

(A) Western blotting assays indicated that Flu treatment inhibited STAT1 expression in a concentration- and time-dependent manner in hyperglycaemic cells. Incubation of cells with 5 μM Flu for 72 hours caused a significant reversal of high-glucose-induced STAT1 expression. (B) Western blotting assays indicated that Flu treatment reversed

the high-glucose-mediated increase in STAT1, ELOVL5 and ACSL4 expression and decrease in GPX4 expression. (C) qPCR assays indicated that Flu treatment decreased STAT1 and ELOVL5 levels in HGECs and HK-2 cells. (D) The C11-BODIPY 581/591 fluorescent probe was used to detect lipid peroxidation levels in HGECs and HK-2 cells. The results indicated that high glucose concentrations increased lipid peroxidation levels in cells, which were reversed by Flu treatment (scale bar: 100 μm). (E) The JC-1 fluorescence probe was used to detect changes in the mitochondrial membrane potential (MMP) in HGECs and HK-2 cells. Our results showed that the probes in high-glucose-treated cells were mainly in the form of green fluorescent monomers. In contrast, after Flu treatment, they were converted into red fluorescent polymers, indicating that Flu treatment attenuated the destruction of the MMP in hyperglycaemic cells (scale bar: 100 μm). (F) Malondialdehyde (MDA) levels were elevated in hyperglycaemic cells, which were reversed by Flu treatment. (G) A FeRhoNox-1 fluorescence probe was used to detect the Fe^{2+} content in the cells. Our results revealed that FeRhoNox-1 fluorescence was increased in hyperglycaemic cells but was decreased via Flu treatment. These data indicated that the Fe^{2+} content was increased in hyperglycaemic cells, which was reversed by Flu treatment (scale bar: 100 μm). (H) The red fluorescence intensity of MitoSOX, a mitochondria-specific superoxide indicator, was significantly increased in hyperglycaemic cells, which was reversed by Flu treatment (scale bar: 100 μm). (I) A PI assay indicated that high-glucose treatment increased cell death, which was reversed by Flu treatment (scale bar: 100 μm). (*P<0.05, **P<0.01, ***P<0.001, and ****P<0.0001.)

Supplementary Figure 15. AARS1 modulates lactylation to trigger ferroptosis in cells

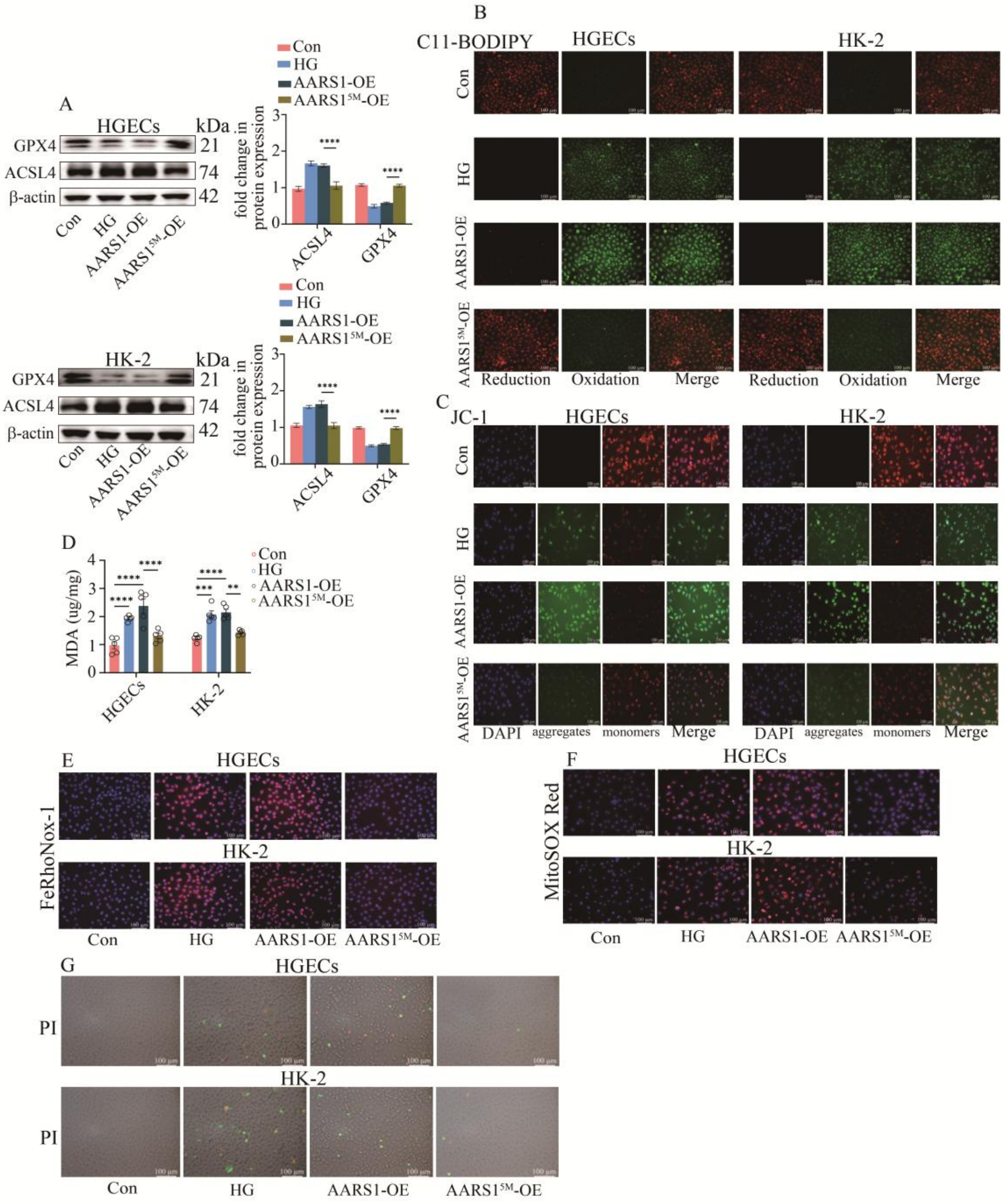

(A) Western blotting assays indicated that AARS1 overexpression increased ACSL4 expression but decreased GPX4 levels in cells. However, $AARS1^{5M}$ did not have these effects. (B) The C11-BODIPY 581/591 fluorescent probe was used to detect lipid peroxidation levels in HGECs and HK-2 cells. These results indicated that AARS1

overexpression increased lipid peroxidation levels. However, AARS1^{5M} did not have these effects (scale bar: 100 μm). (C) The JC-1 fluorescent probe was used to detect changes in the mitochondrial membrane potential (MMP) in HGECs and HK-2 cells. Our results revealed that the probes in the AARS1-overexpressing group were mainly green fluorescent monomers. However, AARS1^{5M} did not alter the MMP (scale bar: 100 μm). (D) Malondialdehyde (MDA) levels were elevated in AARS1-overexpressing cells. However, AARS1^{5M} did not have these effects. (E) A FeRhoNox-1 fluorescent probe was used to detect the Fe^{2+} content in the cells. Our results revealed that FeRhoNox-1 fluorescence was increased in AARS1-overexpressing cells. However, AARS1^{5M} did not have these effects (scale bar: 100 μm). (F) The red fluorescence intensity of MitoSOX, a mitochondria-specific superoxide indicator, was significantly increased in AARS1-overexpressing cells. However, AARS1^{5M} did not have these effects (scale bar: 100 μm). (G) A PI assay results indicating that AARS1 overexpression increased cell death. However, AARS1^{5M} did not have these effects (scale bar: 100 μm). (*$P<0.05$, **$P<0.01$, ***$P<0.001$, and ****$P<0.0001$.)

Supplementary Figure 16. AARS1 modulates STAT1^{K193} lactylation to increase STAT1 transcriptional activity

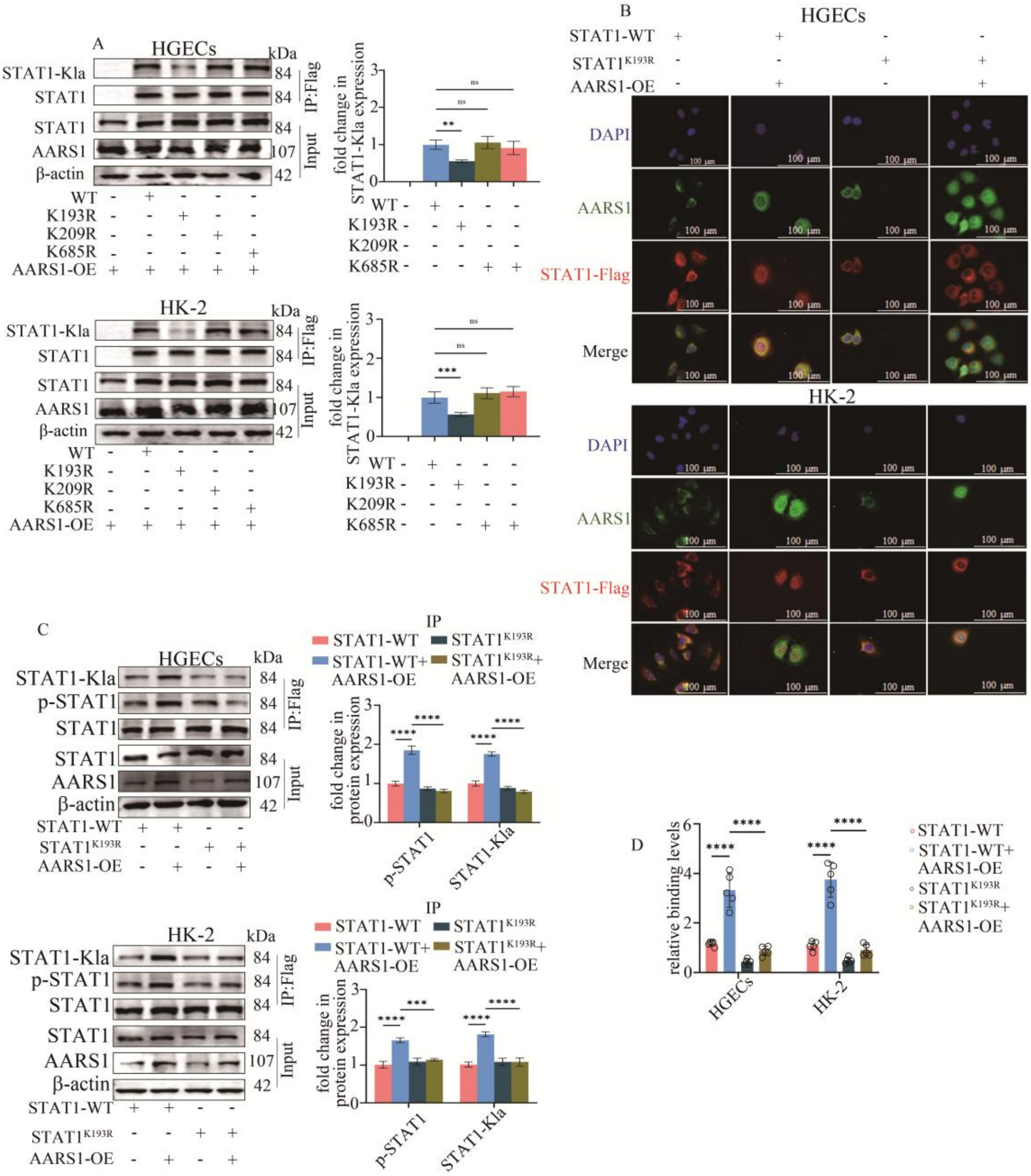

(A) By constructing STAT1 lactylation site mutant plasmids, the STAT1^{K193} site was identified as the most important lactylation site regulated by AARS1. (B) Immunofluorescence staining indicating that AARS1 overexpression promoted STAT1 nuclear translocation in HGECs and HK-2 cells. Additionally, mutation of the

STAT1^{K193} site weakened STAT1 nuclear translocation induced by AARS1 overexpression (scale bar: 100 μm). (C) Western blotting indicated that AARS1 overexpression increased STAT1 phosphorylation in HGECs and HK-2 cells. Additionally, mutation of the STAT1^{K193} site weakened STAT1 phosphorylation induced by AARS1 overexpression. (D) STAT1-Flag plasmid, STAT1^{K193R}-Flag plasmid, and AARS1-OE plasmid was transfected in STAT1-sliencing cells accordingly. ChIP-qPCR revealed that AARS1 overexpression enhanced the binding of STAT1 to the ELOVL5 promoter region in HGECs and HK-2 cells. However, when the STAT1^{K193} site was mutated, the effect of AARS1 overexpression was weakened. (*$P<0.05$, **$P<0.01$, ***$P<0.001$, and ****$P<0.0001$.)

Supplementary Figure 17. β-alanine inhibited AARS1-induced H3K18 and STAT1 lactylation

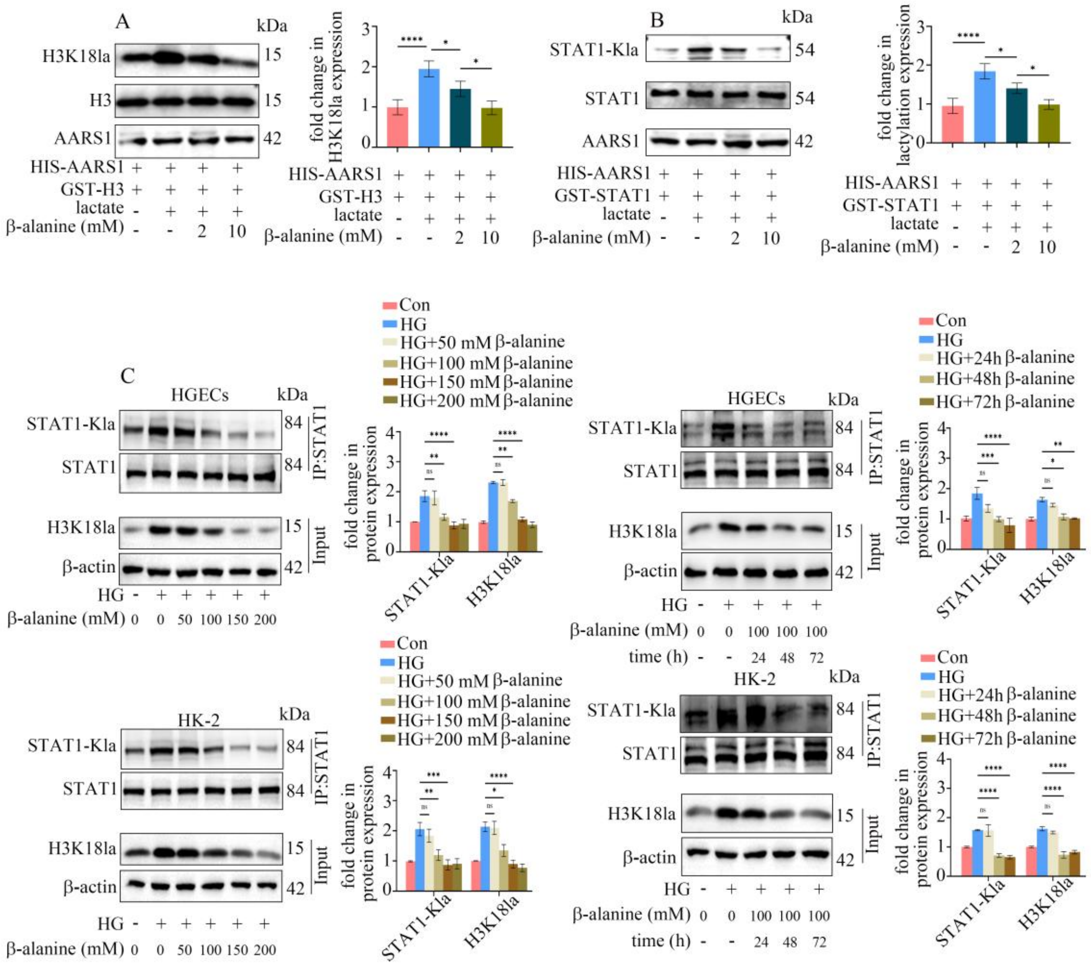

(A) β-alanine inhibited AARS1-induced H3K18la *in vitro*. (B) β-alanine inhibited AARS1-induced STAT1 lactylation *in vitro*. (C) β-alanine inhibited high-glucose-induced STAT1 and H3K18 lactylation in a concentration- and time-dependent manner in hyperglycaemic cells. Incubation of cells with 100 mM β-alanine for 72 hours caused a significant reversal of high-glucose-induced H3K18 and STAT1 lactylation. (*P<0.05, **P<0.01, ***P<0.001, and ****P<0.0001.)

Supplementary Figure 18. β-alanine mitigates ferroptosis in hyperglycaemic cells by inhibiting AARS1-induced lactylation

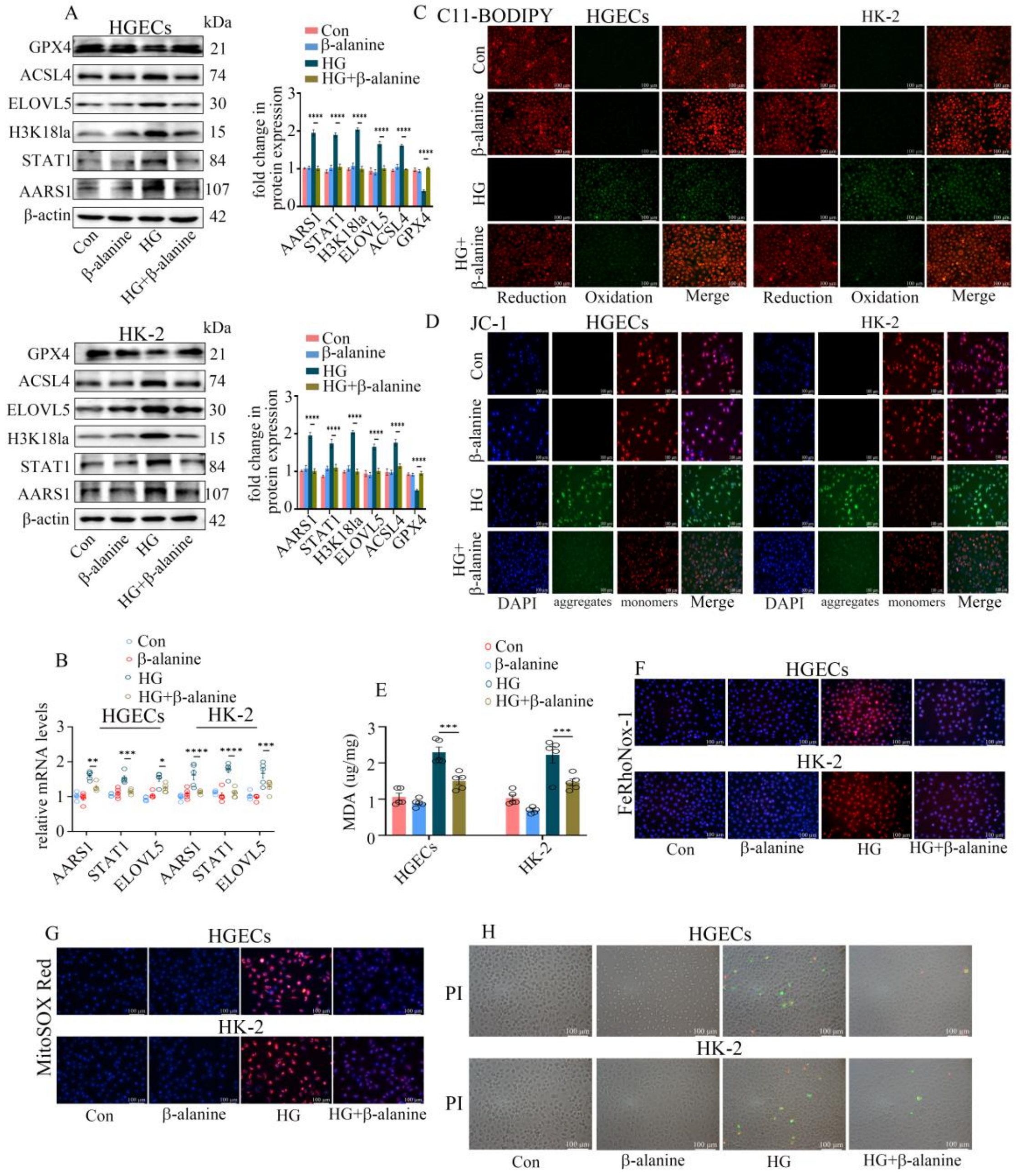

(A) Western blotting assays revealed that β-alanine reversed the high-glucose-mediated increase in AARS1, H3K18la, STAT1, ELOVL5 and ACSL4 levels and decrease in GPX4 expression. (B) qPCR assays indicated that β-alanine decreased AARS1, STAT1 and ELOVL5 mRNA levels in hyperglycaemic HGECs and HK-2

cells. (C) The C11-BODIPY 581/591 fluorescent probe was used to detect lipid peroxidation levels in HGECs and HK-2 cells. The results indicated that high glucose concentrations increased lipid peroxidation, which was reversed by β-alanine treatment in cells (scale bar: 100 μm). (D) The JC-1 fluorescent probe was used to detect changes in the mitochondrial membrane potential (MMP) in HGECs and HK-2 cells. Our results revealed that the probes in high-glucose-treated cells were mainly in the form of green fluorescent monomers. In contrast, after β-alanine treatment, they were converted into red fluorescent polymers, indicating that β-alanine treatment attenuated the destruction of the MMP in hyperglycaemic cells (scale bar: 100 μm). (E) Malondialdehyde (MDA) levels were elevated in hyperglycaemic cells, which were reversed via β-alanine treatment. (F) A FeRhoNox-1 fluorescent probe was used to detect the Fe^{2+} content in cells subjected to the corresponding treatments. Our results showed that FeRhoNox-1 fluorescence was increased in hyperglycaemic cells but was decreased by the β-alanine treatment. These data indicated that the Fe^{2+} content was increased in hyperglycaemic cells, which was reversed by the β-alanine treatment (scale bar: 100 μm). (G) The red fluorescence intensity of MitoSOX, a mitochondria-specific superoxide indicator, was significantly increased in hyperglycaemic cells, which was reversed by β-alanine treatment (scale bar: 100 μm). (H) A PI assay indicated that high-glucose treatment increased cell death, which was reversed by β-alanine treatment (scale bar: 100 μm). (*P<0.05, **P<0.01, ***P<0.001, and ****P<0.0001.)
