# Supplementary material for: AARS1-mediated lactylation of H3K18 and STAT1 promotes ferroptosis in diabetic nephropathy
Source: Cell Death Differ. 2025 Sep 23;33(3):589–604. doi: 10.1038/s41418-025-01587-4 (PMC13036035; doi:10.1038/s41418-025-01587-4)
Supplement: Supplementary file 2 — supplemental table 1 [file 41418_2025_1587_MOESM2_ESM.doc]

**Supplemental Table 1.** Characteristics of participants diagnosed with diabetic nephropathy

| **gender** | **Age（Year）** | **BMI (kg/m2)** | **SBP**  **(mmHg)** | **DBP (mmHg)** | **HbA1C (%)** | **FBG (mmol/l)** | **CREA**  **(umol/l)** | **ALB**  **(g/L)** | **CCr（ml/min）** | **24hUPQ（mg）** | **TP**  **(g/L)** | **eGFR**  **(ml/min/)** |
| --- | --- | --- | --- | --- | --- | --- | --- | --- | --- | --- | --- | --- |
| **DN stage 2 （n=10）** | | | | | | | | | | | | |
| Male/Female  2/8 | 55.4±10.4 | 25.2±2.2 | 151.3±24.3 | 89.3±20.2 | 6.7±0.8 | 6.2±1.0 | 95.7±14.0 | 53.7±11.8 | 77.5±6.0 | 3625.4±987.1 | 57.0±6.4 | 72.4±6.8 |
| **DN stage 3 （n=14）** | | | | | | | | | | | | |
| Male/Female  3/11 | 53.6±6.5 | 26.0±3.8 | 153.7±16.0 | 81.0±12.0 | 7.0±1.0 | 5.7±0.4 | 151.6±30.9 | 39.6±12.4 | 50.2±10.1 | 4782.7±928.3 | 59.1±9.9 | 43.9±9.2 |
| **DN stage 4 （n=12）** | | | | | | | | | | | | |
| Male/Female  5/7 | 60.6±11.0 | 23.1±2.3 | 155.4±20.0 | 78.0±11.2 | 8.0±1.0 | 7.5±3.4 | 272.3±29.6 | 27.4±5.3 | 19.7±5.7 | 5147.1±804.8 | 55.4±6.2 | 16.8±5.6 |

Data are presented as means ± SD. BMI (Body Mass Index), SBP (systolic blood pressure), DBP (diastolic blood pressure), HbA1c (glycated hemoglobin), FBG (fasting blood glucose), CREA (creatinine), ALB (albumin), CCr (Creatinine Clearance), 24hUPQ (24-hour urinary protein quantity), TP (Total Protein), eGFR (Estimated Glomerular Filtration Rate).
