# Supplementary material for: AARS1-mediated lactylation of H3K18 and STAT1 promotes ferroptosis in diabetic nephropathy
Source: Cell Death Differ. 2025 Sep 23;33(3):589–604. doi: 10.1038/s41418-025-01587-4 (PMC13036035; doi:10.1038/s41418-025-01587-4)
Supplement: Supplementary file 3 — supplemental table 2 [file 41418_2025_1587_MOESM3_ESM.docx]

| **Supplemental Table 2. serum biochemical indices of the mice** | | | | |
| --- | --- | --- | --- | --- |
|  | Con | AARS1+/- | DN | DN+AARS1+/- |
| FBG(mmol/L) | 4.3625±1.1612 | 4.725±2.4778 | 21.5875±1.1281 ^####^ | 20.7125±1.3974 |
| TG(mmol/L) | 0.9713±0.1049 | 0.9288±0.0937 | 1.3295±0.1548 ^###^ | 1.3175±0.1402 |
| TC(mmol/L) | 3.565±0.6559 | 3.1725±0.0573 | 3.8225±1.0818 ^#^ | 3.3363±0.8451**^**^ |
| BUN(mmol/L) | 1.4425±1.7739 | 1.1625±0.4335 | 1.8925±0.5519 ^##^ | 1.4675±0.3413** |
| HDL(mmol/L) | 0.1875±0.0818 | 0.1775±0.0745 | 0.0988±0.0306 ^####^ | 0.1475±0.0257* |
| LDL(mmol/L) | 0.1425±0.0336 | 0.1565±0.0291 | 0.2512±0.0188 ^####^ | 0.2113±0.0306** |
| MDA(nmol/ml) | 2.6322±0.1004 | 2.4859±0.1224 | 3.2083±0.1863 ^##^ | 2.6135±0.2083** |
| Scr(umol/L) | 39.8214±7.9623 | 38.6251±7.6253 | 50.6251±9.5253 ^##^ | 43.2251±8.1223**^*^ |
|  |  |  |  |  |
|  | DN+Fer-1 | DN+Flu | β-alanine | DN+β-alanine |
| FBG(mmol/L) | 20.1275±1.6482 | 20.5875±1.7281 | 4.2532±2.4231 | 20.7875±0.8593 |
| TG(mmol/L) | 1.3525±0.1341 | 1.3295±0.1508 | 0.7513±0.0974 | 1.3575±0.0981 |
| TC(mmol/L) | 3.26±0.9917*** | 3.5225±0.6818** | 3.345±1.853 | 3.3108±0.419** |
| BUN(mmol/L) | 1.6425±0.8897* | 1.4925±0.559** | 1.3903±0.9352 | 1.2147±0.5242**** |
| HDL(mmol/L) | 0.1363±0.0746** | 0.1288±0.0506*** | 0.1271±0.0292 | 0.1613±0.1219**** |
| LDL(mmol/L) | 0.2163±0.01531* | 0.2282±0.0168** | 0.1223±0.0191 | 0.16375±0.0313**** |
| MDA(nmol/ml) | 2.56±0.2118*** | 2.5083±0.1563*** | 2.5928±0.1614 | 2.6328±0.1503** |
| Scr(umol/L) | 40.4023±8.2734** | 41.3654±8.4204** | 36.1267±7.3156 | 42.2625±8.9721** |

FBG (Fasting Blood Glucose). TG (Triglyceride). TC (Total Cholesterol). BUN (Blood Urea Nitrogen). HDL (High-Density Lipoprotein). LDL (Low-Density Lipoprotein). MDA (Malondialdehyde). Scr (Serum reatinine). Quantitated data were means±SD. Statistical significance was assessed by unpaired t-test (P values adjusted for 5 comparisons); ^##^ P<0.01, ^###^ p<0.001, ^####^ p<0.0001 compared with the Con mouse groups. * p<0.05, ** p<0.01, *** p<0.001, **** p<0.0001 compared with the DN mouse group.
