# Supplementary material for: AARS1-mediated lactylation of H3K18 and STAT1 promotes ferroptosis in diabetic nephropathy
Source: Cell Death Differ. 2025 Sep 23;33(3):589–604. doi: 10.1038/s41418-025-01587-4 (PMC13036035; doi:10.1038/s41418-025-01587-4)
Supplement: Supplementary file 8 — supplemental table 7 [file 41418_2025_1587_MOESM8_ESM.docx]

**Supplemental Table 7.** Putative binding sites for STAT1 in the ELOVL5 promoter region

| Matrix ID | Gene name | Start | End | Predicted sequence |
| --- | --- | --- | --- | --- |
| [**MA0137.3**](https://jaspar.genereg.net/matrix/MA0137.3) | MA0137.3.**STAT1** | -1270 | -1280 | CTGCCTGGAAC |
| [**MA0137.3**](https://jaspar.genereg.net/matrix/MA0137.3) | MA0137.3.**STAT1** | -1051 | -1061 | GTGCCTGTAAT |
