# Supplementary material for: AARS1-mediated lactylation of H3K18 and STAT1 promotes ferroptosis in diabetic nephropathy
Source: Cell Death Differ. 2025 Sep 23;33(3):589–604. doi: 10.1038/s41418-025-01587-4 (PMC13036035; doi:10.1038/s41418-025-01587-4)
Supplement: Supplementary file 9 — supplemental table 8 [file 41418_2025_1587_MOESM9_ESM.docx]

**Supplemental Table 8.** The information of used antibodies

| Antibody name | Cat No. | Information |
| --- | --- | --- |
| AARS1 Polyclonal antibody | 17394-1-AP | ProteinTech, Wuhan, China |
| STAT1 Polyclonal antibody | 10144-2-AP | ProteinTech, Wuhan, China |
| Anti-ELOVL5  Monoclonal Anti-4 HNE | ab205535  HNE13-M | Abcam, Cambridge, UK  Alpha Diagnostic Intl. Inc, Texas, USA |
| Anti-L-Lactyl-Histone H3 (Lys18) Rabbit mAb | PTM-1406RM | PTM-BIO, HangZhou, China |
| Anti-L-Lactyl Lysine Rabbit pAb (pan-antibody of lactylation) | PTM-1401RM | PTM-BIO, HangZhou, China |
| β-actin Recombinant antibody | 81115-1-RR | ProteinTech, Wuhan, China |
| CD31 Polyclonal antibody | 11265-1-AP | ProteinTech, Wuhan, China |
| Anti-STAT1 (phospho S727) | ab109461 | Abcam, Cambridge, UK |
| Histone-H3 Polyclonal antibody | 17168-1-AP | ProteinTech, Wuhan, China |
| GST Tag Polyclonal antibody | 10000-0-AP | ProteinTech, Wuhan, China |
| 6*his, His tag Polyclonal antibody | 10001-0-AP | ProteinTech, Wuhan, China |
| ACSL4/FACL4 Polyclonal antibody | 22401-1-AP | ProteinTech, Wuhan, China |
| GPX4 Polyclonal antibody | 30388-1-AP | ProteinTech, Wuhan, China |
| Anti-Malondialdehyde antibody | ab27642 | Abcam, Cambridge, UK |
| HA tag Polyclonal antibody | 51064-2-AP | ProteinTech, Wuhan, China |
| DYKDDDDK tag Polyclonal antibody (Binds to FLAG® tag epitope) | 20543-1-AP | ProteinTech, Wuhan, China |
