# Supplementary material for: AARS1-mediated lactylation of H3K18 and STAT1 promotes ferroptosis in diabetic nephropathy
Source: Cell Death Differ. 2025 Sep 23;33(3):589–604. doi: 10.1038/s41418-025-01587-4 (PMC13036035; doi:10.1038/s41418-025-01587-4)
Supplement: Supplementary file 10 — supplemental table 9 [file 41418_2025_1587_MOESM10_ESM.docx]

| **Supplemental Table 9. primers used for the real-time RT-PCR analysis** | |  |
| --- | --- | --- |
| Human-AARS1-F | GTGAAGGTGGATGACAGCAGTG | |
| Human-AARS1-R | CCACTTTCAGGTCACCGTAGATG | |
| Human-STAT1-F： | CAGCTTGACTCAAAATTCCTGGA | |
| Human-STAT1-R： | TGAAGATTACGCTTGCTTTTCCT | |
| Human-ELOVL5-F | CAAGAACAACCACCAGATCACG | |
| Human-ELOVL5-R | CCAGTTCATCACAAACCACCAG | |
| Human-beta-actin-F： | CGGCTACAGCTTCACCACCAC | |
| Human-beta-actin-R： | GCCATCTCTTGCTCGAAGTCCAG | |
|  |  | |
| Mouse-AARS1-F | TACTTATGGATTCCCAGTGGACC | |
| Mouse-AARS1-R | TCCTCAAAGCCATTCATATCTACC | |
| Mouse-STAT1-F | GAGTTCCGACACCTGCAACTG | |
| Mouse-STAT1-R | TCTTCGGTGACAATGAGAGGC | |
| Mouse-ELOVL5-F | TCCAAACTCATCGAATTCATGG | |
| Mouse-ELOVL5-R | CAGATGTTGAGCATGGTAGCG | |
| Mouse-beta-actin-F | CATTGCTGACAGGATGCAGAAGG | |
| Mouse-beta-actin-R | TGCTGGAAGGTGGACAGTGAGG | |
