# Supplementary material for: AARS1-mediated lactylation of H3K18 and STAT1 promotes ferroptosis in diabetic nephropathy
Source: Cell Death Differ. 2025 Sep 23;33(3):589–604. doi: 10.1038/s41418-025-01587-4 (PMC13036035; doi:10.1038/s41418-025-01587-4)
Supplement: Supplementary file 11 — supplemental table 10 [file 41418_2025_1587_MOESM11_ESM.docx]

| Primer pairs | Sequences |
| --- | --- |
| 1  2  3  4 | F 5’-GAGAACTCTAAGTCTCTGGC-3’  R 5’-TCAATAAATACGTGTTAGAT-3’  F 5’- AATTTTGAATACCTAACATA -3’  R 5’- TATTTACAATGTAATGCTTA -3’  F 5’- CACTTTGGCAAGCTGAGGCG-3’  R 5’- GATCTCAGCTCACTGCAACC -3’  F 5’- TCAGCTTTAACGATGAAGTG-3’  R 5’- TCTTCCTAATAAGCCTGCGT -3’ |

**Supplemental Table 10.** Primers used for ChIP assay in ELOVL5 promoter region
