# Supplementary material for: AARS1-mediated lactylation of H3K18 and STAT1 promotes ferroptosis in diabetic nephropathy
Source: Cell Death Differ. 2025 Sep 23;33(3):589–604. doi: 10.1038/s41418-025-01587-4 (PMC13036035; doi:10.1038/s41418-025-01587-4)

Figure 1

C

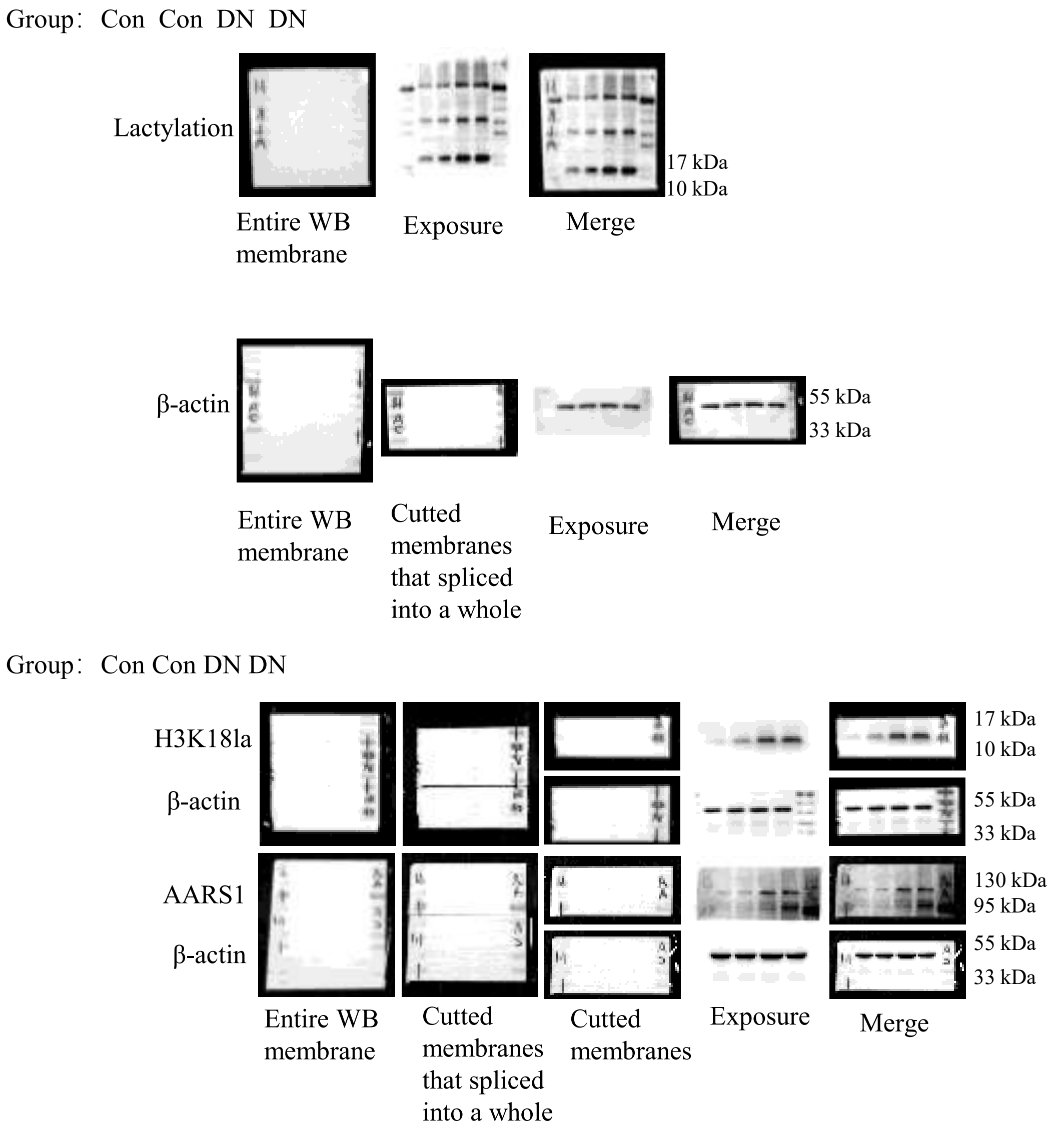
Group: Con Con DN DN
Lactylation
17 kDa
10 kDa
Entire WB membrane
Exposure
Merge
β-actin
55 kDa
33 kDa
Entire WB membrane
Cutted membranes that spliced into a whole
Exposure
Merge
Group: Con Con DN DN
H3K18la
β-actin
AARS1
β-actin
17 kDa
10 kDa
55 kDa
33 kDa
130 kDa
95 kDa
55 kDa
33 kDa
Entire WB membrane
Cutted membranes that spliced into a whole
Cutted membranes
Exposure
Merge

Figure 2

B

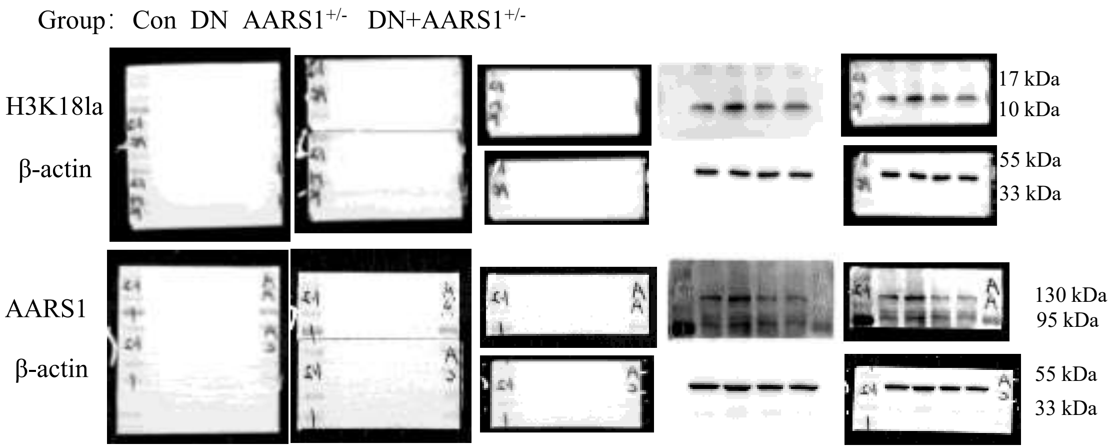
Group: Con DN AARS1+/- DN+AARS1+/-
H3K18la
β-actin
AARS1
β-actin
17 kDa
10 kDa
55 kDa
33 kDa
130 kDa
95 kDa
55 kDa
33 kDa

D

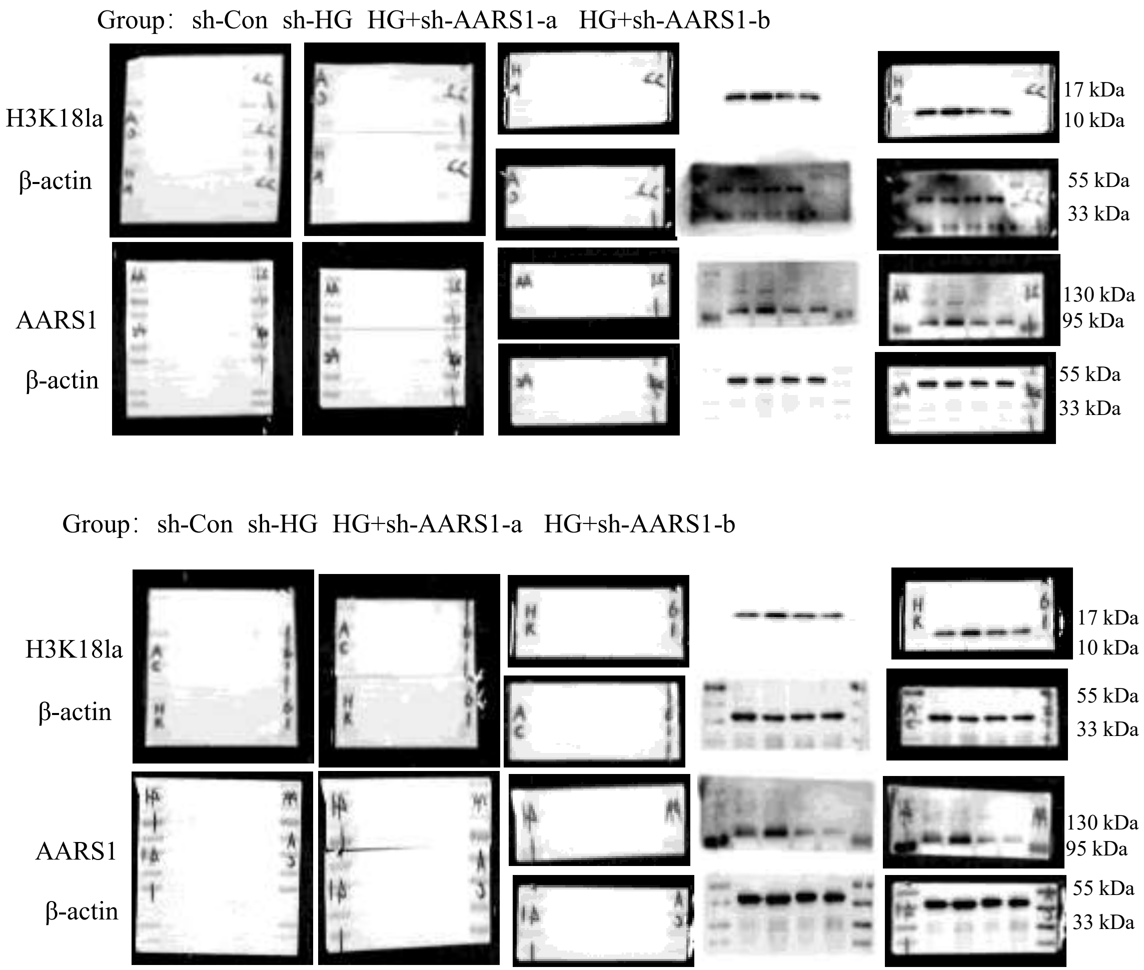
Group: sh-Con sh-HG HG+sh-AARS1-a HG+sh-AARS1-b
H3K18la
β-actin
AARS1
β-actin
17 kDa
10 kDa
55 kDa
33 kDa
130 kDa
95 kDa
55 kDa
33 kDa
Group: sh-Con sh-HG HG+sh-AARS1-a HG+sh-AARS1-b
H3K18la
β-actin
AARS1
β-actin
17 kDa
10 kDa
55 kDa
33 kDa
130 kDa
95 kDa
55 kDa
33 kDa

Figure 3

D

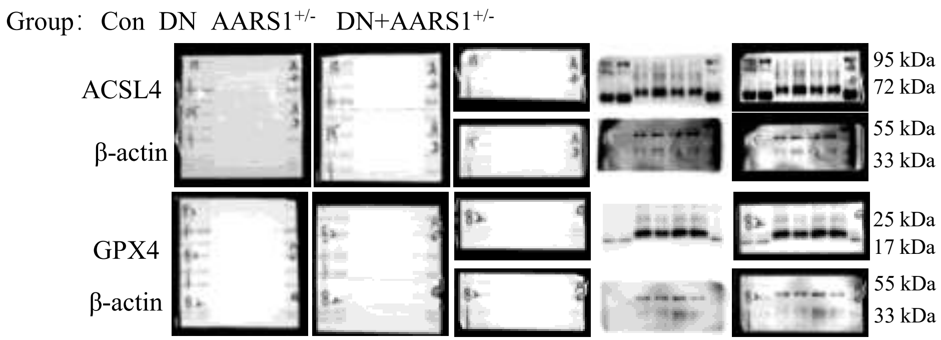
Group：Con DN AARS1+/- DN+AARS1+/-
ACSL4
β-actin
GPX4
β-actin
95 kDa
72 kDa
55 kDa
33 kDa
25 kDa
17 kDa
55 kDa
33 kDa

Figure 4

A

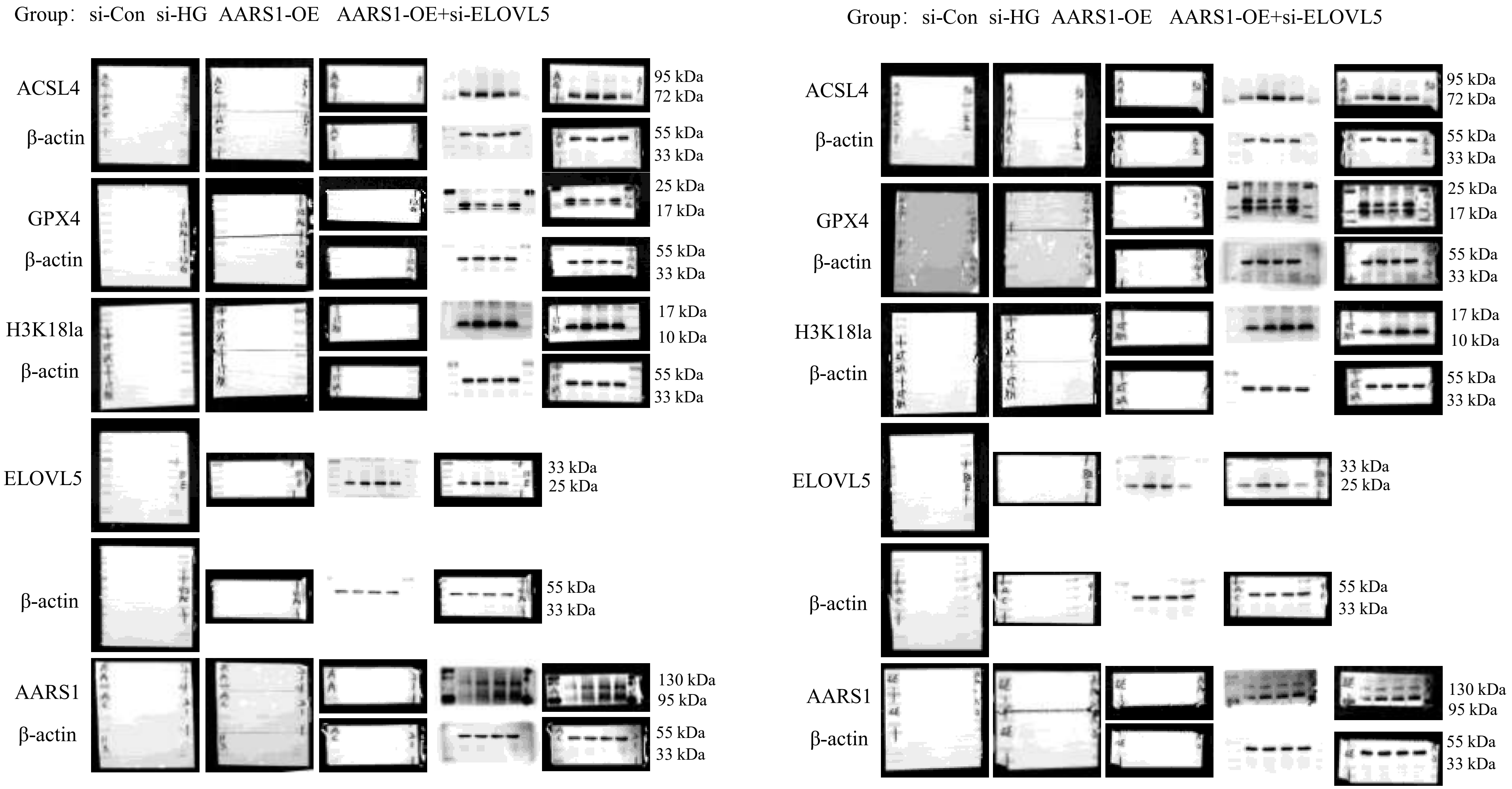
Group: si-Con si-HG AARS1-OE AARS1-OE+si-ELOVL5
ACSL4
β-actin
GPX4
β-actin
H3K18la
β-actin
ELOVL5
β-actin
AARS1
β-actin
95 kDa
72 kDa
55 kDa
33 kDa
25 kDa
17 kDa
55 kDa
33 kDa
17 kDa
10 kDa
55 kDa
33 kDa
33 kDa
25 kDa
55 kDa
33 kDa
130 kDa
95 kDa
55 kDa
33 kDa
Group: si-Con si-HG AARS1-OE AARS1-OE+si-ELOVL5
ACSL4
β-actin
GPX4
β-actin
H3K18la
β-actin
ELOVL5
β-actin
AARS1
β-actin
95 kDa
72 kDa
55 kDa
33 kDa
25 kDa
17 kDa
55 kDa
33 kDa
17 kDa
10 kDa
55 kDa
33 kDa
33 kDa
25 kDa
55 kDa
33 kDa
130 kDa
95 kDa
55 kDa
33 kDa

Figure 5

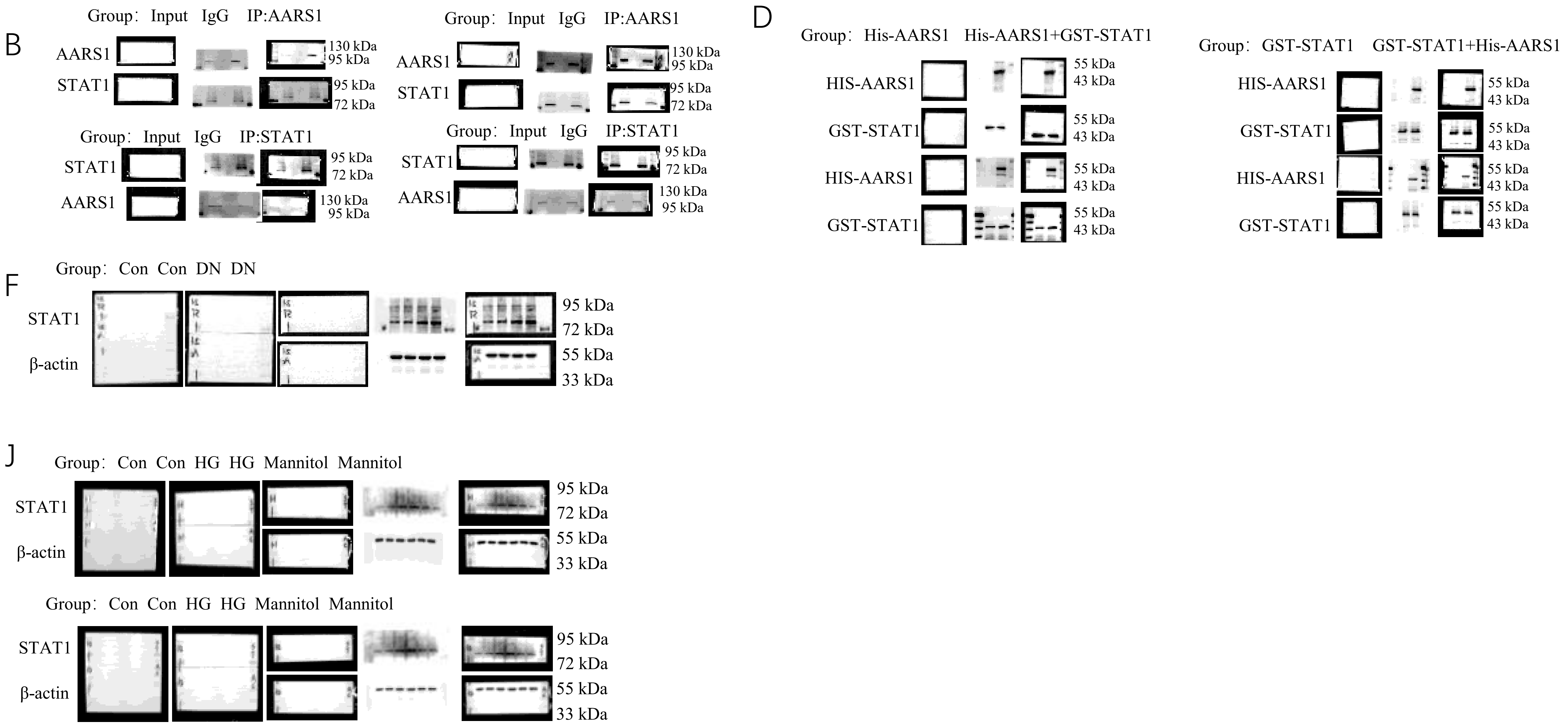
B
Group：Input IgG IP:AARS1
AARS1
130 kDa
95 kDa
STAT1
95 kDa
72 kDa
Group：Input IgG IP:STAT1
STAT1
95 kDa
72 kDa
AARS1
130 kDa
95 kDa
Group：Input IgG IP:AARS1
AARS1
130 kDa
95 kDa
STAT1
95 kDa
72 kDa
Group：Input IgG IP:STAT1
STAT1
95 kDa
72 kDa
AARS1
130 kDa
95 kDa
D
Group：His-AARS1 His-AARS1+GST-STAT1
HIS-AARS1
55 kDa
43 kDa
GST-STAT1
55 kDa
43 kDa
HIS-AARS1
55 kDa
43 kDa
GST-STAT1
55 kDa
43 kDa
Group：GST-STAT1 GST-STAT1+His-AARS1
HIS-AARS1
55 kDa
43 kDa
GST-STAT1
55 kDa
43 kDa
HIS-AARS1
55 kDa
43 kDa
GST-STAT1
55 kDa
43 kDa
F
Group：Con Con DN DN
STAT1
β-actin
95 kDa
72 kDa
55 kDa
33 kDa
J
Group：Con Con HG HG Mannitol Mannitol
STAT1
β-actin
95 kDa
72 kDa
55 kDa
33 kDa
Group：Con Con HG HG Mannitol Mannitol
STAT1
β-actin
95 kDa
72 kDa
55 kDa
33 kDa

Figure 6

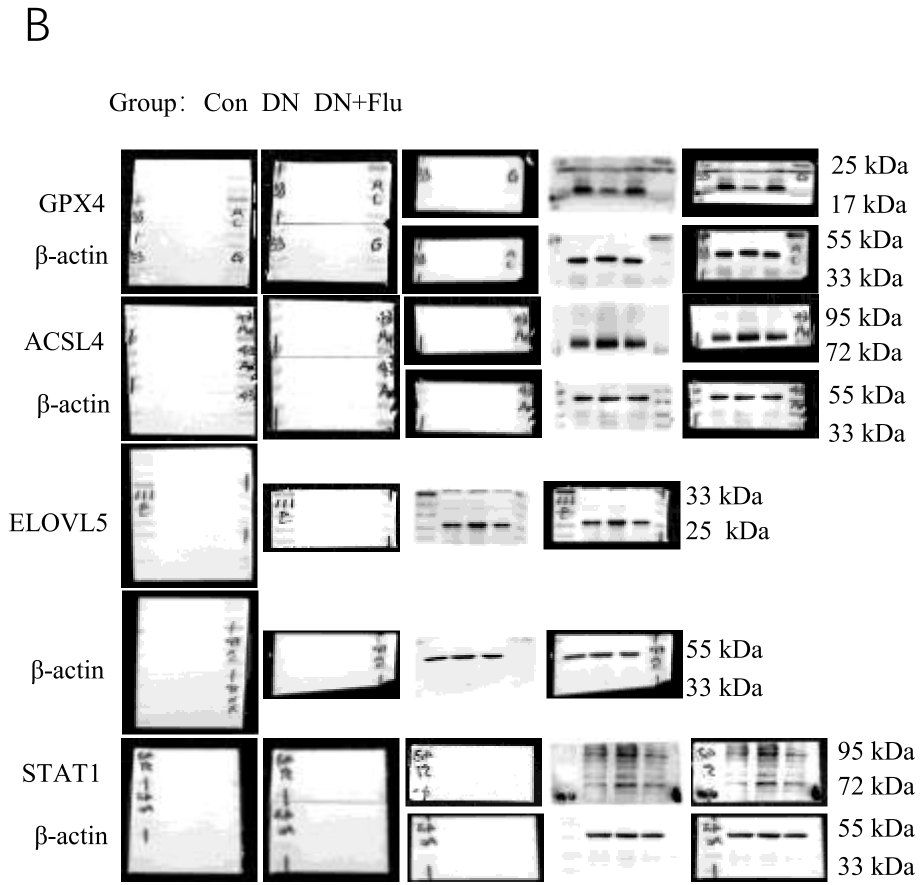
B
Group：Con DN DN+Flu
GPX4
β-actin
ACSL4
β-actin
ELOVL5
β-actin
STAT1
β-actin
25 kDa
17 kDa
55 kDa
33 kDa
95 kDa
72 kDa
55 kDa
33 kDa
33 kDa
25 kDa
55 kDa
33 kDa
95 kDa
72 kDa
55 kDa
33 kDa

Figure 7

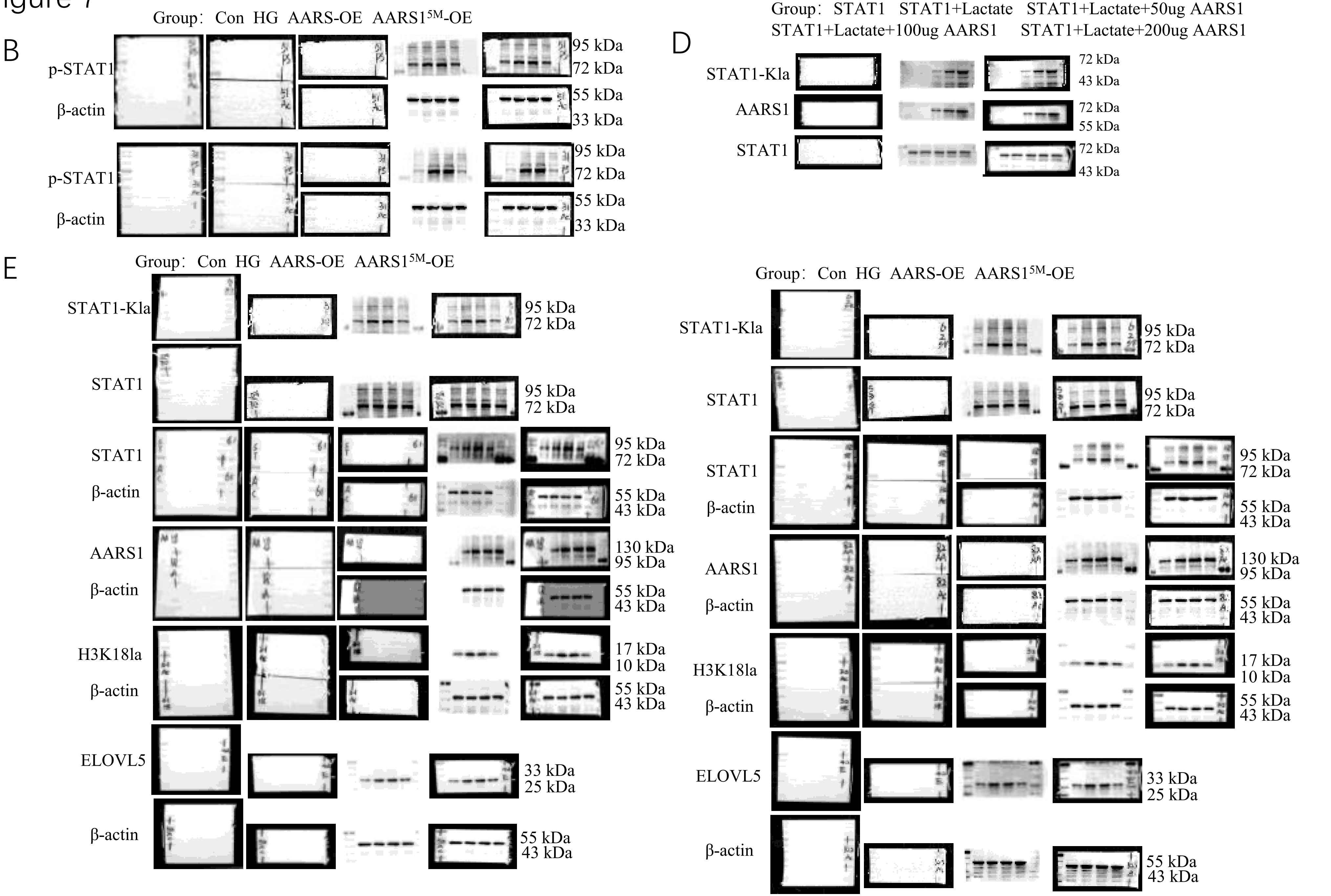
Group: Con HG AARS-OE AARS1^{5M}-OE
B
p-STAT1
β-actin
95 kDa
72 kDa
55 kDa
33 kDa
p-STAT1
β-actin
95 kDa
72 kDa
55 kDa
33 kDa
Group: STAT1 STAT1+Lactate STAT1+Lactate+50ug AARS1
STAT1+Lactate+100ug AARS1 STAT1+Lactate+200ug AARS1
D
STAT1-Kla
72 kDa
43 kDa
AARS1
72 kDa
55 kDa
STAT1
72 kDa
43 kDa
E
Group: Con HG AARS-OE AARS1^{5M}-OE
STAT1-Kla
95 kDa
72 kDa
STAT1
95 kDa
72 kDa
STAT1
β-actin
95 kDa
72 kDa
55 kDa
43 kDa
AARS1
β-actin
130 kDa
95 kDa
55 kDa
43 kDa
H3K18la
β-actin
17 kDa
10 kDa
55 kDa
43 kDa
ELOVL5
33 kDa
25 kDa
β-actin
55 kDa
43 kDa
Group: Con HG AARS-OE AARS1^{5M}-OE
STAT1-Kla
95 kDa
72 kDa
STAT1
95 kDa
72 kDa
STAT1
β-actin
95 kDa
72 kDa
55 kDa
43 kDa
AARS1
β-actin
130 kDa
95 kDa
55 kDa
43 kDa
H3K18la
β-actin
17 kDa
10 kDa
55 kDa
43 kDa
ELOVL5
33 kDa
25 kDa
β-actin
55 kDa
43 kDa

Figure 8

B

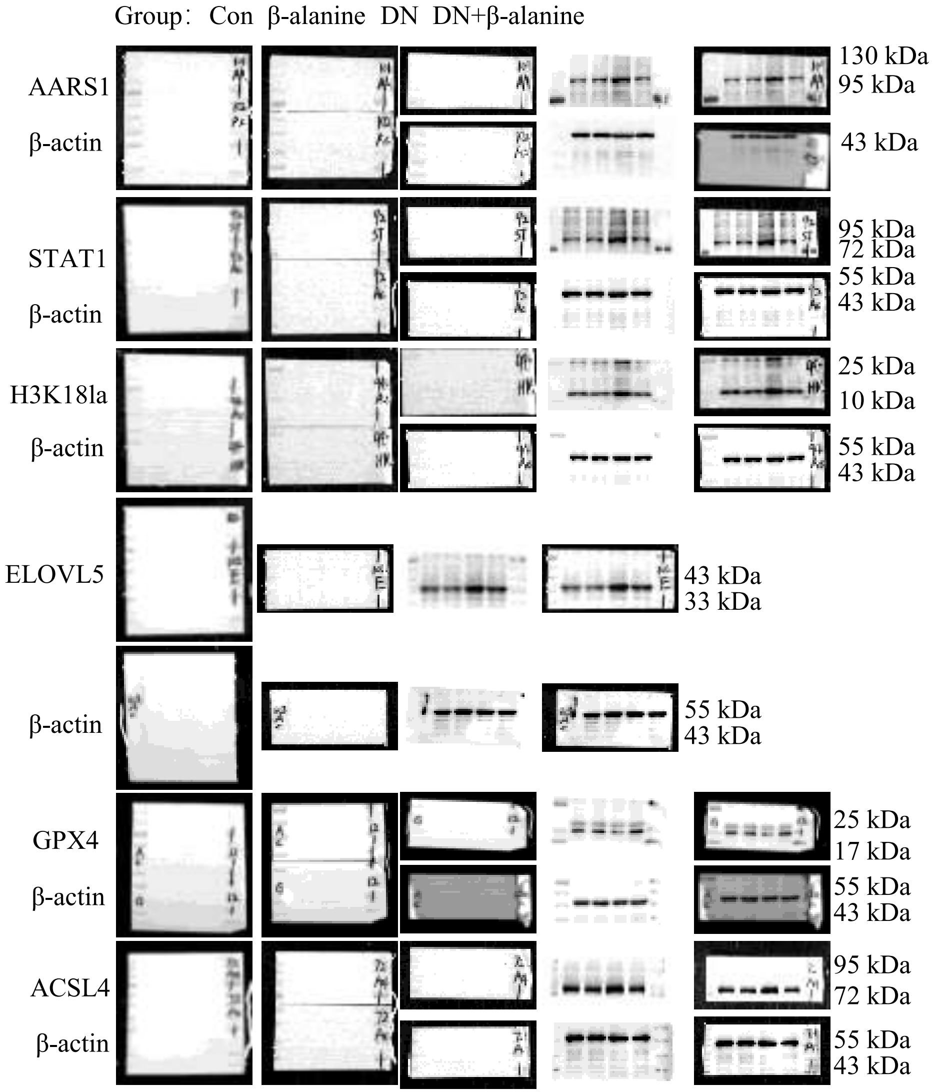
Group: Con β-alanine DN DN+β-alanine
AARS1
β-actin
STAT1
β-actin
H3K18la
β-actin
ELOVL5
β-actin
GPX4
β-actin
ACSL4
β-actin
130 kDa
95 kDa
43 kDa
95 kDa
72 kDa
55 kDa
43 kDa
25 kDa
10 kDa
55 kDa
43 kDa
43 kDa
33 kDa
55 kDa
43 kDa
25 kDa
17 kDa
55 kDa
43 kDa
95 kDa
72 kDa
55 kDa
43 kDa

S-Figure 1

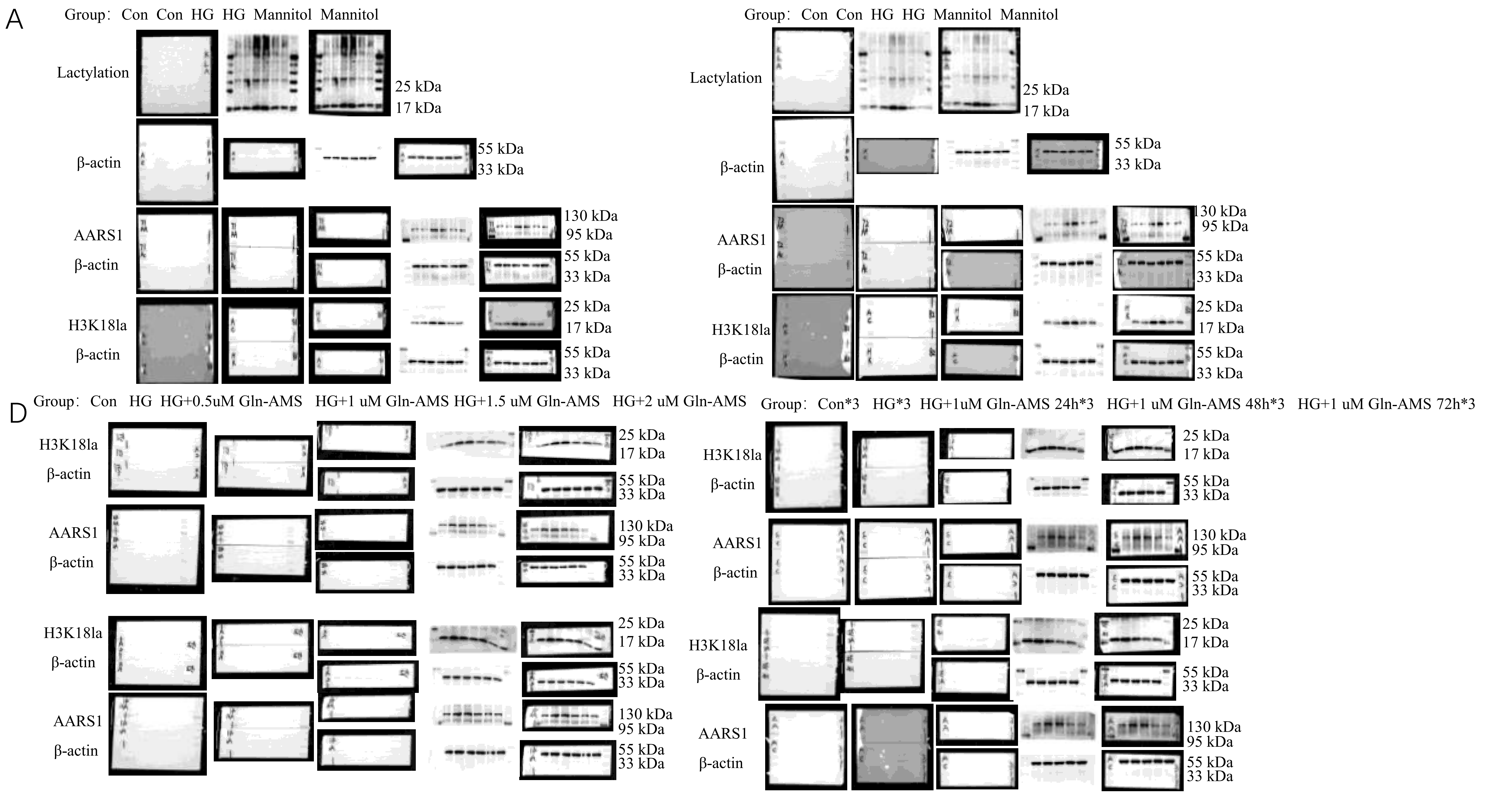
A
Group: Con Con HG HG Mannitol Mannitol
Lactylation
25 kDa
17 kDa
β-actin
55 kDa
33 kDa
AARS1
β-actin
130 kDa
95 kDa
55 kDa
33 kDa
H3K18la
β-actin
25 kDa
17 kDa
55 kDa
33 kDa
Group: Con Con HG HG Mannitol Mannitol
Lactylation
25 kDa
17 kDa
β-actin
55 kDa
33 kDa
AARS1
β-actin
130 kDa
95 kDa
55 kDa
33 kDa
H3K18la
β-actin
25 kDa
17 kDa
55 kDa
33 kDa
D
Group: Con HG HG+0.5uM Gln-AMS HG+1 uM Gln-AMS HG+1.5 uM Gln-AMS HG+2 uM Gln-AMS
H3K18la
β-actin
25 kDa
17 kDa
55 kDa
33 kDa
AARS1
β-actin
130 kDa
95 kDa
55 kDa
33 kDa
H3K18la
β-actin
25 kDa
17 kDa
55 kDa
33 kDa
AARS1
β-actin
130 kDa
95 kDa
55 kDa
33 kDa
Group: Con*3 HG*3 HG+1uM Gln-AMS 24h*3 HG+1 uM Gln-AMS 48h*3 HG+1 uM Gln-AMS 72h*3
H3K18la
β-actin
25 kDa
17 kDa
55 kDa
33 kDa
AARS1
β-actin
130 kDa
95 kDa
55 kDa
33 kDa
H3K18la
β-actin
25 kDa
17 kDa
55 kDa
33 kDa
AARS1
β-actin
130 kDa
95 kDa
55 kDa
33 kDa

S-Figure 2

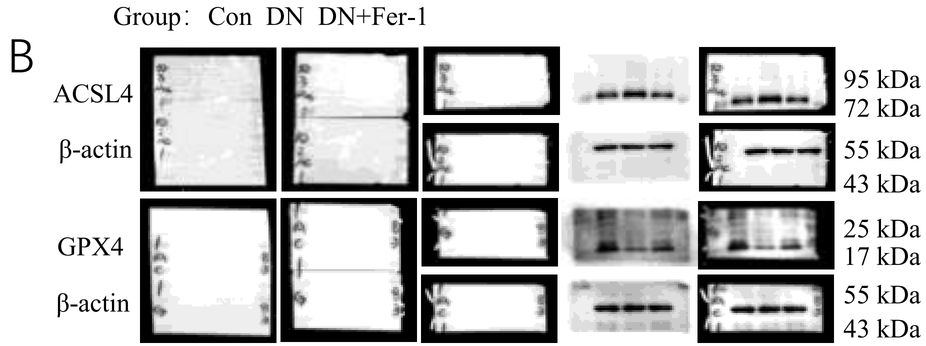
Group: Con DN DN+Fer-1
B
ACSL4
β-actin
GPX4
β-actin
95 kDa
72 kDa
55 kDa
43 kDa
25 kDa
17 kDa
55 kDa
43 kDa

S-Figure 3

B

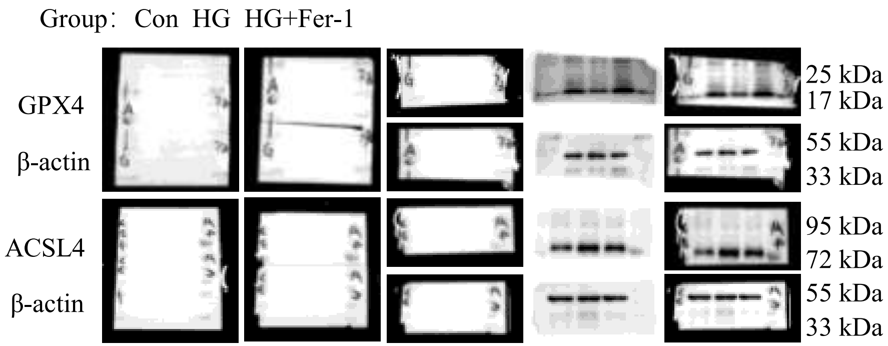

Group: Con HG HG+Fer-1
GPX4
β-actin
ACSL4
β-actin
25 kDa
17 kDa
55 kDa
33 kDa
95 kDa
72 kDa
55 kDa
33 kDa

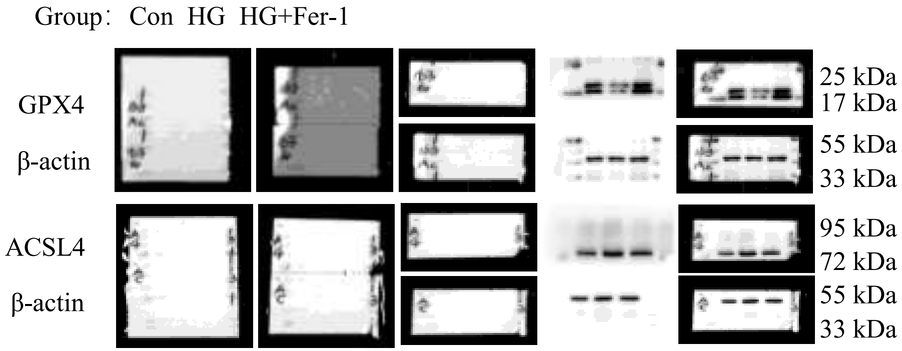

Group: Con HG HG+Fer-1
GPX4
β-actin
ACSL4
β-actin
25 kDa
17 kDa
55 kDa
33 kDa
95 kDa
72 kDa
55 kDa
33 kDa

S-Figure 4

A

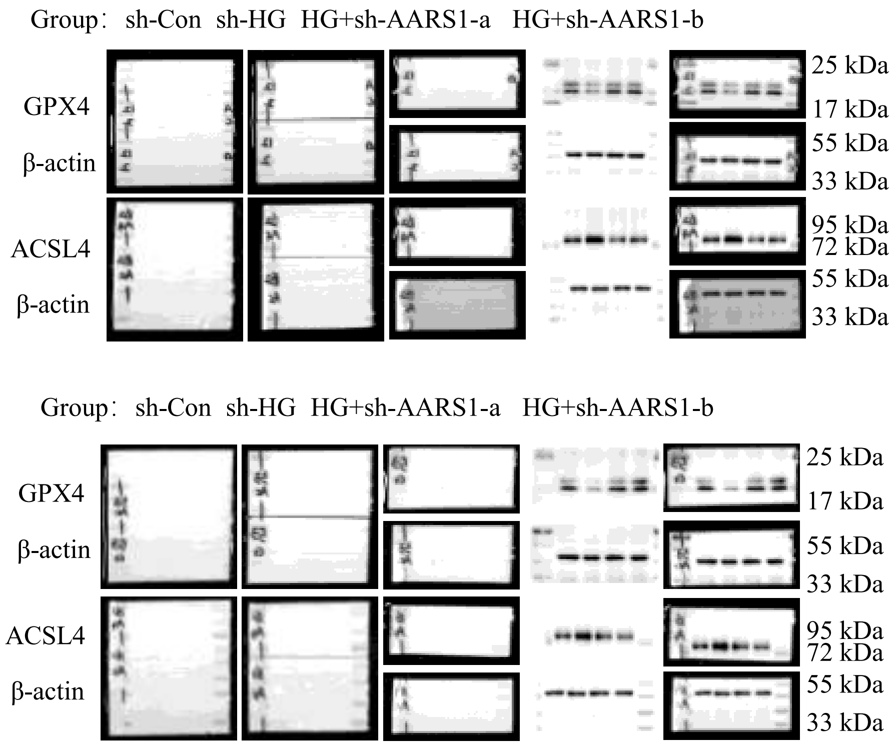
Group: sh-Con sh-HG HG+sh-AARS1-a HG+sh-AARS1-b
GPX4
β-actin
ACSL4
β-actin
25 kDa
17 kDa
55 kDa
33 kDa
95 kDa
72 kDa
55 kDa
33 kDa
Group: sh-Con sh-HG HG+sh-AARS1-a HG+sh-AARS1-b
GPX4
β-actin
ACSL4
β-actin
25 kDa
17 kDa
55 kDa
33 kDa
95 kDa
72 kDa
55 kDa
33 kDa

S-Figure 5

A

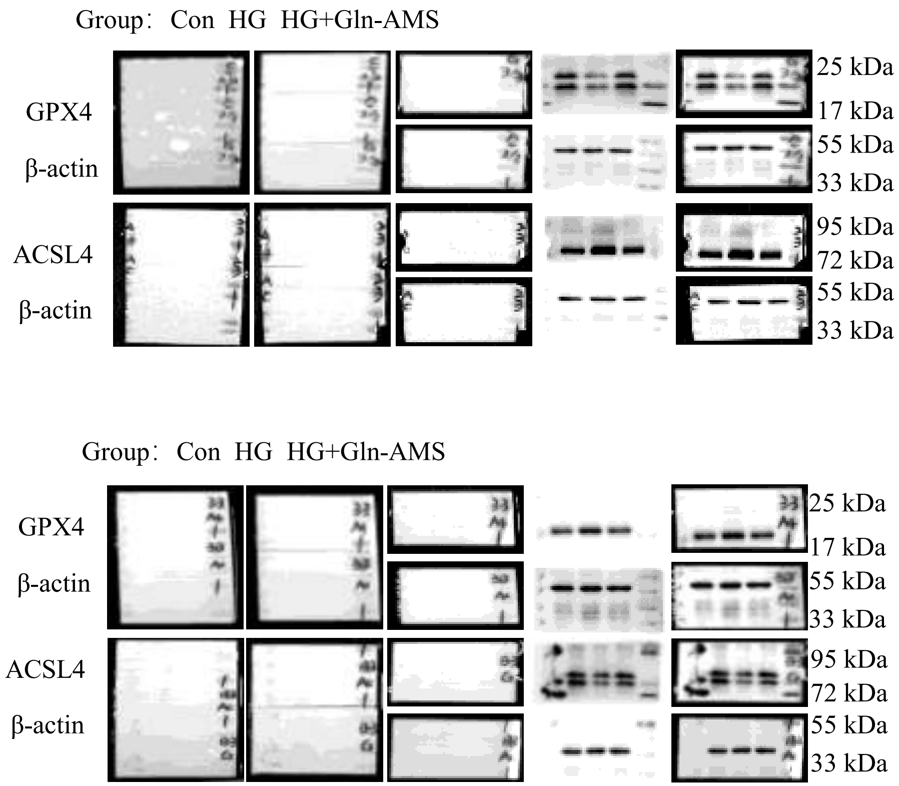
Group: Con HG HG+Gln-AMS
GPX4
β-actin
ACSL4
β-actin
25 kDa
17 kDa
55 kDa
33 kDa
95 kDa
72 kDa
55 kDa
33 kDa
Group: Con HG HG+Gln-AMS
GPX4
β-actin
ACSL4
β-actin
25 kDa
17 kDa
55 kDa
33 kDa
95 kDa
72 kDa
55 kDa
33 kDa

S-Figure 6

F

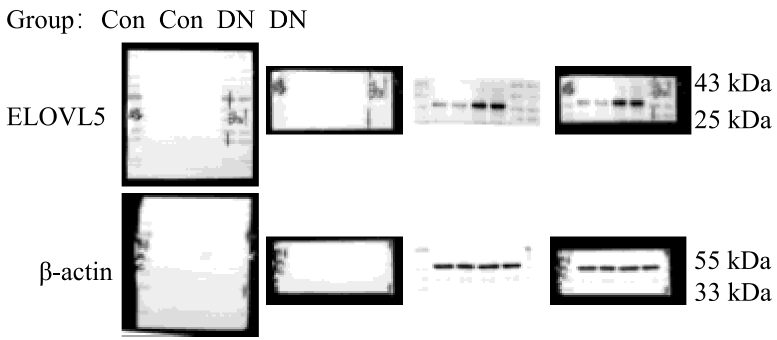
Group: Con Con DN DN
ELOVL5
43 kDa
25 kDa
β-actin
55 kDa
33 kDa

G

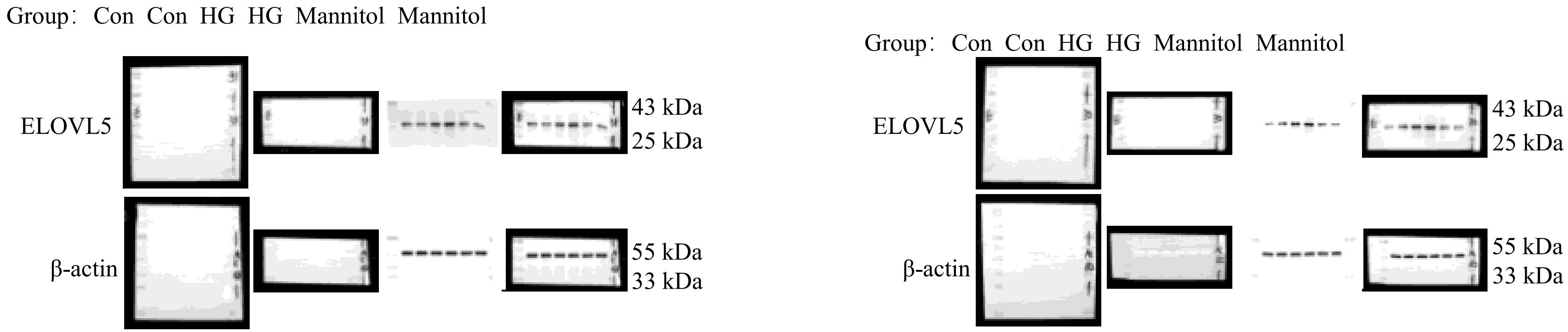
Group: Con Con HG HG Mannitol Mannitol
ELOVL5
43 kDa
25 kDa
β-actin
55 kDa
33 kDa
Group: Con Con HG HG Mannitol Mannitol
ELOVL5
43 kDa
25 kDa
β-actin
55 kDa
33 kDa

S-Figure 7

A

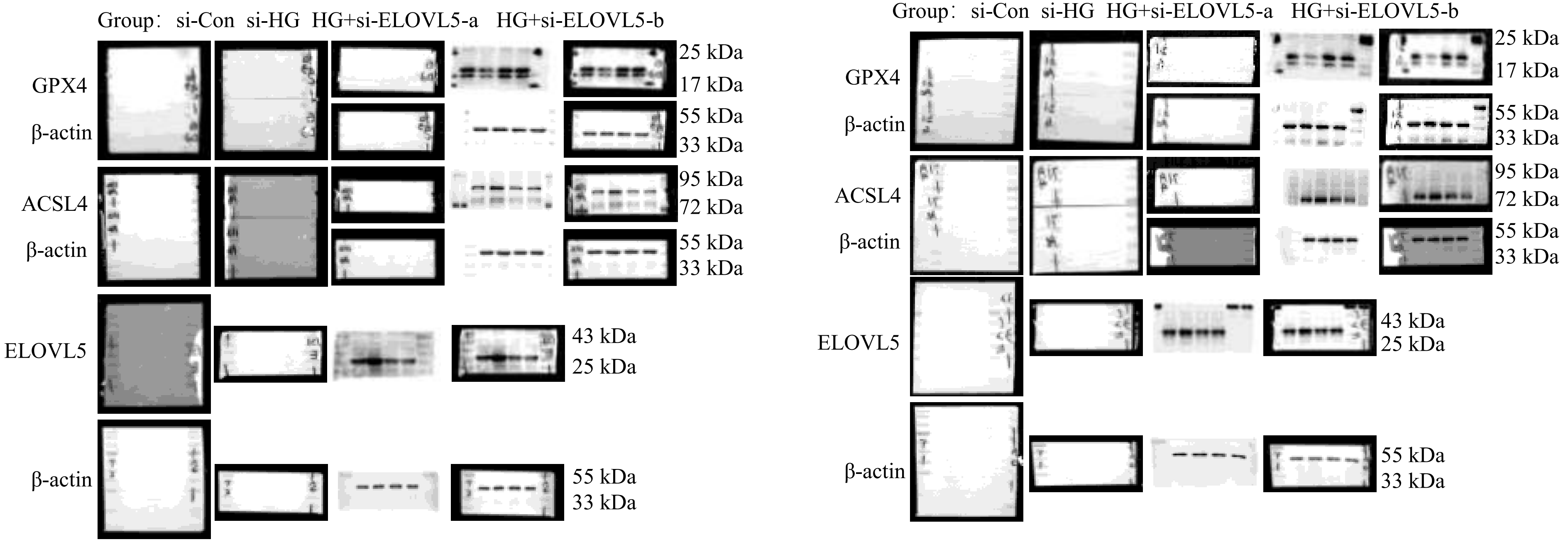
Group: si-Con si-HG HG+si-ELOVL5-a HG+si-ELOVL5-b
GPX4
β-actin
ACSL4
β-actin
ELOVL5
β-actin
25 kDa
17 kDa
55 kDa
33 kDa
95 kDa
72 kDa
55 kDa
33 kDa
43 kDa
25 kDa
55 kDa
33 kDa
Group: si-Con si-HG HG+si-ELOVL5-a HG+si-ELOVL5-b
GPX4
β-actin
ACSL4
β-actin
ELOVL5
β-actin
25 kDa
17 kDa
55 kDa
33 kDa
95 kDa
72 kDa
55 kDa
33 kDa
43 kDa
25 kDa
55 kDa
33 kDa

S-Figure 9

A

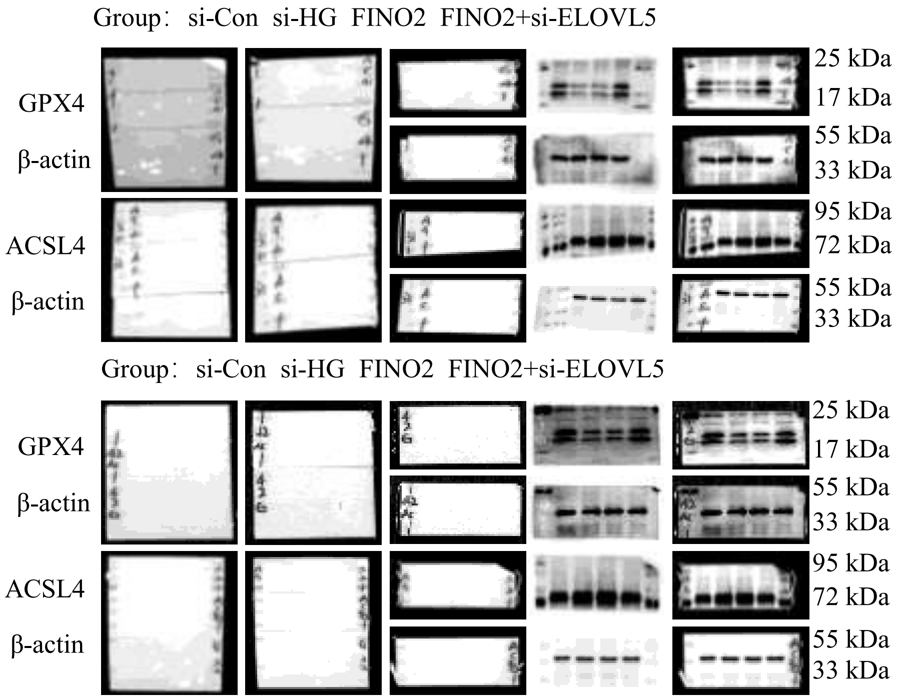
Group：si-Con si-HG FINO2 FINO2+si-ELOVL5
GPX4
β-actin
ACSL4
β-actin
25 kDa
17 kDa
55 kDa
33 kDa
95 kDa
72 kDa
55 kDa
33 kDa
Group：si-Con si-HG FINO2 FINO2+si-ELOVL5
GPX4
β-actin
ACSL4
β-actin
25 kDa
17 kDa
55 kDa
33 kDa
95 kDa
72 kDa
55 kDa
33 kDa

S-Figure 10

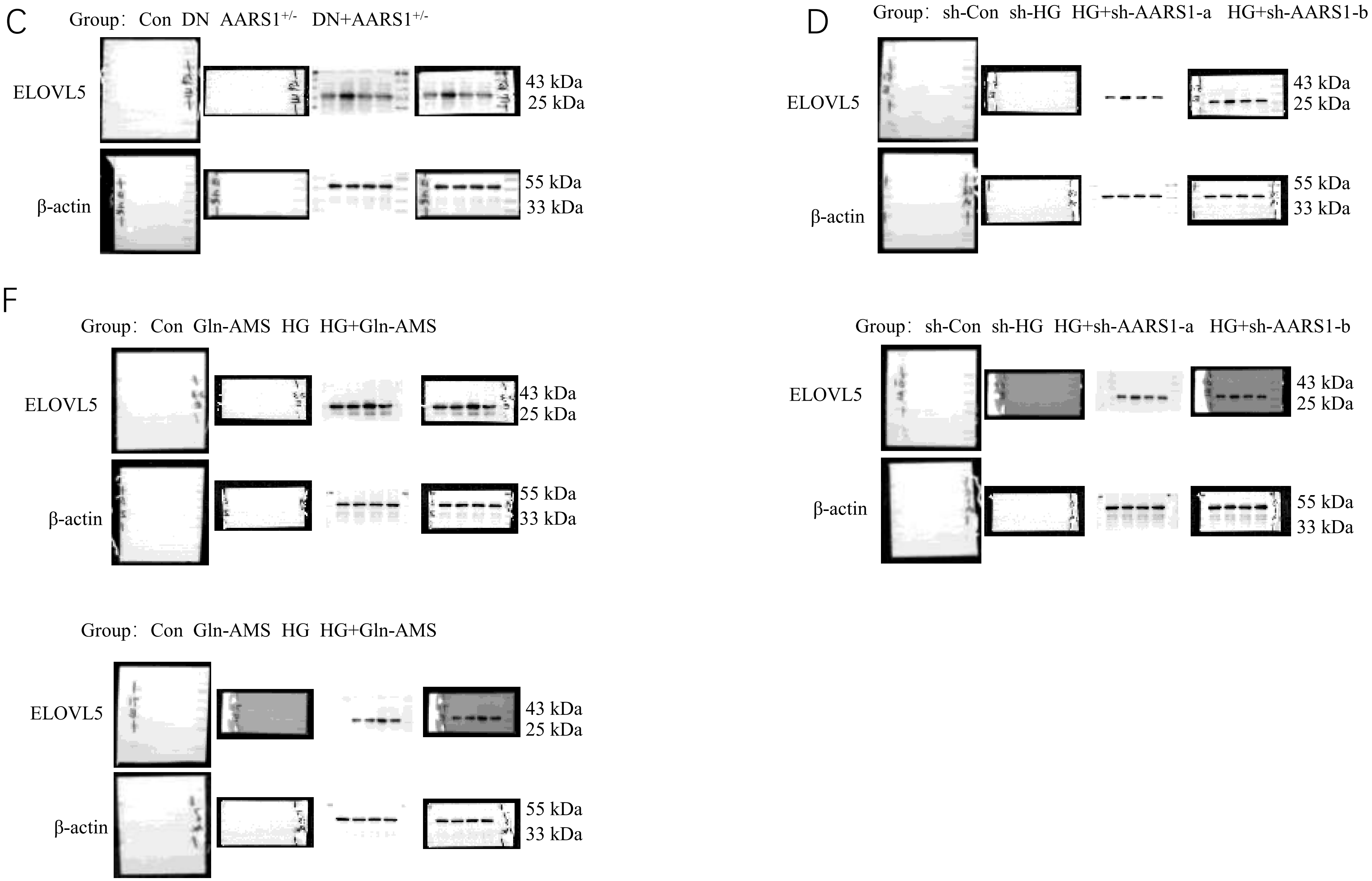
C
Group: Con DN AARS1+/- DN+AARS1+/-
ELOVL5
43 kDa
25 kDa
β-actin
55 kDa
33 kDa
D
Group: sh-Con sh-HG HG+sh-AARS1-a HG+sh-AARS1-b
ELOVL5
43 kDa
25 kDa
β-actin
55 kDa
33 kDa
F
Group: Con Gln-AMS HG HG+Gln-AMS
ELOVL5
43 kDa
25 kDa
β-actin
55 kDa
33 kDa
Group: sh-Con sh-HG HG+sh-AARS1-a HG+sh-AARS1-b
ELOVL5
43 kDa
25 kDa
β-actin
55 kDa
33 kDa
Group: Con Gln-AMS HG HG+Gln-AMS
ELOVL5
43 kDa
25 kDa
β-actin
55 kDa
33 kDa

S-Figure 11

A

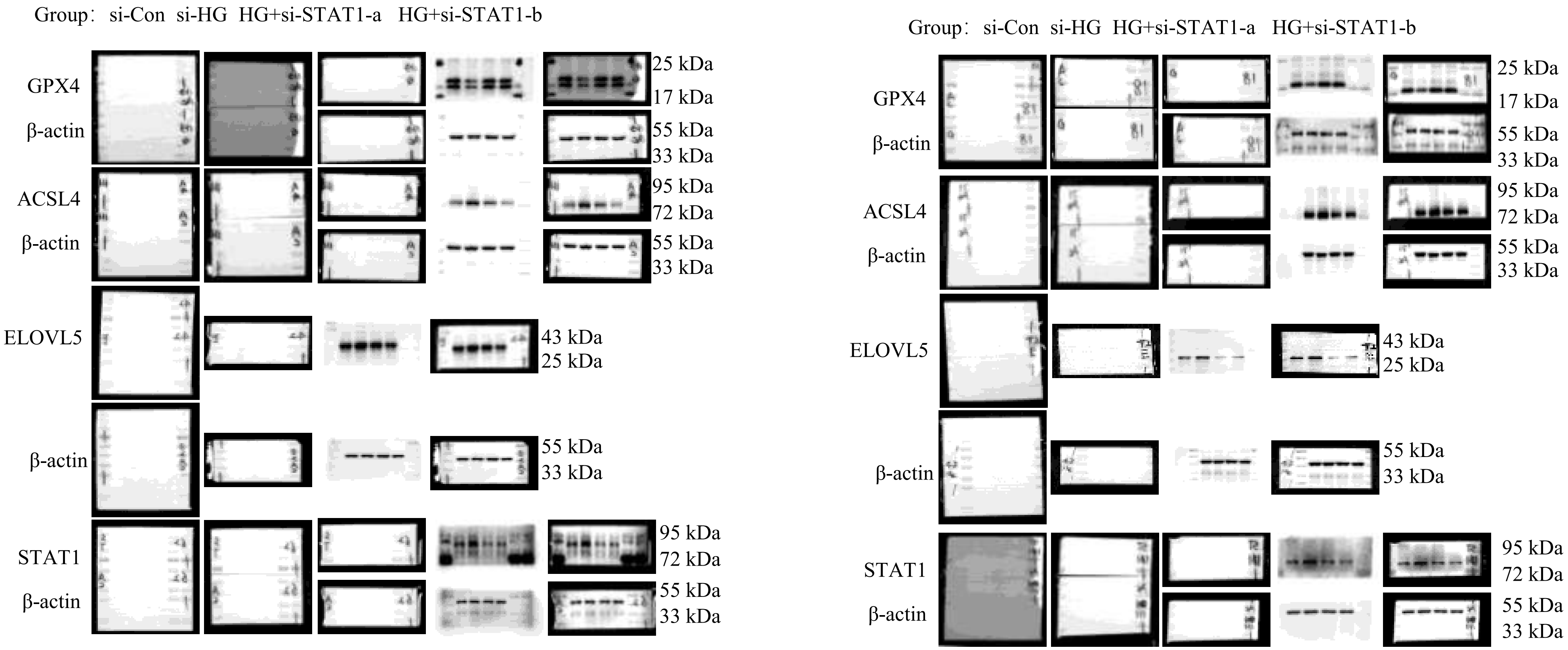
Group: si-Con si-HG HG+si-STAT1-a HG+si-STAT1-b
GPX4
β-actin
ACSL4
β-actin
ELOVL5
β-actin
STAT1
β-actin
25 kDa
17 kDa
55 kDa
33 kDa
95 kDa
72 kDa
55 kDa
33 kDa
43 kDa
25 kDa
55 kDa
33 kDa
95 kDa
72 kDa
55 kDa
33 kDa
Group: si-Con si-HG HG+si-STAT1-a HG+si-STAT1-b
GPX4
β-actin
ACSL4
β-actin
ELOVL5
β-actin
STAT1
β-actin
25 kDa
17 kDa
55 kDa
33 kDa
95 kDa
72 kDa
55 kDa
33 kDa
43 kDa
25 kDa
55 kDa
33 kDa
95 kDa
72 kDa
55 kDa
33 kDa

S-Figure 12

A

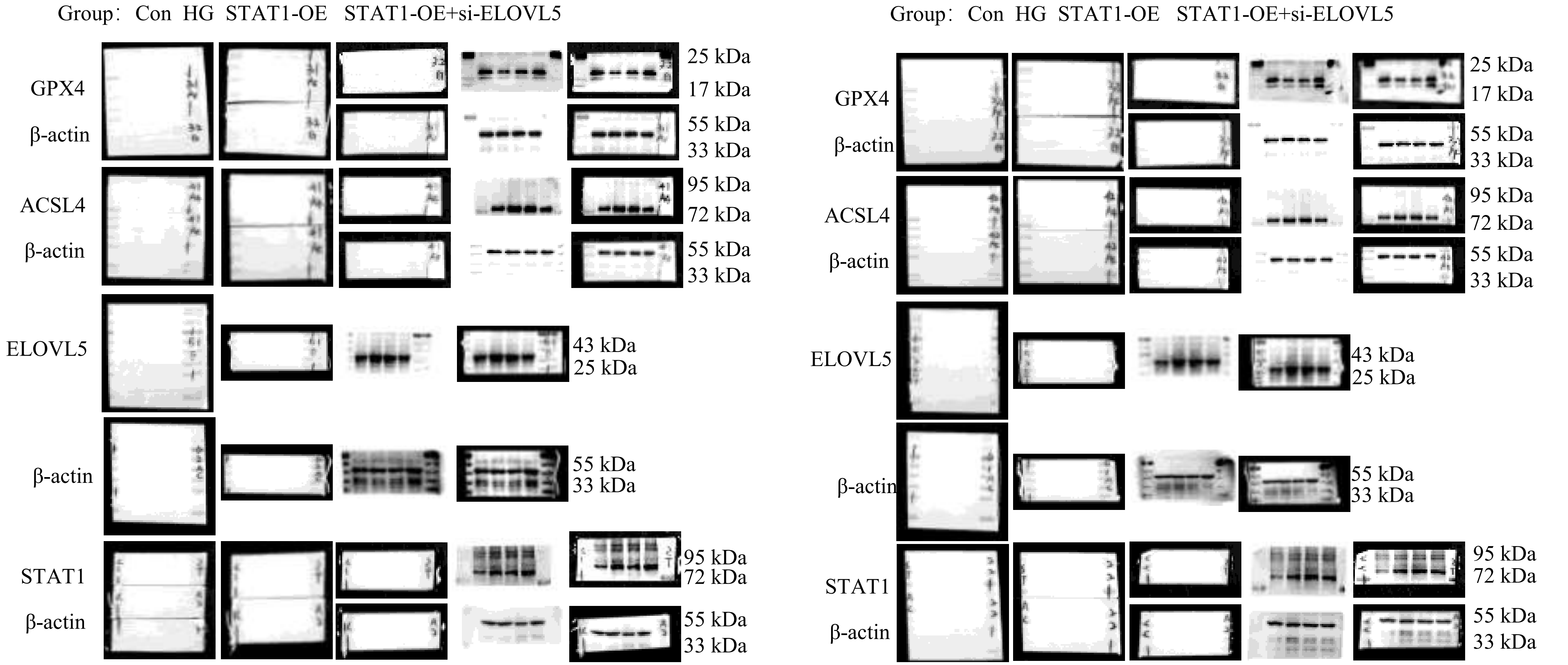
Group: Con HG STAT1-OE STAT1-OE+si-ELOVL5
GPX4
β-actin
ACSL4
β-actin
ELOVL5
β-actin
STAT1
β-actin
25 kDa
17 kDa
55 kDa
33 kDa
95 kDa
72 kDa
55 kDa
33 kDa
43 kDa
25 kDa
55 kDa
33 kDa
95 kDa
72 kDa
55 kDa
33 kDa
Group: Con HG STAT1-OE STAT1-OE+si-ELOVL5
GPX4
β-actin
ACSL4
β-actin
ELOVL5
β-actin
STAT1
β-actin

S-Figure 14

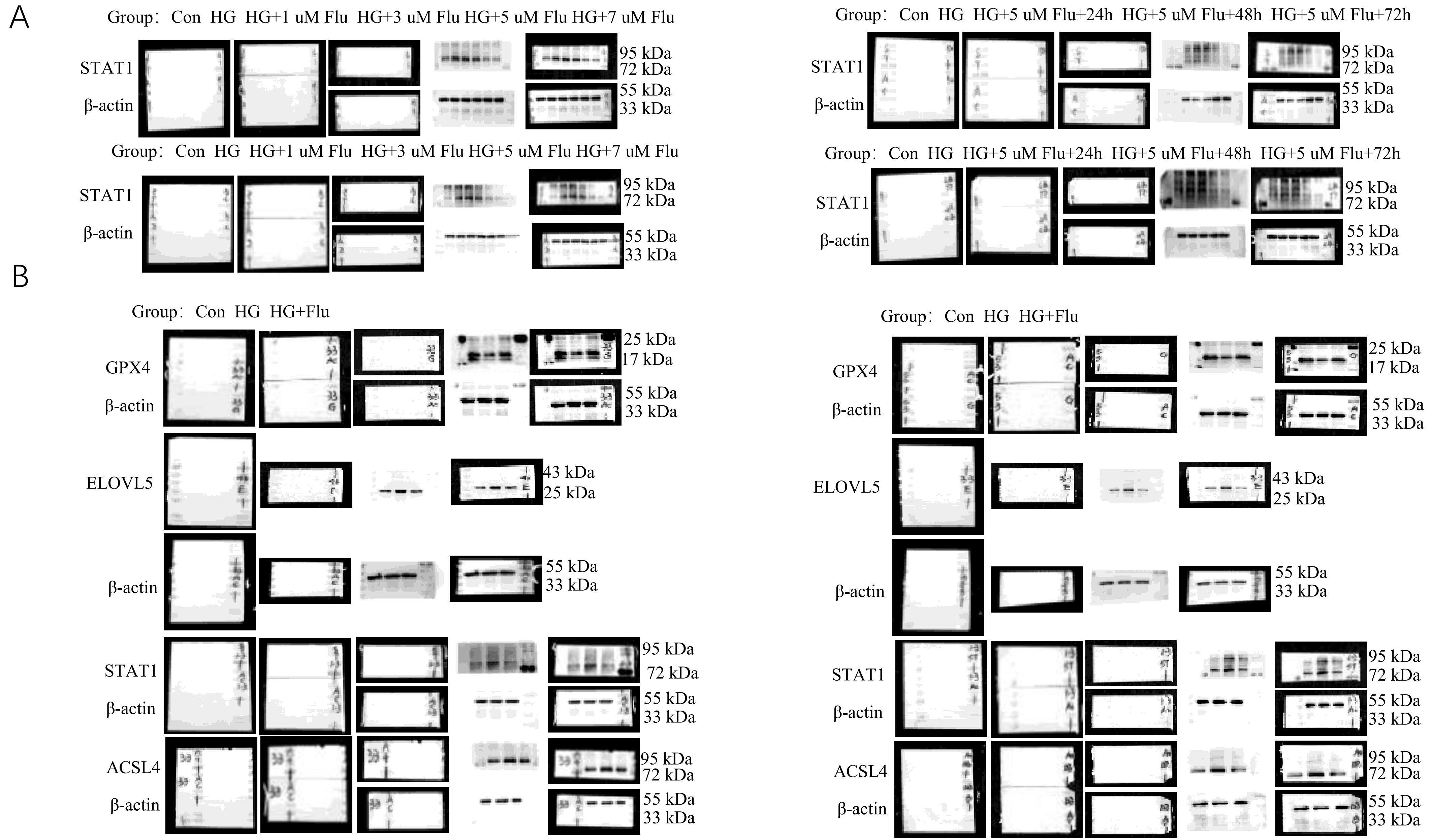
A
Group: Con HG HG+1 uM Flu HG+3 uM Flu HG+5 uM Flu HG+7 uM Flu
STAT1
β-actin
95 kDa
72 kDa
55 kDa
33 kDa
Group: Con HG HG+5 uM Flu+24h HG+5 uM Flu+48h HG+5 uM Flu+72h
B
Group: Con HG HG+Flu
GPX4
25 kDa
17 kDa
ELOVL5
43 kDa
25 kDa
ACSL4

S-Figure 15

A

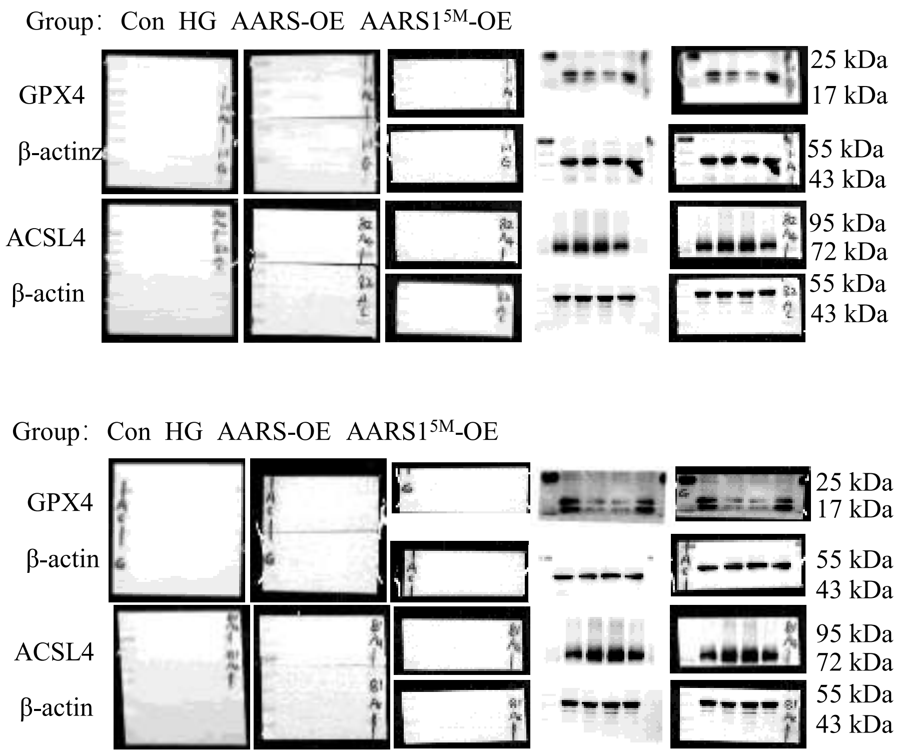
Group: Con HG AARS-OE AARS1^{5M}-OE
GPX4
β-actinz
ACSL4
β-actin
25 kDa
17 kDa
55 kDa
43 kDa
95 kDa
72 kDa
55 kDa
43 kDa
Group: Con HG AARS-OE AARS1^{5M}-OE
GPX4
β-actin
ACSL4
β-actin
25 kDa
17 kDa
55 kDa
43 kDa
95 kDa
72 kDa
55 kDa
43 kDa

S-Figure 16

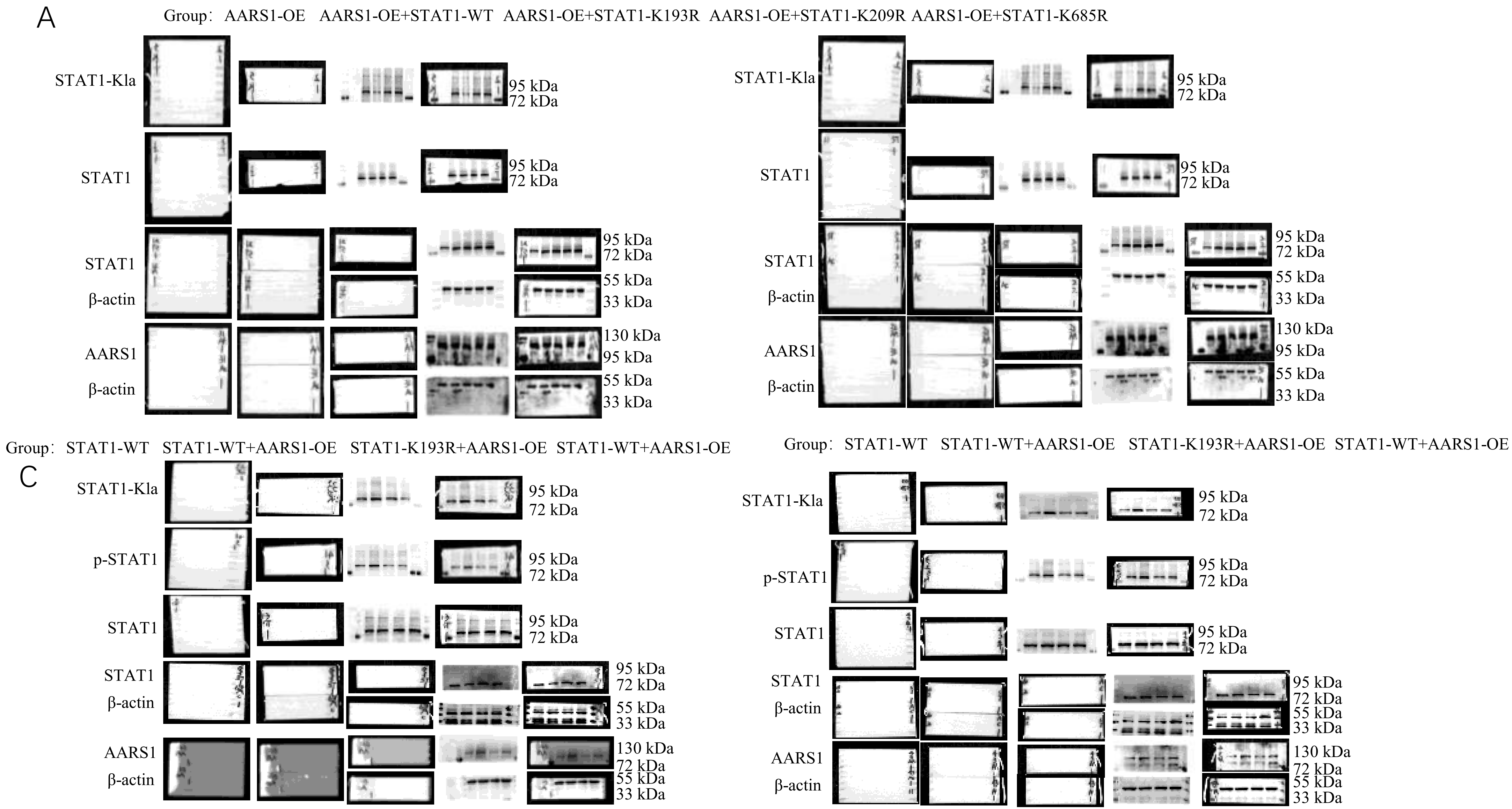
A
Group: AARS1-OE AARS1-OE+STAT1-WT AARS1-OE+STAT1-K193R AARS1-OE+STAT1-K209R AARS1-OE+STAT1-K685R
STAT1-Kla
STAT1
STAT1
β-actin
AARS1
β-actin
95 kDa
72 kDa
55 kDa
33 kDa
130 kDa
C
Group: STAT1-WT STAT1-WT+AARS1-OE STAT1-K193R+AARS1-OE STAT1-WT+AARS1-OE
STAT1-Kla
p-STAT1
STAT1
STAT1
β-actin
AARS1
β-actin
95 kDa
72 kDa
55 kDa
33 kDa
130 kDa

S-Figure 17

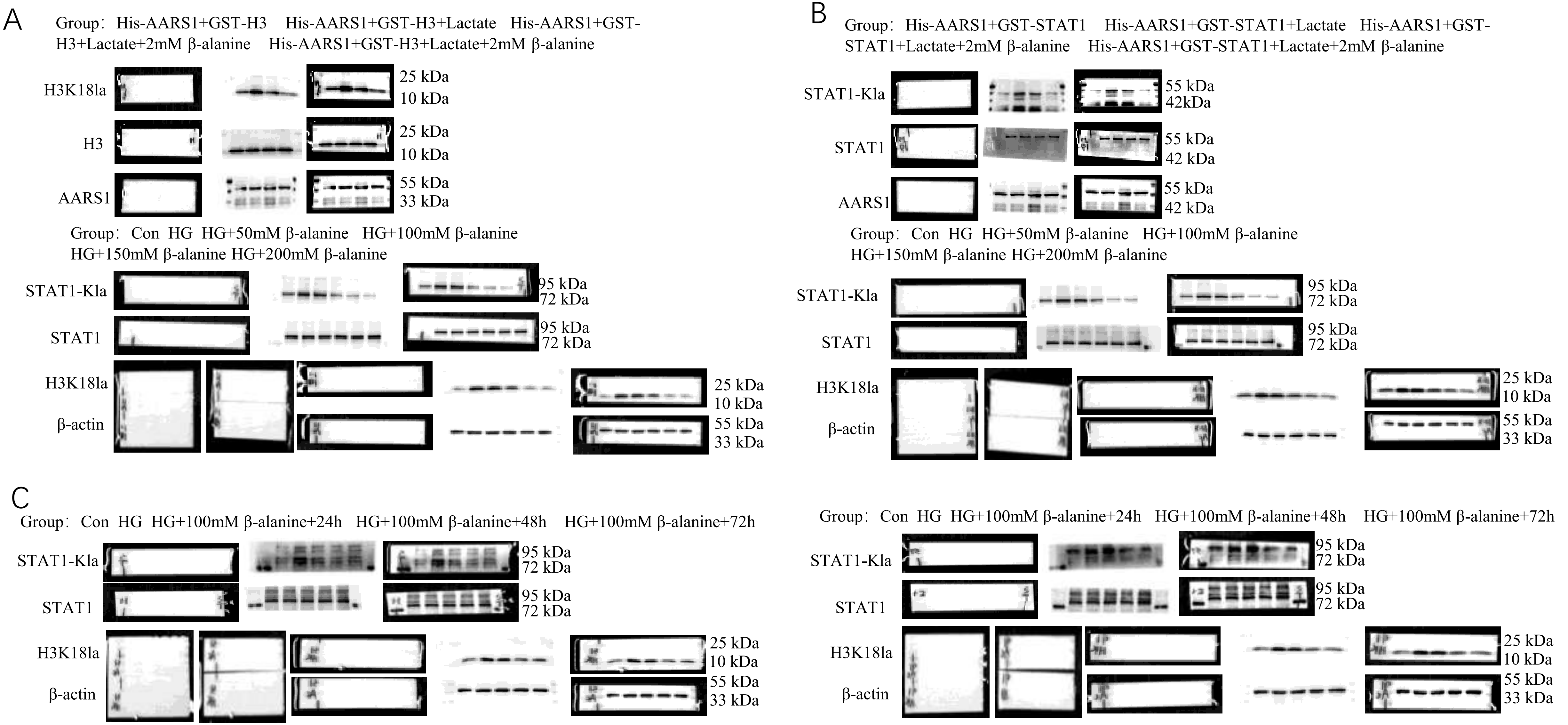
A
Group：His-AARS1+GST-H3 His-AARS1+GST-H3+Lactate His-AARS1+GST-H3+Lactate+2mM β-alanine His-AARS1+GST-H3+Lactate+2mM β-alanine
H3K18la
25 kDa
10 kDa
H3
25 kDa
10 kDa
AARS1
55 kDa
33 kDa
Group：Con HG HG+50mM β-alanine HG+100mM β-alanine HG+150mM β-alanine HG+200mM β-alanine
STAT1-Kla
95 kDa
72 kDa
STAT1
95 kDa
72 kDa
H3K18la
25 kDa
10 kDa
β-actin
55 kDa
33 kDa
B
Group：His-AARS1+GST-STAT1 His-AARS1+GST-STAT1+Lactate His-AARS1+GST-STAT1+Lactate+2mM β-alanine His-AARS1+GST-STAT1+Lactate+2mM β-alanine
STAT1-Kla
55 kDa
42kDa
STAT1
55 kDa
42 kDa
AARS1
55 kDa
42 kDa
Group：Con HG HG+50mM β-alanine HG+100mM β-alanine HG+150mM β-alanine HG+200mM β-alanine
STAT1-Kla
95 kDa
72 kDa
STAT1
95 kDa
72 kDa
H3K18la
25 kDa
10 kDa
β-actin
55 kDa
33 kDa
C
Group：Con HG HG+100mM β-alanine+24h HG+100mM β-alanine+48h HG+100mM β-alanine+72h
STAT1-Kla
95 kDa
72 kDa
STAT1
95 kDa
72 kDa
H3K18la
25 kDa
10 kDa
β-actin
55 kDa
33 kDa
Group：Con HG HG+100mM β-alanine+24h HG+100mM β-alanine+48h HG+100mM β-alanine+72h
STAT1-Kla
95 kDa
72 kDa
STAT1
95 kDa
72 kDa
H3K18la
25 kDa
10 kDa
β-actin
55 kDa
33 kDa

S-Figure 18

A

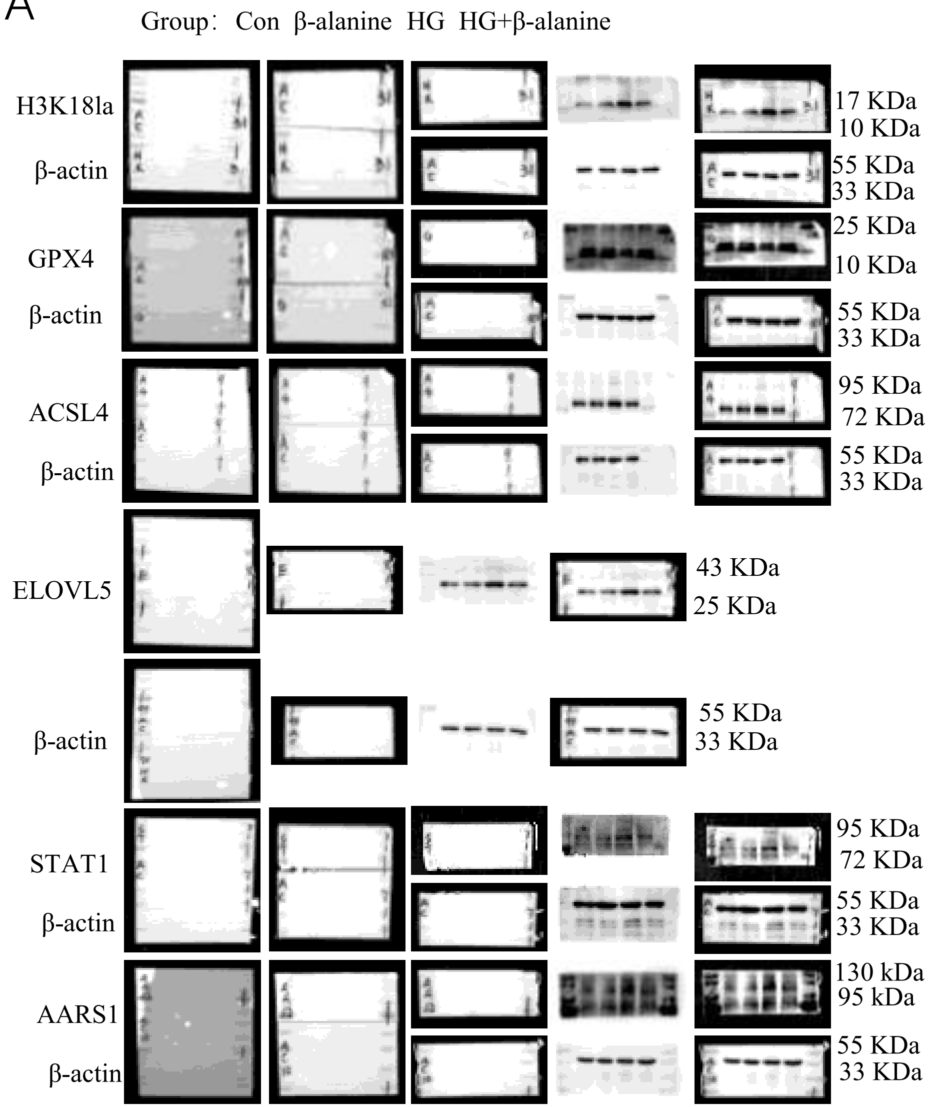

Group: Con β-alanine HG HG+β-alanine
H3K18la
β-actin
17 KDa
10 KDa
55 KDa
33 KDa
GPX4
β-actin
25 KDa
10 KDa
55 KDa
33 KDa
ACSL4
β-actin
95 KDa
72 KDa
55 KDa
33 KDa
ELOVL5
43 KDa
25 KDa
β-actin
55 KDa
33 KDa
STAT1
β-actin
95 KDa
72 KDa
55 KDa
33 KDa
AARS1
β-actin
130 kDa
95 kDa
55 KDa
33 KDa

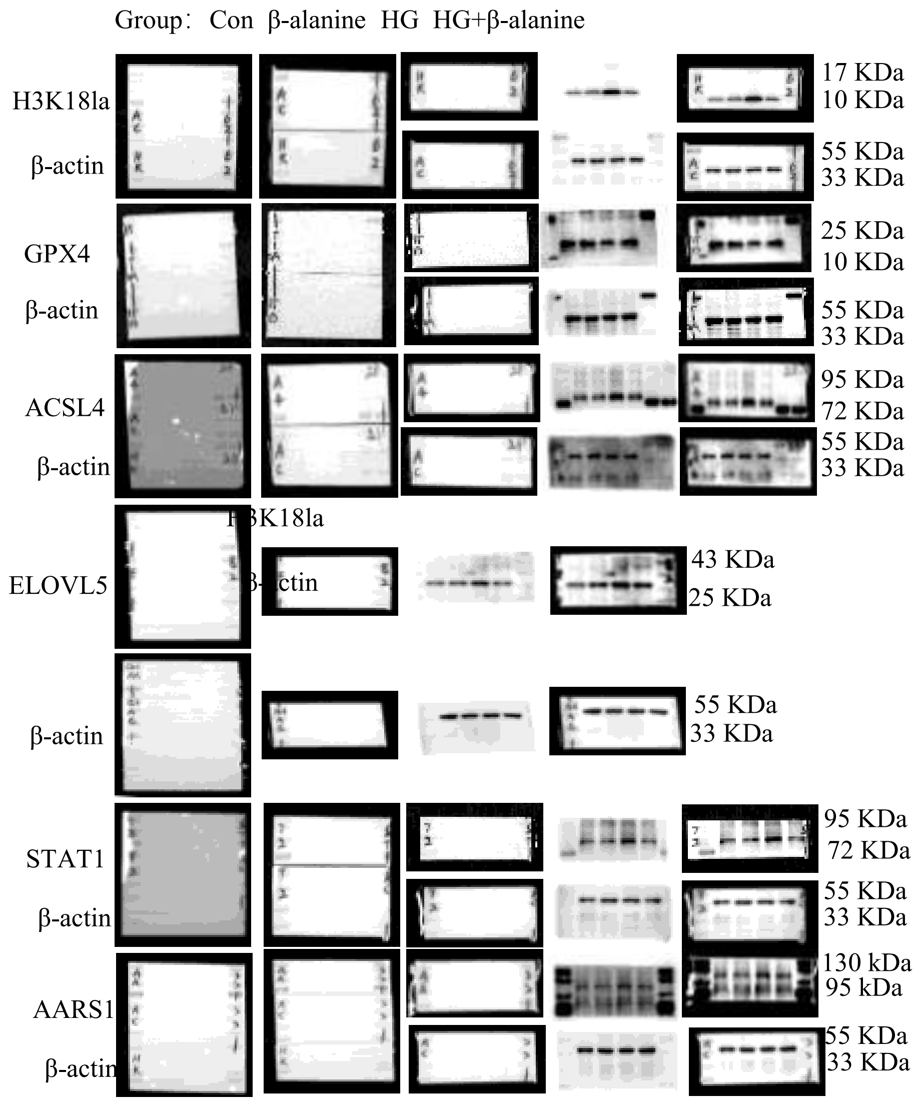

Group: Con β-alanine HG HG+β-alanine
H3K18la
β-actin
17 KDa
10 KDa
55 KDa
33 KDa
GPX4
β-actin
25 KDa
10 KDa
55 KDa
33 KDa
ACSL4
β-actin
95 KDa
72 KDa
55 KDa
33 KDa
ELOVL5
43 KDa
25 KDa
β-actin
55 KDa
33 KDa
STAT1
β-actin
95 KDa
72 KDa
55 KDa
33 KDa
AARS1
β-actin
130 kDa
95 kDa
55 KDa
33 KDa

Figure 1 Repeated measurements

C

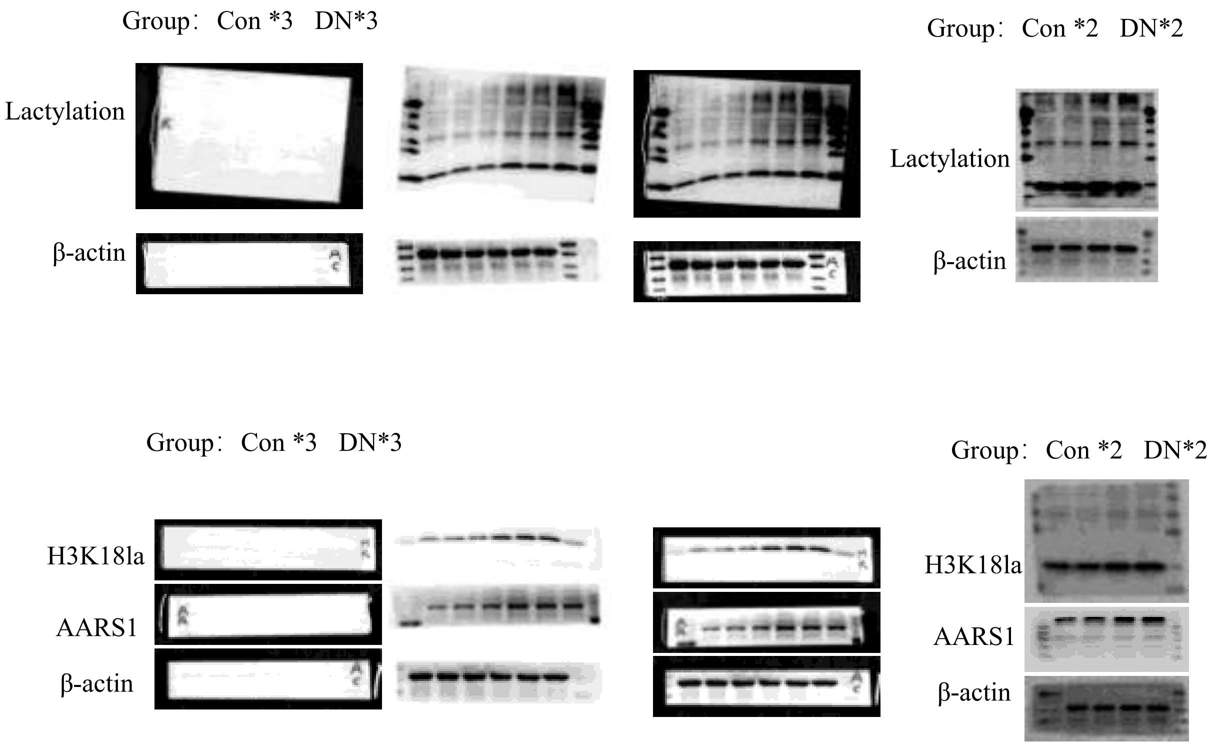

Figure 2 Repeated measurements

B

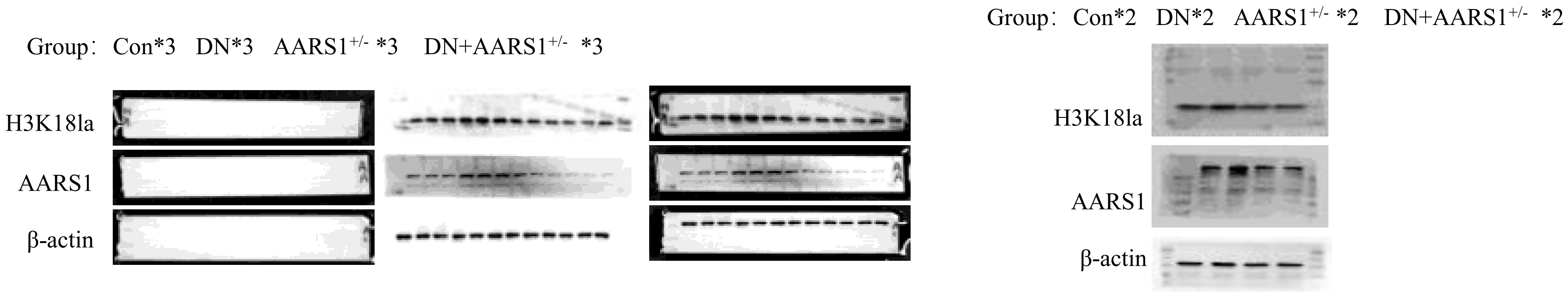

D

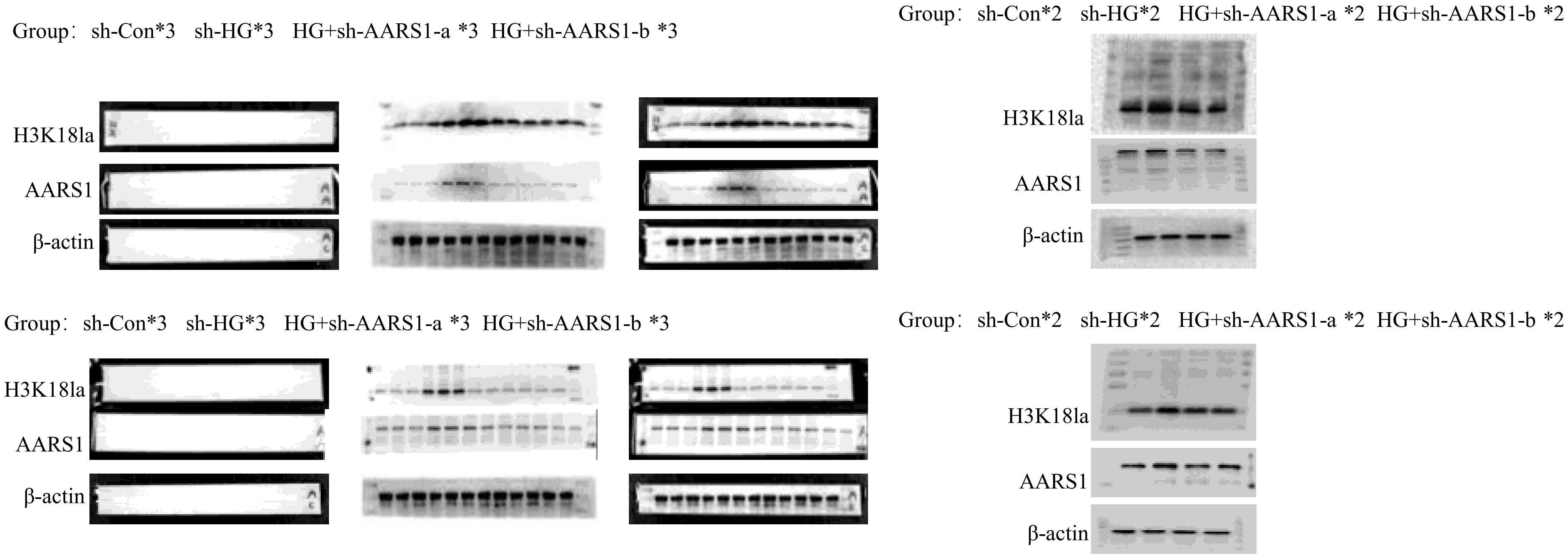

Figure 3 Repeated measurements

D

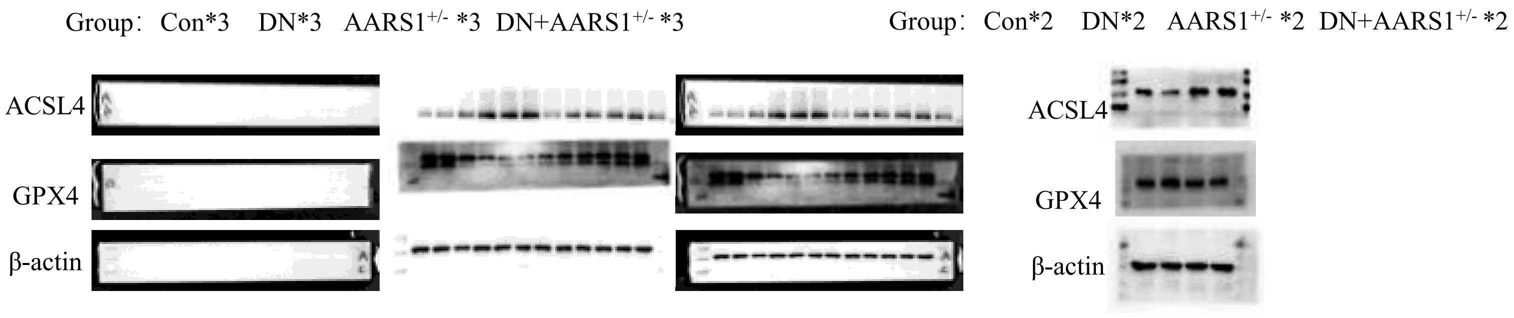

Figure 4 Repeated measurements

A

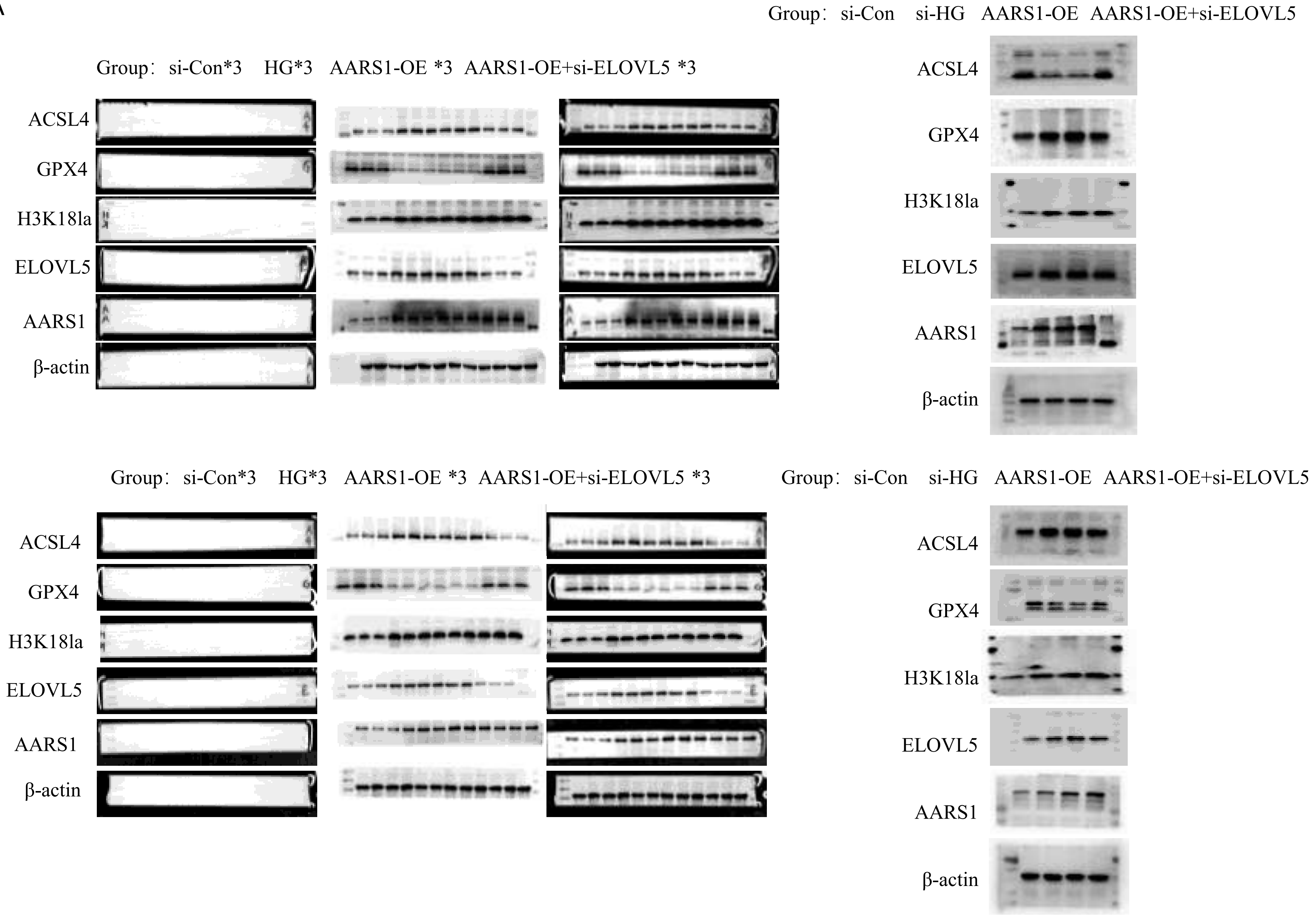

Figure 5 Repeated measurements

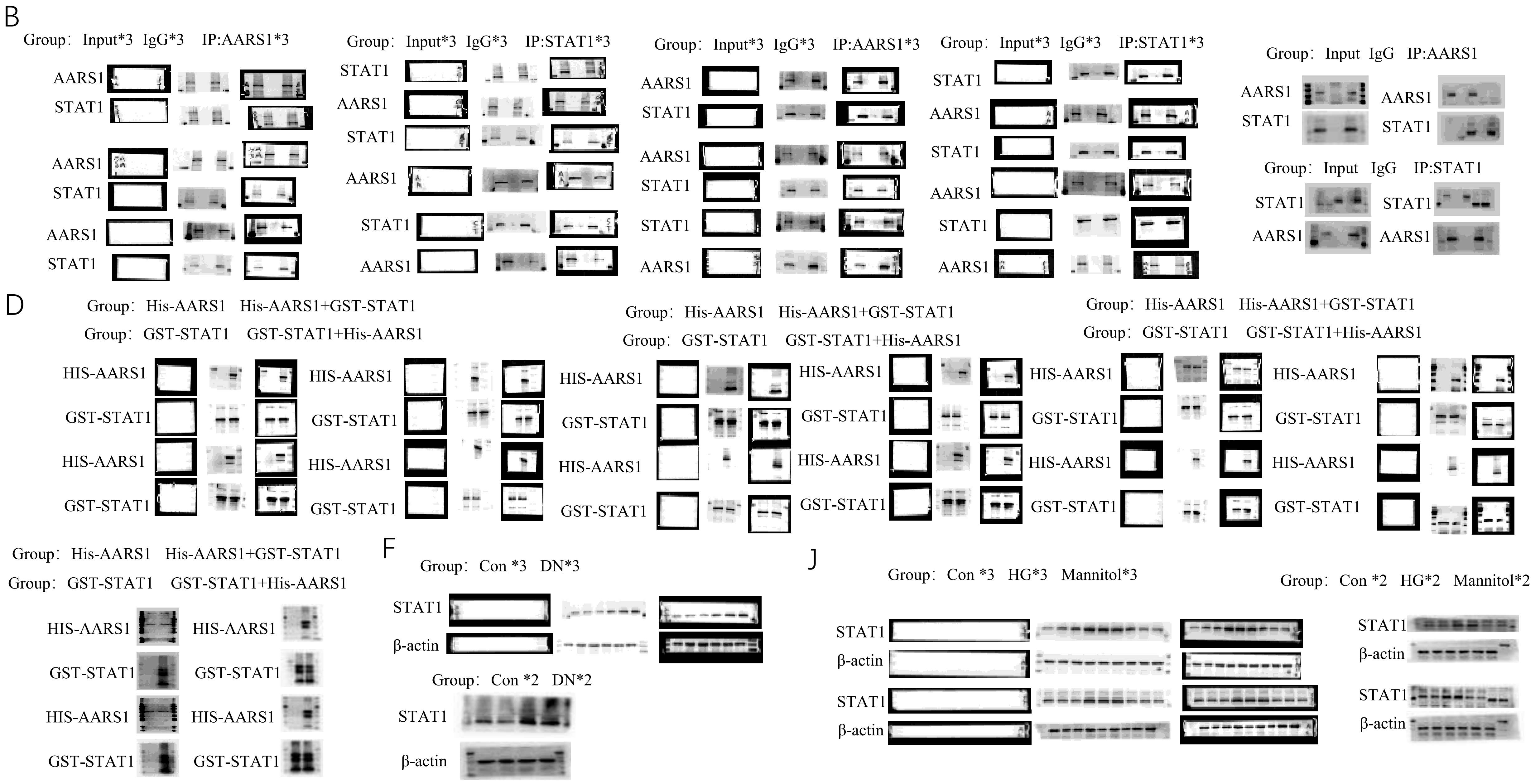

Figure 6 Repeated measurements

B

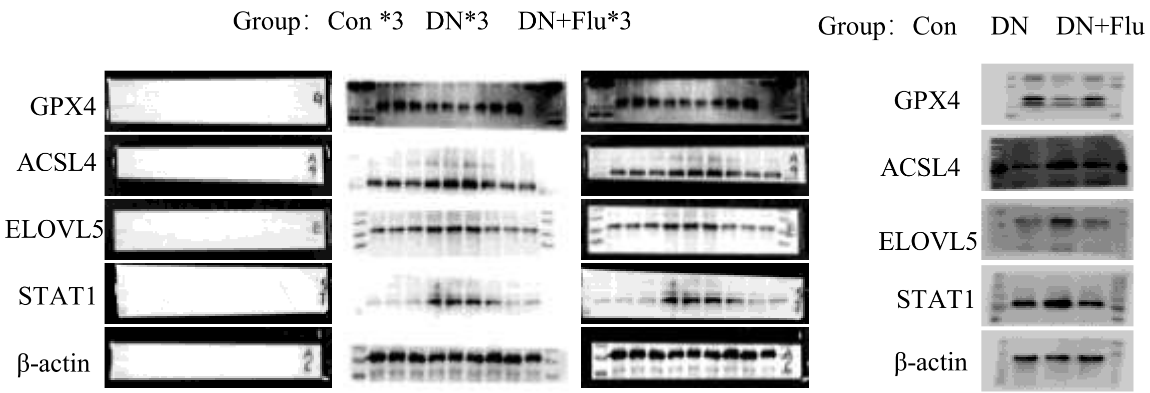

Figure 7 Repeated measurements

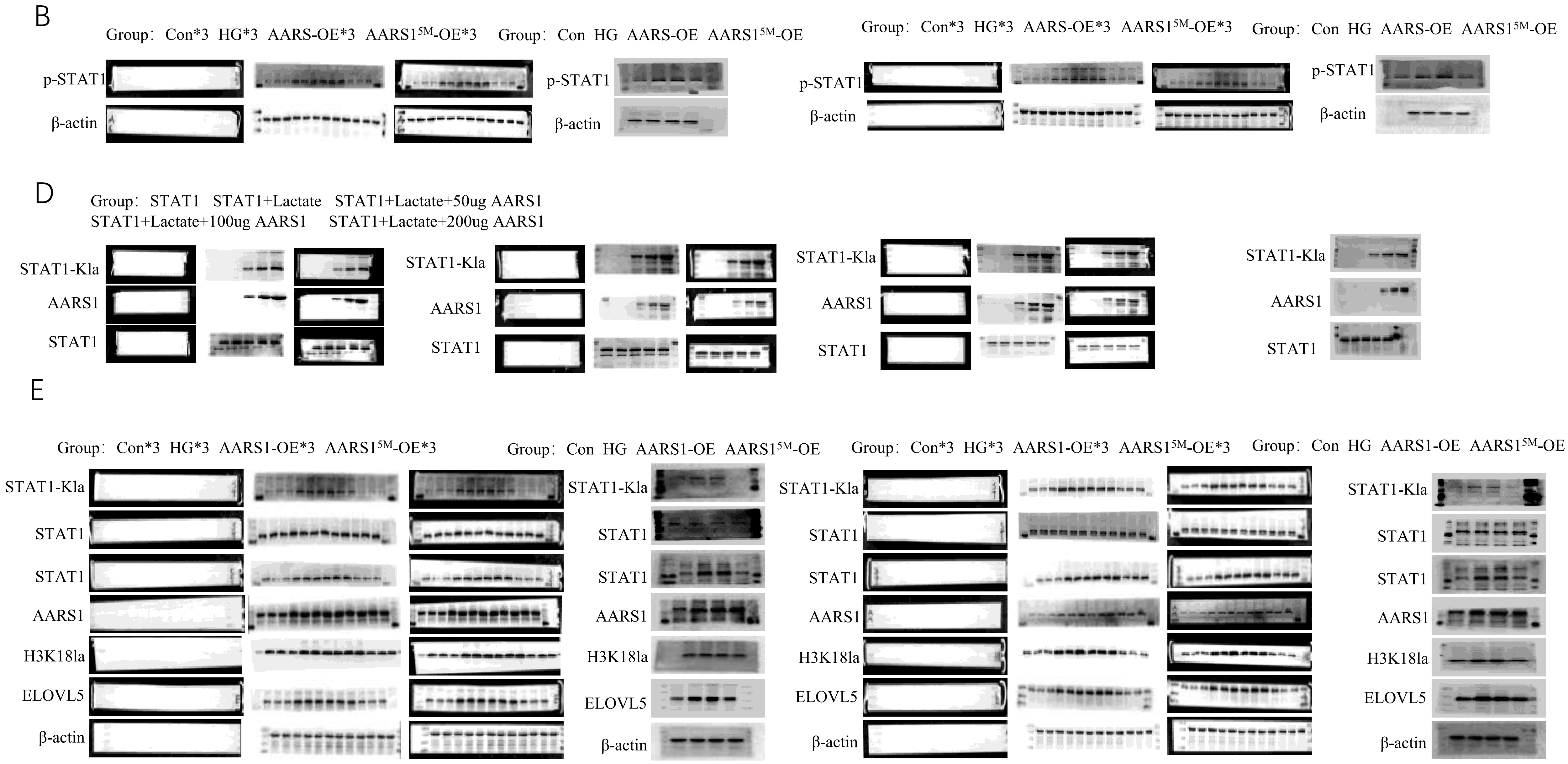

Figure 8 Repeated measurements

B

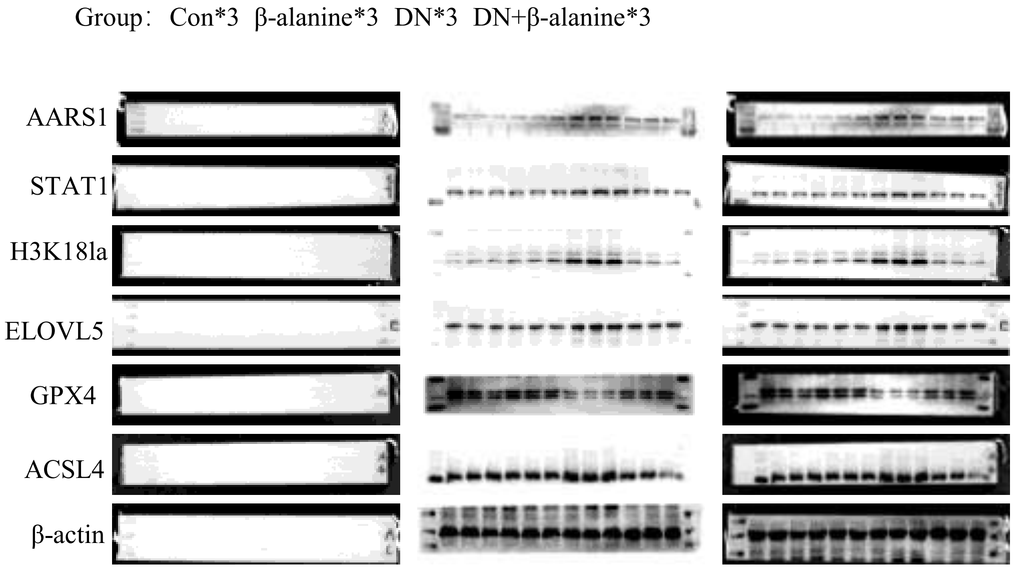

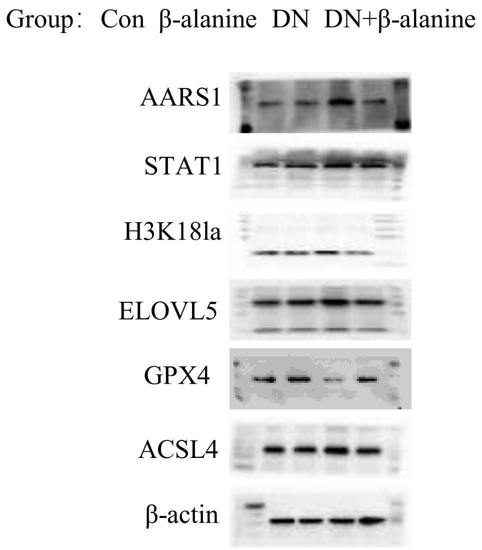

S-Figure 1 Repeated measurements

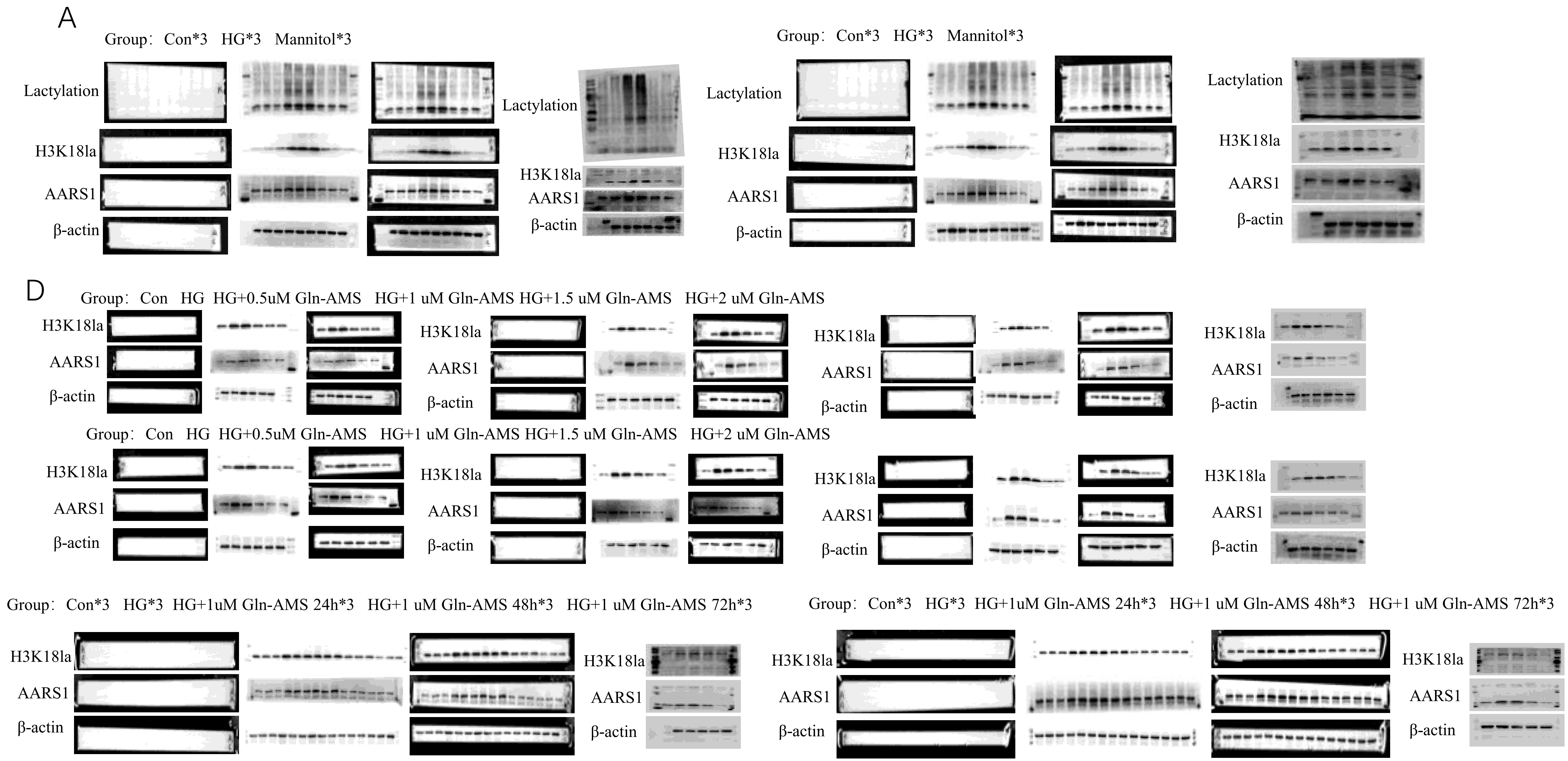

S-Figure 2 Repeated measurements

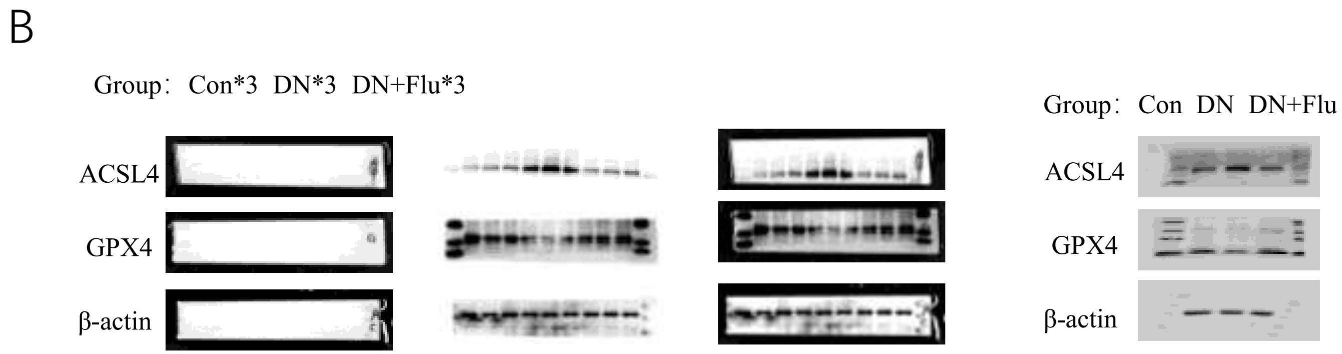

S-Figure 3 Repeated measurements

B

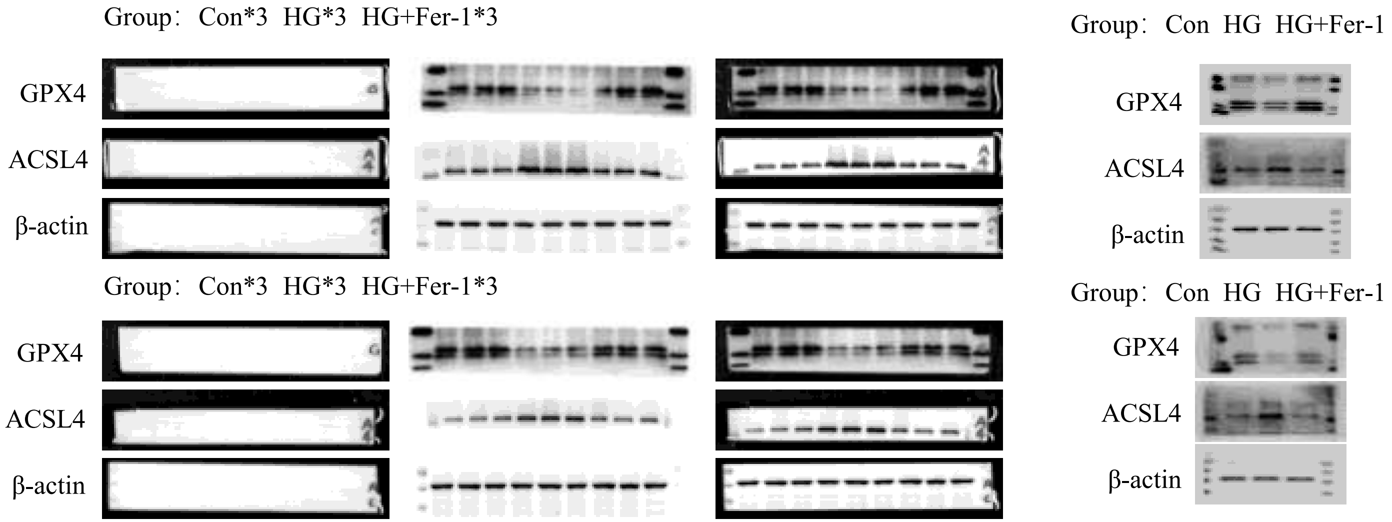

S-Figure 4 Repeated measurements

A

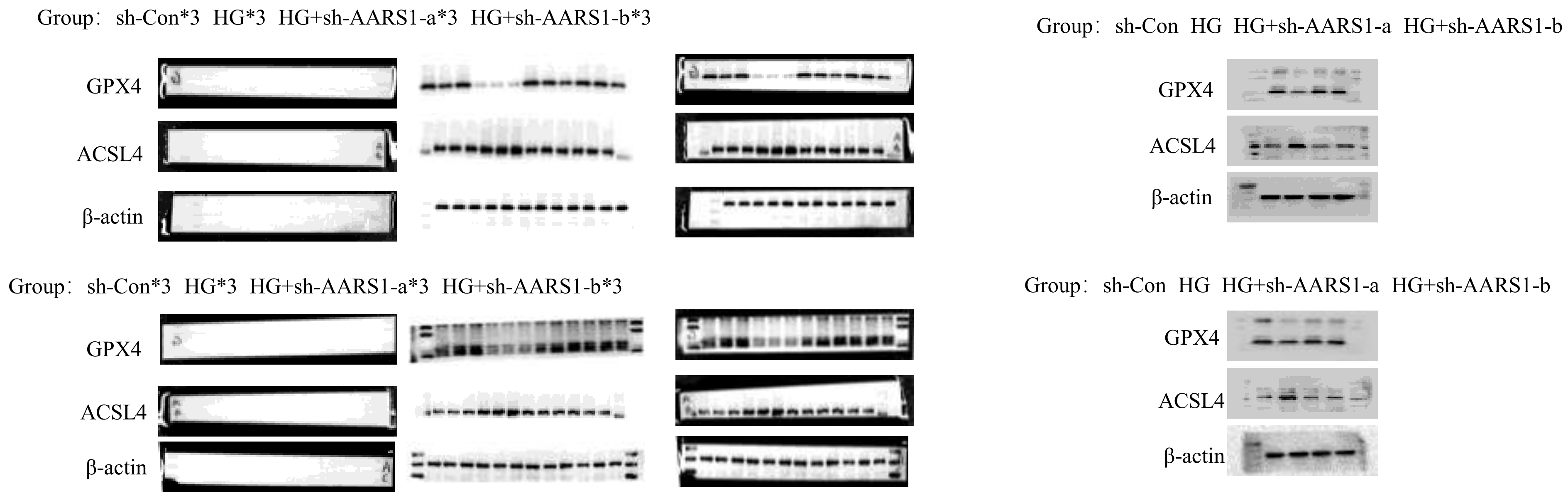

S-Figure 5 Repeated measurements

A

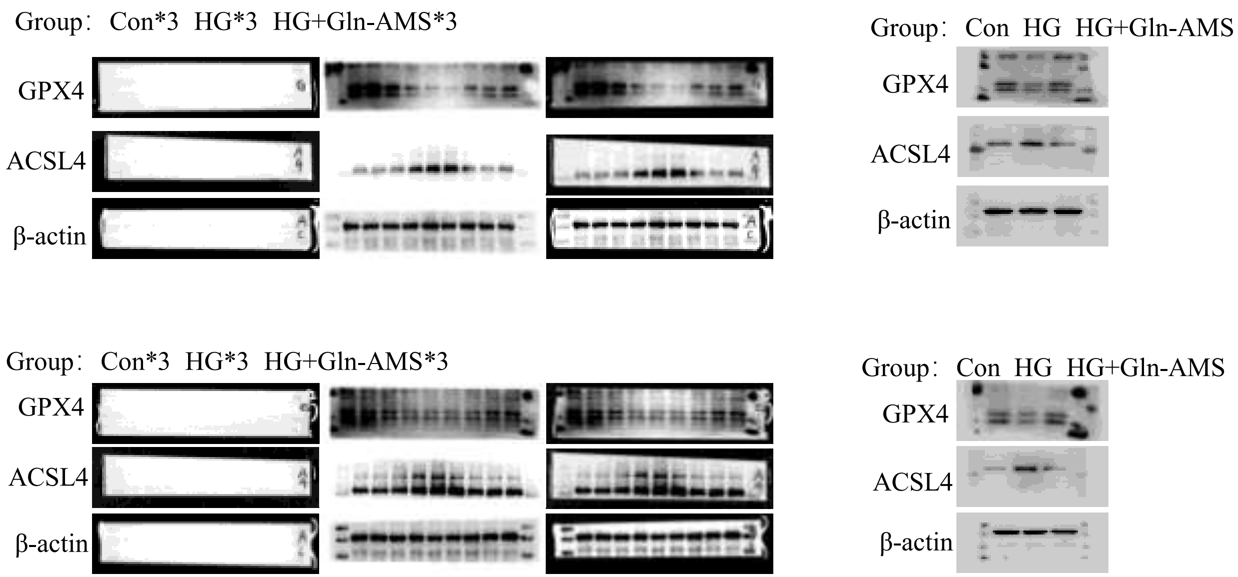

S-Figure 6

Repeated measurements

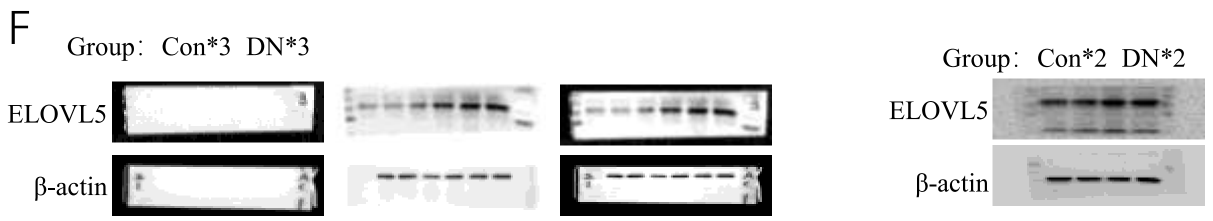

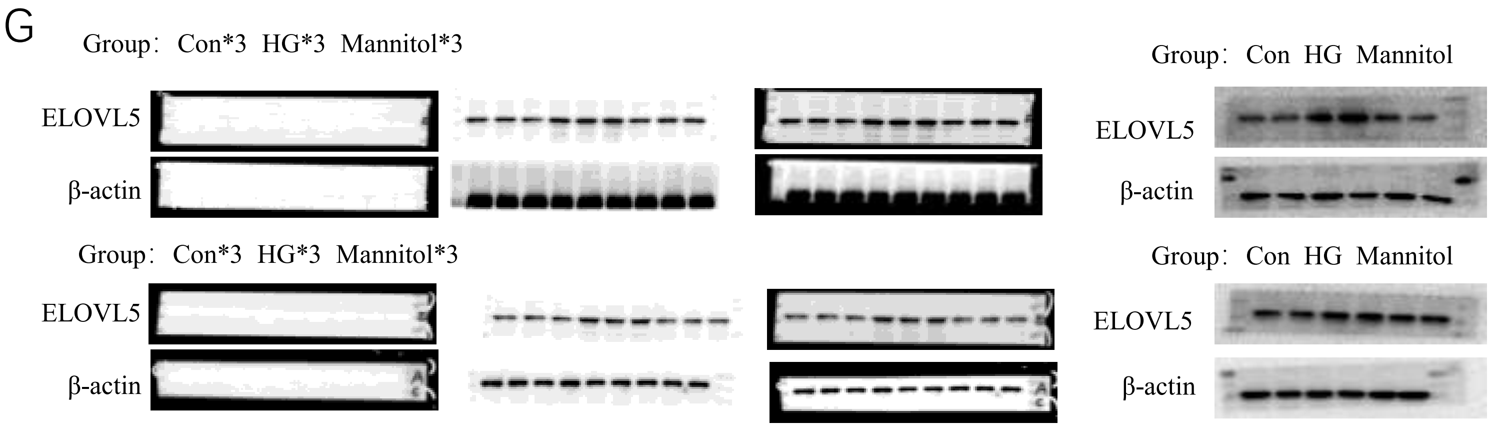

S-Figure 7 Repeated measurements

A

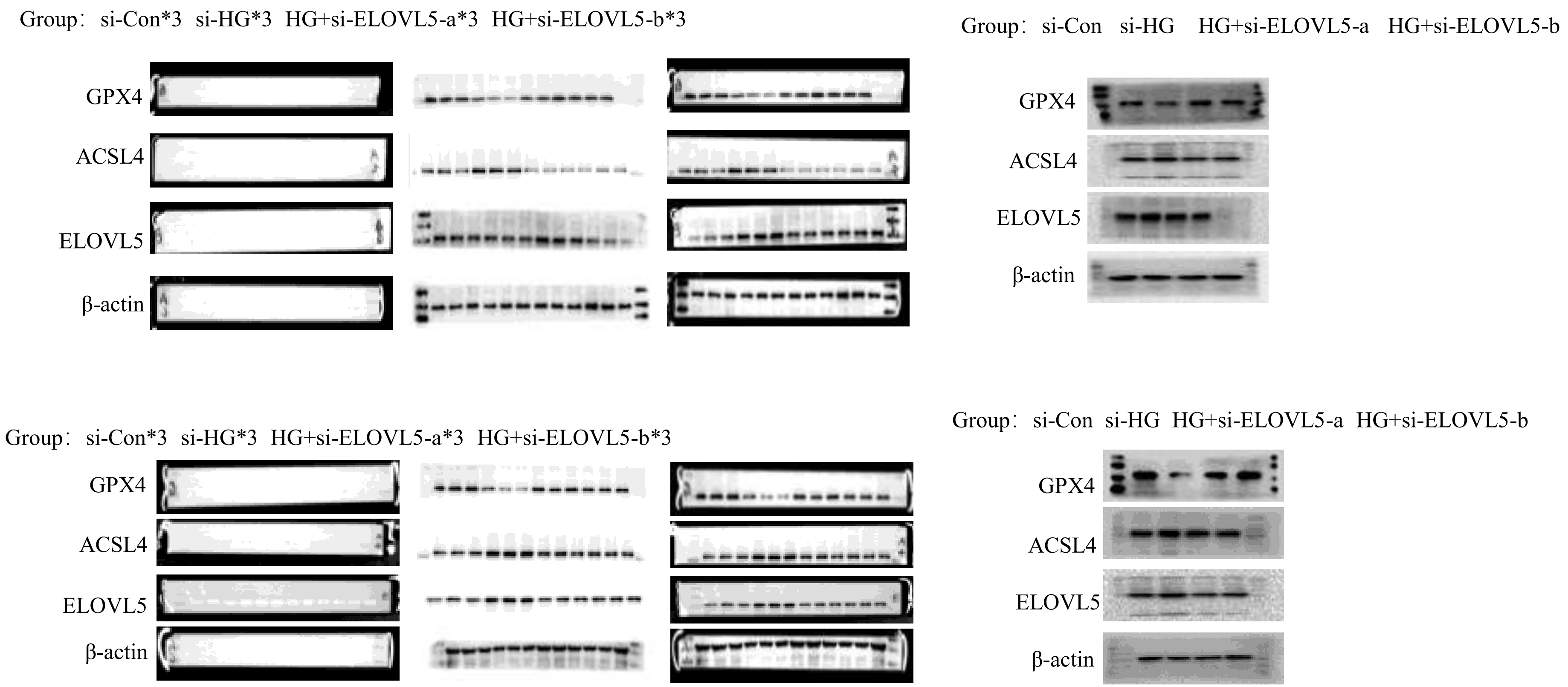

S-Figure 9 Repeated measurements

A

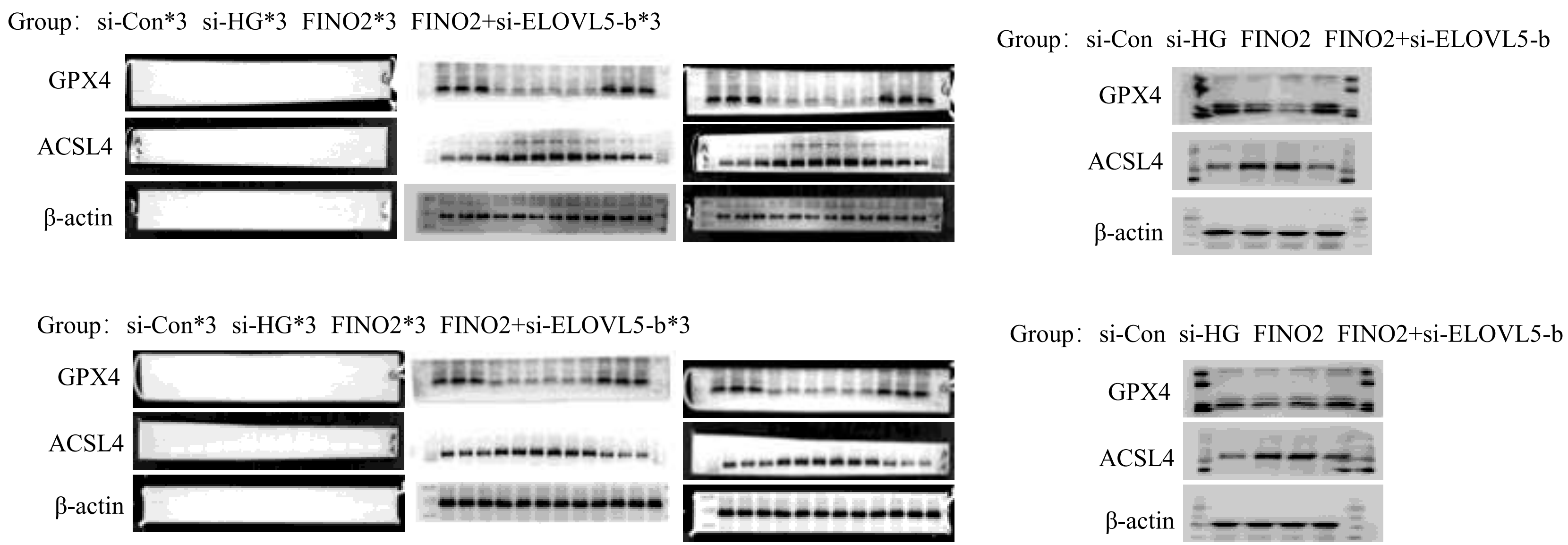

S-Figure 10 Repeated measurements

C

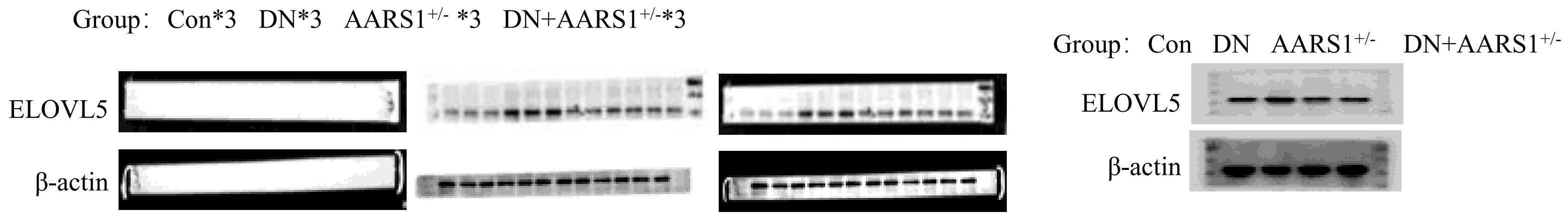

D

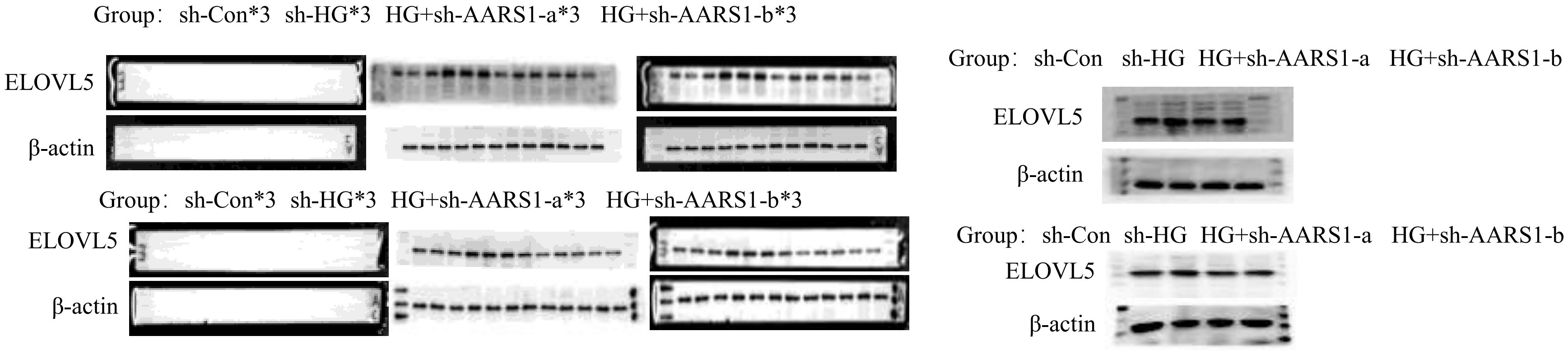

F

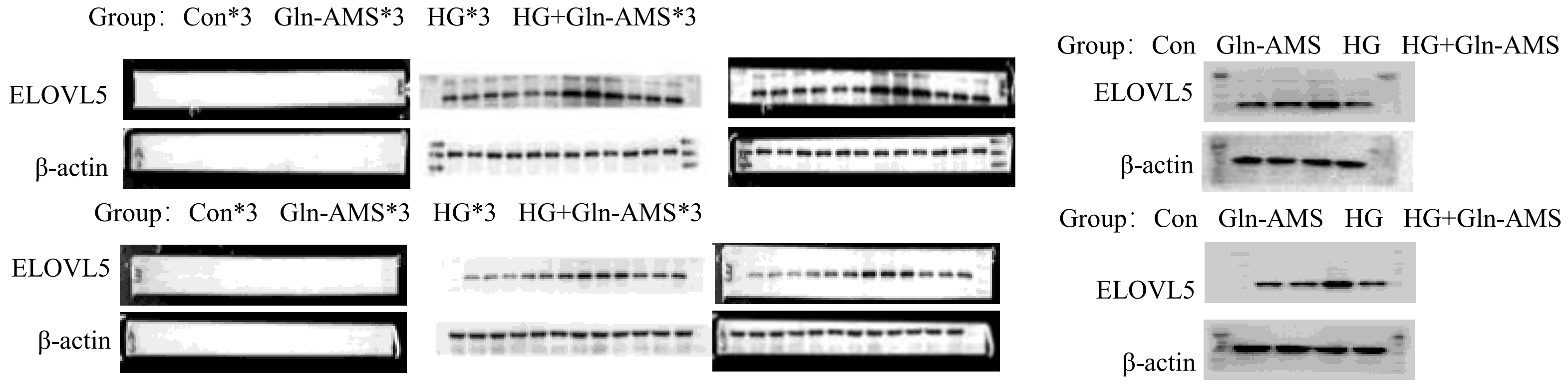

S-Figure 11 Repeated measurements

A

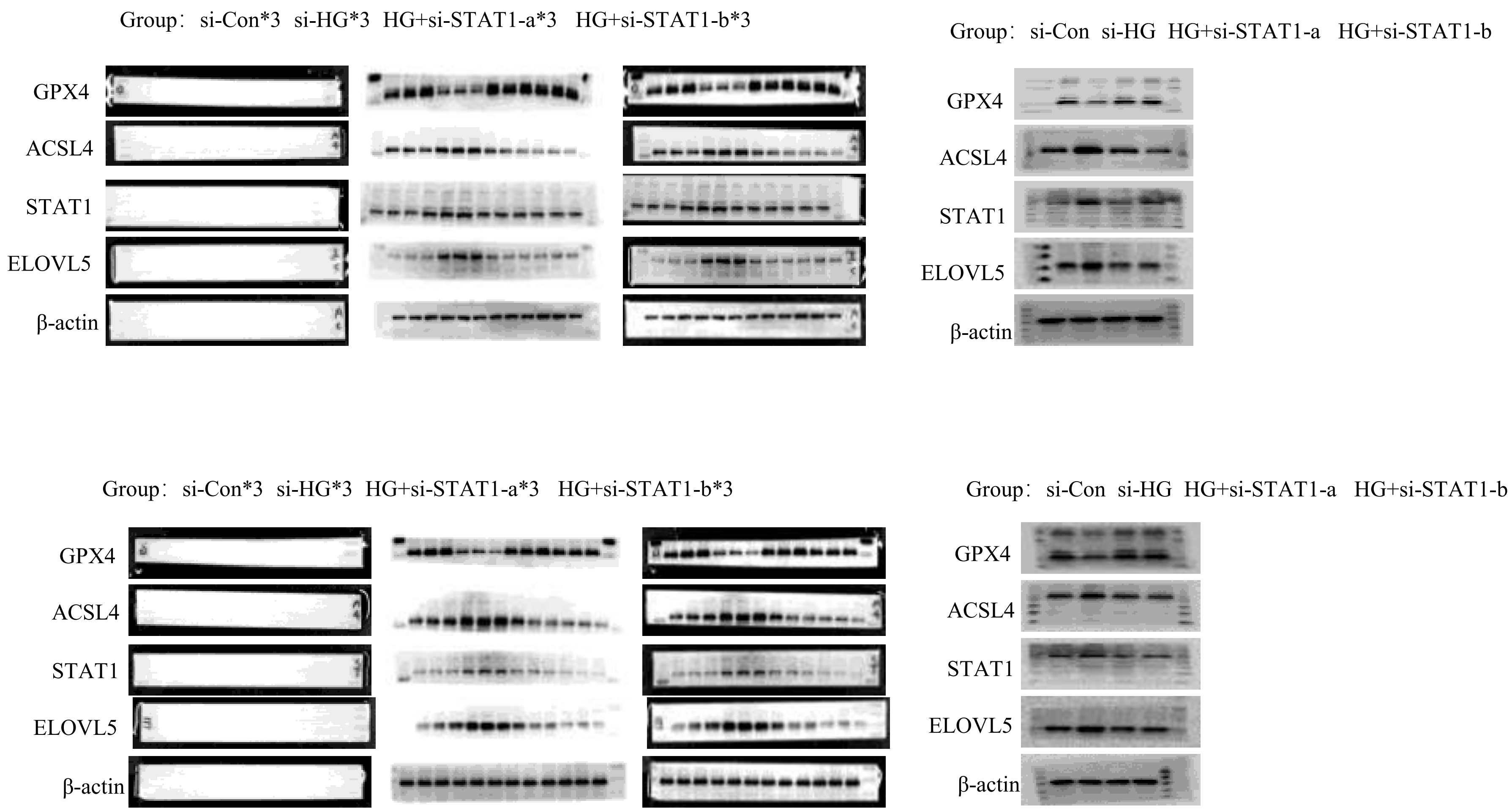

S-Figure 12 Repeated measurements

A

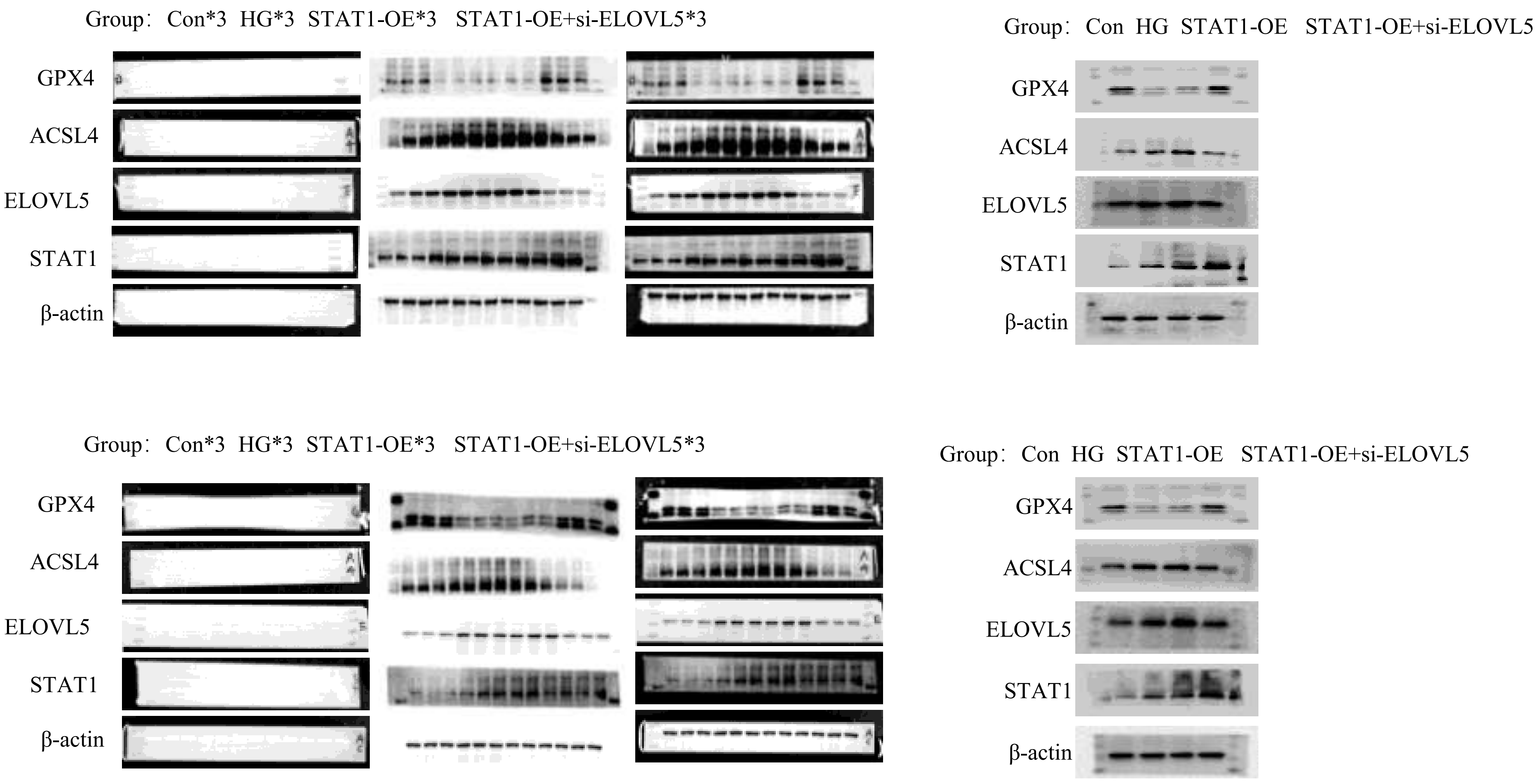

S-Figure 14 Repeated measurements

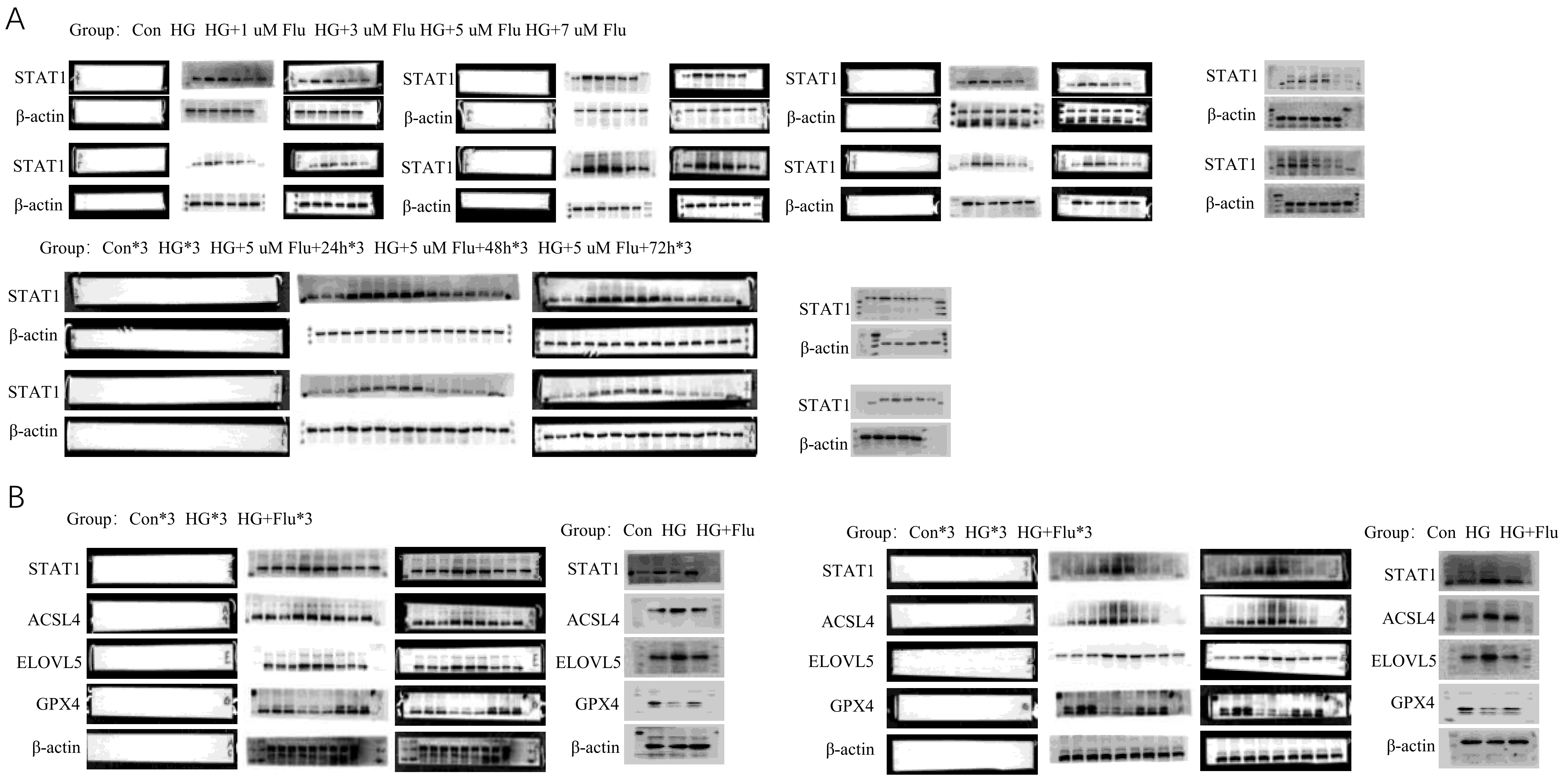

S-Figure 15 Repeated measurements

A

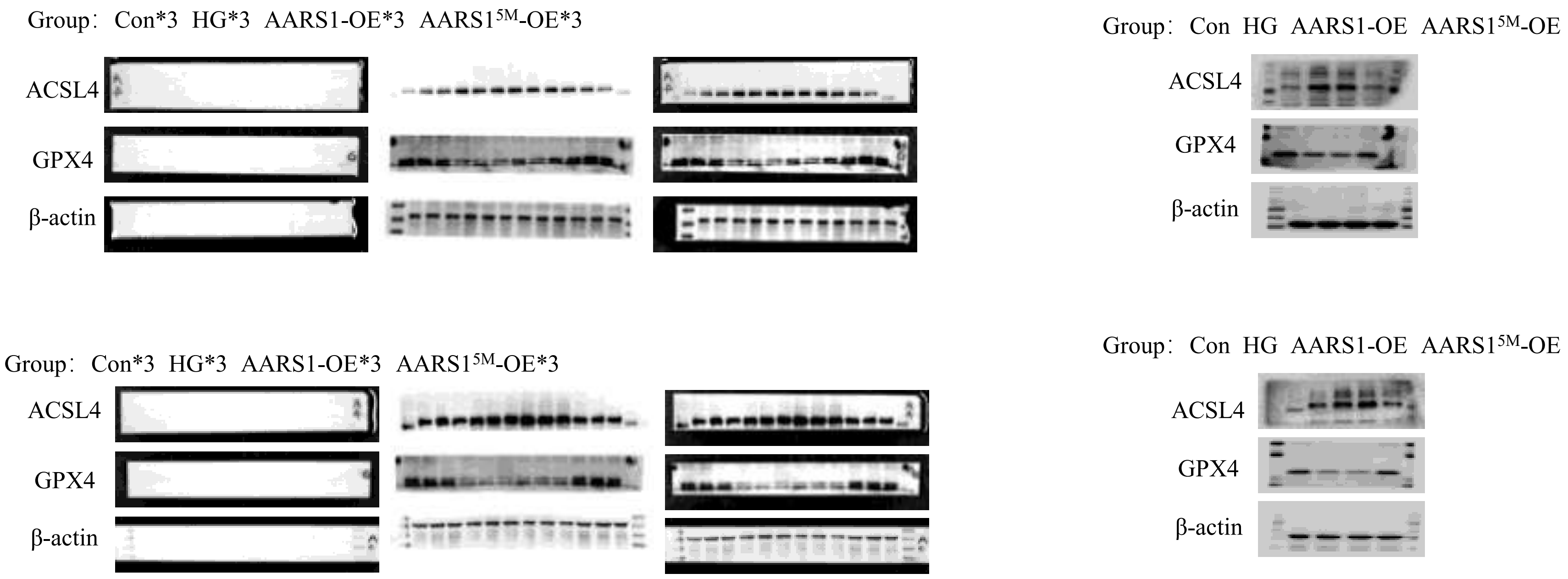

S-Figure 16 Repeated measurements

A

Group：AARS1-OE*3 AARS1-OE+STAT1-WT*3 AARS1-OE+STAT1-K193R*3 AARS1-OE+STAT1-K209R*3 AARS1-OE+STAT1-K685R*3

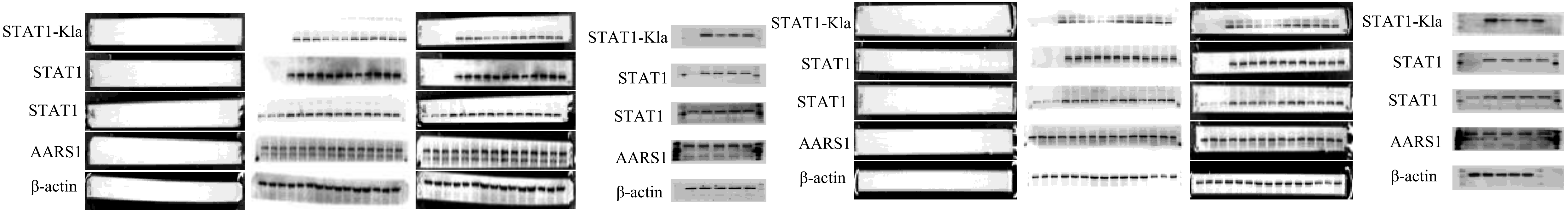

C

Group：STAT1-WT*3 STAT1-WT+AARS1-OE*3 STAT1-K193R+AARS1-OE*3 STAT1-WT+AARS1-OE*3

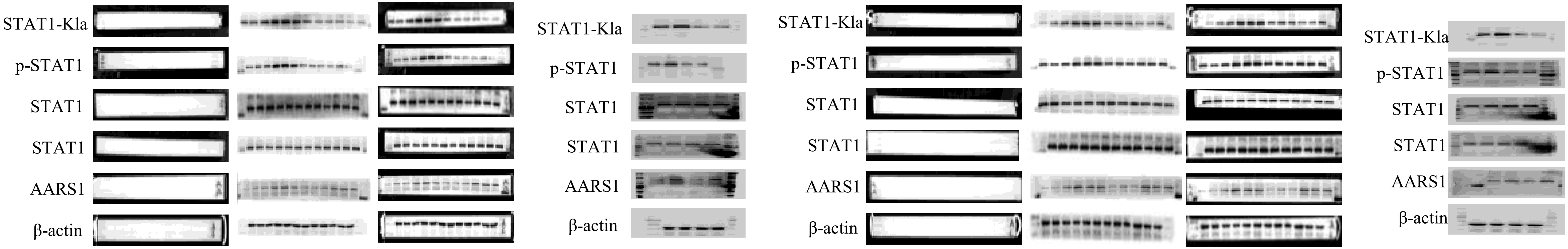

S-Figure 17 Repeated measurements

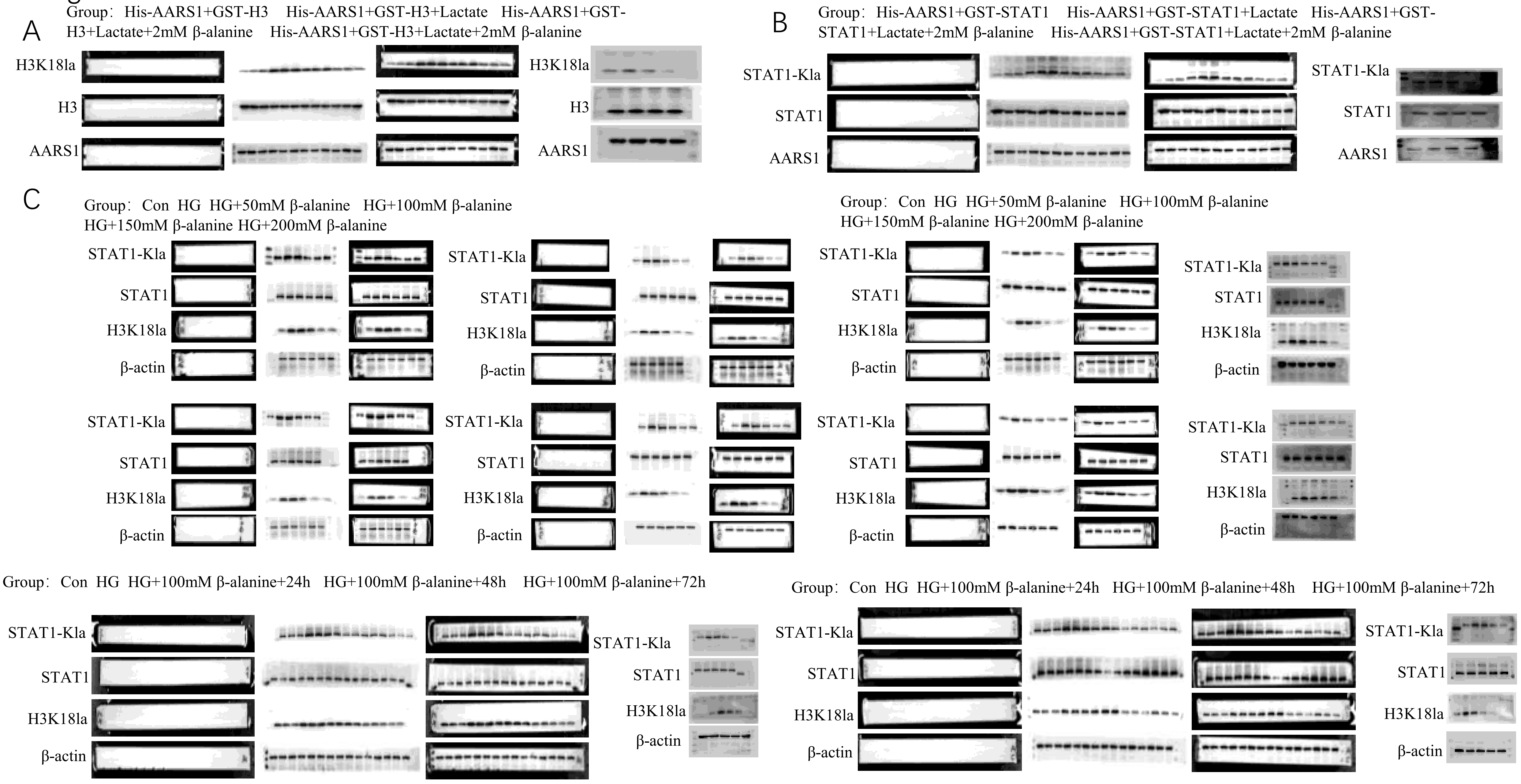

S-Figure 18 Repeated measurements

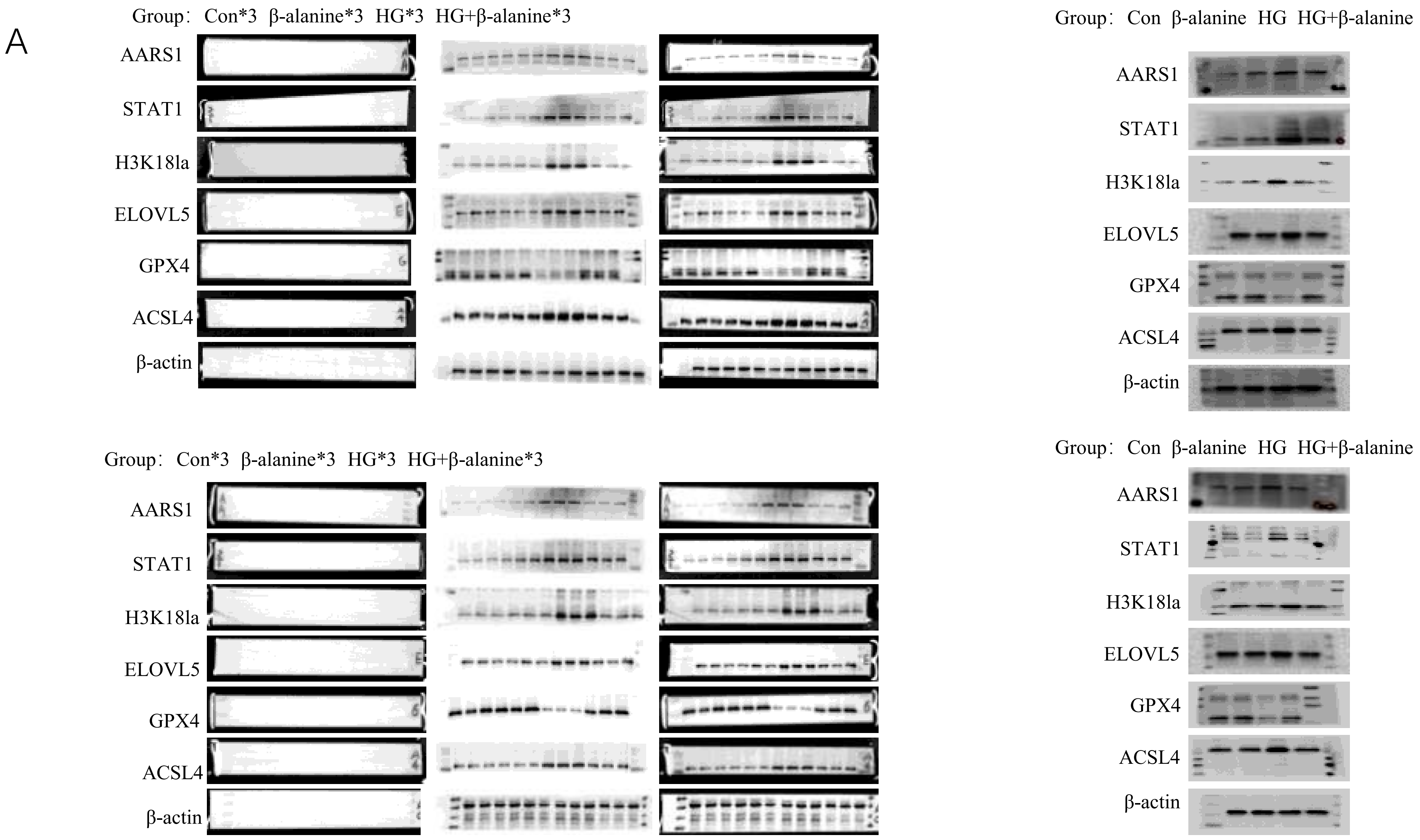

Supplement: Supplementary file 12 — original WB blot [file 41418_2025_1587_MOESM12_ESM.pdf]
