# Supplementary material for: AARS1-mediated lactylation of H3K18 and STAT1 promotes ferroptosis in diabetic nephropathy
Source: Cell Death Differ. 2025 Sep 23;33(3):589–604. doi: 10.1038/s41418-025-01587-4 (PMC13036035; doi:10.1038/s41418-025-01587-4)
Supplement: Supplementary file 13 — gray value of WB data [file 41418_2025_1587_MOESM13_ESM.pdf]

fig 1

kla	Con	DN
	0.73708	1.807549
	1.26292	2.2129
	0.894629	2.181125
	0.989358	2.564512
	1.116013	2.330056

	Con					DN				
AARS1	0.980088	1.165196	0.781082	1.039408	1.206553	2.528545	2.790962	1.954039	1.577107	1.618652
H3K18la	0.996021	0.989109	1.072183	1.004894	0.870393	2.231401	2.034279	2.115154	1.849297	1.950161

fig 2

	Con				DN				AARS1+/-				DN+AARS1+/-			
AARS1	1.310773	0.910602	1.014198	1.086708	1.953179	2.377811	2.349624	1.867237	0.326231	0.303584	0.31272	0.327777	0.394132	0.354186	0.319866	0.317863
H3K18la	1.000052	0.771432	0.992543	1.240037	1.727663	1.759748	1.739951	1.63008	1.089669	1.04529	0.929057	0.989975	1.192149	0.979811	0.821774	1.019992

	sh-Con				sh-HG				HG+sh-AARS1-a				HG+sh-AARS1-a			
AARS1	0.996483	0.998171	0.946211	1.121826	1.638531	2.324962	2.603386	2.774407	0.658652	1.194101	1.157208	1.067848	1.176347	1.183057	1.04087	0.767287
H3K18la	1.002134	0.837883	0.808822	1.156898	1.930415	2.059281	2.087261	2.012512	0.899997	0.999361	1.114194	1.087499	0.815597	1.197065	0.915235	1.10474

	sh-Con				sh-HG				HG+sh-AARS1-a				HG+sh-AARS1-b			
AARS1	0.992508	0.922061	1.120779	0.978691	1.774141	1.904521	1.862301	1.653593	0.651607	1.028694	1.107494	1.110651	0.615513	1.0583	1.168845	0.922061
H3K18la	0.921662	0.940612	1.143329	0.998382	1.672771	2.023226	1.831688	1.692148	1.212482	1.049392	1.129776	1.132997	0.994044	1.079593	1.192362	0.940612

fig 3

	Con				DN				AARS1+/-				DN+AARS1+/-			
ACSL4	1.01465	0.63977	0.688159	1.197283	1.639133	2.108958	2.211487	2.056496	1.168847	1.020319	1.336716	1.309332	0.965723	1.355747	1.358731	0.952138
GPX4	0.997349	1.302501	1.253615	0.851951	0.518045	0.640489	0.362778	0.474028	0.841189	0.701088	1.01011	1.157717	0.935659	1.170821	1.184036	1.202704

fig 4

	si-Con				si-HG				AARS1-OE				AARS1-OE+si-ELOVL5			
AARS1	1.023392	0.901897	1.048815	1.069289	2.031921	2.298343	1.929233	2.141666	1.768862	2.300139	2.024413	2.185863	1.860897	2.101285	2.203527	2.040057
ELOVL5	1.007227	1.079159	0.830946	1.076738	1.791774	1.616737	1.730927	1.567764	1.539342	1.771738	1.660323	1.660525	1.115111	0.726461	0.606304	1.125547
H3K18la	0.99717	1.001779	0.816347	0.968806	1.704544	1.4463	1.543128	1.727211	1.562304	1.790216	1.585639	1.613464	1.51298	1.517328	1.774746	1.519153
ACSL4	1.001389	0.887903	0.877522	1.242514	1.578286	1.769987	1.829619	1.600159	1.836809	1.627887	1.584061	1.828789	1.019215	1.202245	0.991148	1.216741
GPX4	1.014885	1.179687	0.8903	0.940291	0.501301	0.334027	0.334027	0.334027	0.464563	0.42825	0.42825	0.321725	0.833556	0.808467	1.032253	1.252156

	si-Con				si-HG				AARS1-OE				AARS1-OE+si-ELOVL5			
AARS1	1.002682	1.280607	0.612237	1.134952	1.574168	1.717417	2.47729	1.842014	1.630952	2.26823	2.376355	2.010531	1.588125	1.866113	1.713883	1.75815
ELOVL5	1.007852	0.953465	0.863409	0.926212	1.828774	1.624628	1.851157	1.94502	1.562244	1.531971	1.613361	1.667145	0.740236	0.868504	0.648994	0.665066
H3K18la	1.007402	1.032334	0.998412	0.970711	1.686178	1.600151	1.569543	1.830418	1.820893	1.763478	1.716211	1.59055	1.792601	1.591184	1.585701	1.545615
ACSL4	0.9991	0.913241	0.981526	1.082281	1.523368	1.532922	1.906936	1.502908	1.51144	1.430177	1.551922	1.579952	1.065939	0.985639	0.578458	0.566984
GPX4	0.992884	1.020942	1.108455	0.883609	0.595432	0.45972	0.535609	0.379027	0.586618	0.398268	0.395549	0.379324	0.928865	0.948823	0.837225	0.838472

fig 5

STAT1	Con	DN
	0.971724	1.514392
	1.079748	1.86657
	1.163499	1.694846
	0.955931	2.356193
	0.844267	2.60131

STAT1	Con	HG	Mannitol
	1.075051	1.519801	1.074596
	0.906531	1.534442	0.727116
	0.74321	1.846738	1.331738
	1.142585	1.666874	0.909611
	1.131011	1.665199	0.883187

STAT1	Con	HG	Mannitol
	1.097117	1.952803	1.078636
	0.915712	1.77012	0.930641
	0.997181	1.584809	1.137665
	1.135157	1.624405	0.839462
	0.927721	1.666583	0.644675

fig 6

	Con				DN				DN+Flu			
STAT1	1.001114	0.964338	0.991604	1.027094	1.767512	1.949727	2.578884	2.359958	1.208895	1.341013	0.97055	0.930096
ELOVL5	1.007304	0.868812	0.980928	1.145134	1.743943	1.841916	1.961225	1.68026	0.895169	0.823235	0.72263	0.87822
ACSL4	1.003198	0.959375	1.087404	0.990015	1.694431	1.55281	1.952582	1.814676	1.146706	1.036206	0.911583	1.168737
GPX4	0.972223	0.944198	0.981974	1.124778	0.347898	0.488256	0.439725	0.280243	1.024512	0.809593	1.031051	1.342493

fig 7

p-STAT1	Con	HG	AARS1-OE	AARS15M-OE
	1.053714	1.805086	1.861806	1.110676
	0.879173	2.128358	2.430289	1.23994
	1.175922	2.015414	2.168832	1.41564
	0.871699	2.23865	2.454256	1.106741

p-STAT1	Con	HG	AARS1-OE	AARS15M-OE
	0.996623	2.610892	2.415925	1.018653
	0.95812	2.101957	2.017204	0.938311
	1.061554	2.062545	2.562299	1.058432
	0.967471	2.75897	2.496365	1.040652

STAT1-Kla	st	st+Lac	st+Lac+A	st+Lac+A	st+Lac+AARS1 100
	0	0	1.003558	1.776079	2.475997
	0	0	1.02211	1.423359	2.938005
	0	0	1.001276	1.599326	2.324944
	0	0	1.001705	1.370356	1.698424

STAT1-Kla	Con	HG	AARS1-OE	AARS15M-OE
	0.993468	1.577411	1.615862	0.839092
	0.887749	1.58326	1.72369	0.744582
	1.083864	1.564313	1.679707	0.816993
	0.993962	1.672177	1.617603	0.969645

	Con				HG				AARS1-OE				AARS15M-OE			
ELOVL5	0.829674	0.962064	0.886918	1.15944	2.05625	1.739812	1.793293	1.666888	1.689904	1.744863	1.676536	1.567349	1.194723	1.104079	1.000652	1.004962
H3K18la	1.008263	0.996392	0.972701	1.024073	1.82647	1.551182	1.648606	1.527566	1.529029	1.892047	1.562049	1.691434	0.982273	0.916375	0.99845	0.828377
AARS1	1.001313	0.86797	0.98173	1.15178	1.670197	1.505254	1.683551	1.552618	1.504101	1.614878	1.58675	1.499741	1.775152	1.253586	1.194521	1.203203
STAT1	0.996818	1.033916	0.894651	1.061842	1.677363	1.576696	1.800942	1.668278	2.062775	1.848461	1.926076	1.602059	1.239993	0.956528	0.916367	0.882526

STAT1-Kla	Con	HG	AARS1-OE	AARS15M-OE
	0.995904	1.946324	2.074871	1.211918
	0.965418	1.775142	1.723182	0.959768
	0.730593	1.695109	1.702629	0.990565
	1.008213	1.52687	1.454067	1.090562

	Con				HG				AARS1-OE				AARS15M-OE			
ELOVL5	1.008604	0.997834	0.986485	1.02563	1.922694	2.110639	1.816272	1.7358	1.696664	1.820945	1.86163	1.976995	0.982965	1.130608	1.196301	1.066871
H3K18la	0.994395	1.088154	0.989669	0.960095	2.00371	2.347946	1.892575	2.05725	1.910336	1.704685	1.633923	2.291982	1.0608	1.196251	0.966019	1.066443
AARS1	1.004289	1.106036	0.839363	1.077636	1.581175	2.310719	1.996334	2.095054	2.120563	1.942506	1.992396	2.129427	1.653357	1.837966	2.142434	2.5266
STAT1	0.979735	1.004695	1.024949	0.964964	1.77849	2.224748	1.802619	2.184705	1.917991	1.737073	1.849613	2.209228	1.196415	1.266338	1.318781	1.220403

fig 8

	Con				β-alanine				DN				DN+β-alanine			
AARS1	0.979266	1.051621	0.958163	1.008957	1.000112	1.07629	1.021733	1.013116	1.79176	2.181854	1.820023	1.668055	0.979021	1.362132	1.234992	1.208176
STAT1	0.928025	1.013134	0.903058	1.166742	1.10287	1.129379	1.117205	1.153113	1.966413	1.696126	2.300767	1.679061	1.053971	1.28896	1.143271	1.054982
H3K18la	0.995116	1.037237	0.931618	1.219741	0.966925	1.087907	1.03446	1.232204	1.550394	1.775833	1.431513	1.532285	1.192708	0.90001	1.263468	0.835679
ELOVL5	1.004828	0.972597	0.904695	1.160038	0.900221	1.045679	1.131968	0.999082	2.58593	1.501569	1.706576	1.638001	1.075843	1.25993	1.064332	1.262256
ACSL4	0.995497	0.862378	0.889332	1.26617	0.980034	1.08351	1.100976	0.970812	1.569238	1.596923	1.610628	1.5981	1.022363	1.065104	0.958326	0.524792
GPX4	1.294342	1.088566	0.859779	1.031214	0.97786	1.276591	1.259664	1.201993	0.46076	0.364057	0.332712	0.425913	1.150769	0.894382	0.903766	1.192708

s-fig 1

	Con					HG					Mannitol				
lactylation	0.982401	1.023646	1.170385	0.964332	0.857365	1.5187	1.536492	1.769805	2.092832	2.04066	1.037889	0.867669	0.796759	1.068215	0.987033
H3K18la	0.861817	1.008626	0.830722	0.859479	1.282748	1.719134	2.043073	1.94436	1.898318	1.794891	0.871419	0.924062	1.090142	1.182306	0.607436
AARS1	1.006842	0.93082	1.072173	0.882295	1.057019	1.885703	2.199092	1.581673	1.732264	1.604649	0.966064	0.961475	1.097771	0.91703	0.636018

	Con					HG					Mannitol				
lactylation	1.372026	1.27347	1.123109	0.871899	1.011793	1.849402	1.6197	1.958803	2.203499	2.080446	0.858835	0.708913	1.063292	1.194973	0.968848
H3K18la	0.87126	1.19545	1.017113	0.835497	0.883779	1.635864	2.032549	1.45771	1.60043	1.506966	0.972339	0.946259	1.119714	0.818864	0.709134
AARS1	1.00648	0.96559	0.983514	1.112619	0.910797	1.827072	1.970492	1.58796	1.801737	1.506661	1.104191	0.945403	0.940116	0.833037	0.650101

	Con				HG				HG+0.5 Gln-AMS				HG+1 Gln-AMS				HG+1.5 Gln-AMS				HG+2 Gln-AMS			
AARS1	0.996324	1.16927	1.005032	1.056876	1.632815	1.698539	1.498835	1.610063	1.616696	1.651216	1.469184	1.579032	1.204026	1.329718	1.137349	1.223698	1.18149	0.962865	1.237426	1.12726	0.65166	1.00111	0.971029	0.8746
H3K18la	0.987073	1.027699	1.002665	1.034991	1.699412	1.808214	1.484317	1.633171	1.667667	1.718464	1.322228	1.574927	1.359836	1.391263	0.991784	1.233954	0.91352	1.219681	0.926742	1.081283	1.052282	1.050433	0.930114	0.933033

	Con				HG				HG+24h				HG+48h				HG+72h			
AARS1	1.007038	1.040971	1.110624	0.849935	2.075208	1.542582	1.655975	1.626154	2.03933	1.492577	1.518523	1.553108	1.057661	0.959198	0.812823	0.903782	0.891292	0.984337	0.930225	0.806986
H3K18la	1.002244	1.097326	0.970155	0.942517	1.538327	1.503869	1.593945	1.607812	1.4484	1.322925	1.429254	1.556574	0.635606	1.151073	0.969571	0.952317	0.652663	0.903625	0.942183	0.877405

	Con				HG				HG+0.5 Gln-AMS				HG+1 Gln-AMS				HG+1.5 Gln-AMS				HG+2 Gln-AMS			
AARS1	1.002991	1.002539	1.067754	1.024428	1.872217	1.521072	2.24529	1.879526	1.776813	1.497591	2.020526	1.764977	1.49285	1.060806	1.710467	1.421375	0.864806	1.194573	1.47137	1.176916	0.945469	0.741541	1.024466	0.903825
H3K18la	1.00135	1.016521	0.999036	1.005636	2.024128	2.092006	1.590625	1.902253	2.009843	1.847025	1.542443	1.79977	1.37433	1.67689	1.153104	1.401441	1.204893	1.271403	0.67016	1.048819	0.921967	0.72717	0.623013	0.757384

	Con				HG				HG+24h				HG+48h				HG+72h			
AARS1	0.919217	1.157433	0.94437	0.889076	1.597087	1.689821	1.675417	1.651966	1.475085	1.526545	1.592245	1.424358	1.102137	0.966829	0.994324	0.996999	0.656533	1.103782	1.036084	0.997114
H3K18la	0.904946	0.98454	0.946748	1.07726	1.656883	1.768555	1.569095	1.546103	1.524542	1.484988	1.43347	1.428263	0.903337	0.95839	1.023066	0.908659	0.865483	0.866357	0.970857	0.929657

s-fig 2

	Con				DN				DN+Fer-1			
ACSL4	1.006069	0.670464	1.115931	0.845064	1.442477	1.913845	1.741002	2.103893	1.060468	0.982716	1.091079	1.2924
GPX4	1.115286	0.924989	1.254273	1.162914	0.420597	0.36407	0.450011	0.509919	0.81038	0.781155	0.949581	0.879428

s-fig 3

	Con				HG				HG+Fer-1			
ACSL4	0.969679	1.068106	0.886847	1.055862	1.527457	1.947231	1.866585	1.915199	1.220114	1.137046	1.126489	1.272852

GPX4	1.004871	0.980901	1.00186	1.014803	0.384012	0.441011	0.317477	0.354197	1.230157	0.874317	1.198559	1.18113

	Con				HG				HG+Fer-1			
ACSL4	1.020999	0.759329	0.938226	1.276831	1.503411	1.945602	1.781304	2.165734	1.183406	1.109284	1.167621	1.308205
GPX4	1.018133	1.002192	1.029943	0.988695	0.385372	0.433888	0.412746	0.342154	1.213009	1.125043	1.275804	1.151584

s-fig 4

	sh-Con				sh-HG				HG+sh-AARS1-a				HG+sh-AARS1-b			
ACSL4	1.000236	0.999546	1.049319	1.073156	1.528517	1.668415	1.509612	1.647107	0.762707	0.994769	1.081707	0.959055	0.729475	0.907113	1.192212	0.981934
GPX4	1.187626	1.072148	1.140479	0.998918	0.397311	0.335706	0.467471	0.419847	1.023539	1.101925	0.914947	0.923708	1.115055	0.892927	1.136872	1.046219

	sh-Con				sh-HG				HG+sh-AARS1-a				HG+sh-AARS1-b			
ACSL4	1.000049	0.875167	0.980603	1.139893	1.502022	1.570954	1.858058	1.938628	1.050579	1.091396	1.190944	1.241725	0.881582	1.253996	1.140417	1.274783
GPX4	0.998232	0.918928	1.085082	0.995849	0.369302	0.41943	0.335836	0.446702	1.177369	0.805794	1.07616	0.975015	1.166822	1.21536	1.03021	1.078614

s-fig 5

	Con				HG				HG+Gln-AMS			
ACSL4	0.907831	0.76584	1.044174	1.00576	1.551947	1.944108	2.07451	2.124302	1.246764	1.2936	0.958642	1.233996
GPX4	1.125117	1.245493	1.188012	1.04567	0.415758	0.364534	0.434753	0.338737	1.012403	0.812365	1.00551	0.882433

	Con				HG				HG+Gln-AMS			
ACSL4	0.998343	0.863835	0.882321	1.031117	1.688098	1.516695	1.52595	1.51345	1.053606	1.160314	1.207461	1.12329
GPX4	0.961049	1.010431	1.173449	0.999803	0.536069	0.577168	0.534255	0.480506	0.90337	0.829311	0.793105	0.873931

s-fig 6

ELOVL5	Con	DN
	0.925955	2.279318
	1.094515	2.191935
	1.022171	1.952884
	0.989404	1.860969
	1.259868	1.784994

ELOVL5	Con	HG	Mannitol
	0.952622	1.833795	1.030822
	0.821376	1.854442	0.834003
	1.006588	1.738401	1.244268
	1.080562	1.559805	1.084228
	0.902559	1.659941	0.99279

ELOVL5	Con	HG	Mannitol
	0.961799	1.752398	0.946498
	1.040474	1.660141	0.707645
	0.944506	1.569107	0.911439
	1.02258	1.775115	0.980629
	1.220588	1.83136	0.921232

s-fig 7

	si-Con				si-HG				HG+si-ELOVL5-a				HG+si-ELOVL5-b			
ELOVL5	0.997931	0.970091	0.965373	1.082581	1.740141	1.842209	1.9818	2.029547	0.85056	1.092313	1.192249	1.203647	0.985422	1.160764	1.012686	1.192552
ACSL4	0.988771	1.006149	1.138929	1.138938	1.721678	1.92389	2.018593	1.906953	0.839899	0.832784	1.053584	0.978272	0.863019	0.956043	0.824892	0.865002
GPX4	0.976202	1.199246	1.130243	0.951121	0.40926	0.479937	0.399308	0.378377	0.927348	0.946092	1.176842	0.998411	0.970678	1.125085	1.135053	1.046222

	si-Con				si-HG				HG+si-ELOVL5-a				HG+si-ELOVL5-b			
ELOVL5	1.000248	0.767746	1.214378	1.032767	1.62853	1.577804	1.630002	1.699162	0.909884	1.028113	1.244486	1.298654	1.104186	1.083072	1.007833	0.998234
ACSL4	0.986605	0.950688	1.04208	1.049779	1.54421	1.875627	1.958686	1.985303	0.908322	1.187081	1.325315	1.210175	0.84386	1.12419	0.893812	0.783292
GPX4	0.855951	1.070005	1.069682	0.982069	0.249382	0.353244	0.480301	0.402644	1.38131	1.177475	0.958111	1.226274	1.228232	0.910214	1.101409	1.171658

s-fig 9

	si-Con				si-HG				FINO2				FINO2+si-ELOVL5			
ACSL4	0.998211	1.005428	0.927605	1.120633	1.518624	1.778144	1.540399	1.97623	1.67403	1.75089	1.503451	1.810257	1.081992	1.23766	1.173374	1.088916
GPX4	1.021228	1.063754	0.996087	0.936562	0.39903	0.308786	0.442423	0.452781	0.453465	0.590203	0.491115	0.551103	1.09127	1.225458	1.098422	1.175366

	si-Con				si-HG				FINO2				FINO2+si-ELOVL5			
ACSL4	0.998531	0.993052	0.986298	1.038806	1.503743	1.403666	1.588524	1.731235	1.656632	1.71514	1.535253	1.720752	0.981742	0.868559	0.638963	0.579006
GPX4	1.000783	0.921185	1.09873	0.963756	0.380622	0.421529	0.311978	0.458964	0.508923	0.316091	0.370032	0.418647	0.927459	1.01297	0.909274	1.036915

s-fig 10

ELOVL5	Con	DN	AARS1+/-	DN+AARS1+/-
	0.878898	1.812632	0.957918	1.01309
	1.020941	1.8829	0.949338	1.091489
	1.083452	1.737213	0.956202	1.178068
	1.019733	2.097671	1.108878	1.099781

ELOVL5	sh-Con	sh-HG	HG+sh-A	HG+sh-AARS1-b
	1.002716	1.789237	1.066684	1.059954
	0.981198	1.917071	0.889064	1.183336
	0.806115	1.705438	1.198451	1.137815
	1.215985	1.611697	1.088729	0.885958

ELOVL5	sh-Con	sh-HG	HG+sh-A	HG+sh-AARS1-b
	0.989086	1.550632	1.064334	0.981146
	1.090705	1.722871	1.05357	1.006716
	1.078584	1.615374	0.774391	0.759495
	1.056402	1.581391	0.999877	0.856839

ELOVL5	Con	Gln-AMS	HG	HG+Gln-AMS
	1.003646	1.126894	1.570979	1.163903
	1.109662	0.924066	1.874317	0.882682
	1.069068	0.761734	1.767284	1.134043
	0.820775	0.918556	1.640719	0.874149

ELOVL5	Con	Gln-AMS	HG	HG+Gln-AMS
	0.994096	0.886622	1.618955	0.737538
	1.026877	0.983633	1.591972	0.70184
	0.984906	0.961569	1.757471	0.506002
	0.984358	0.959411	1.570598	0.625284

s-fig 11

	Con				HG				HG+si-STAT1-a				HG+si-STAT1-b			
STAT1	1.000743	0.802155	1.186864	0.988768	1.503891	1.827237	2.09048	2.011528	0.751397	1.213314	1.023373	1.059298	0.732745	0.994835	1.240214	1.012302
ELOVL5	1.070915	1.082795	1.069623	0.85024	1.53033	1.829114	1.734879	1.59412	1.273193	1.172441	1.098965	1.100849	1.119032	0.862618	0.908392	1.057338
ACSL4	1.0361	1.201836	1.044718	0.778398	1.647815	1.615881	1.950026	2.021784	1.167125	1.158095	1.066543	1.087735	0.982258	0.822135	0.996569	1.145247
GPX4	1.055477	1.029174	1.008073	0.97258	0.47873	0.439536	0.428182	0.395177	1.000617	1.067426	1.199781	1.092706	1.082247	1.146276	1.009506	1.087799

	Con				HG				HG+si-STAT1-a				HG+si-STAT1-b			
STAT1	1.008537	0.871664	0.963229	1.121992	1.541927	1.901697	1.917216	1.744582	1.021938	1.065109	1.173528	0.954721	1.113247	0.902409	0.82266	0.866149
ELOVL5	0.998035	0.773788	0.943664	1.30907	1.695097	1.675798	1.718131	1.504708	1.190589	0.672376	0.848065	0.700944	0.94706	1.022408	0.634087	0.612563
ACSL4	0.999346	0.97474	1.019737	1.02369	1.675195	1.529497	1.64835	1.665548	0.979041	1.052385	0.780601	0.752842	1.114519	0.768507	0.765125	0.688378
GPX4	1.199497	0.985992	1.135106	0.918611	0.463307	0.418144	0.362411	0.46041	1.019037	1.137036	1.121012	1.208524	0.902388	0.809681	0.888254	1.075943

s-fig 12

	si-Con				si-HG				STAT1-OE				STAT1-OE+si-ELOVL5			
STAT1	0.955629	0.991263	1.049719	0.978442	1.787194	1.72325	1.761708	1.860504	1.953502	1.776348	1.642867	1.703876	1.940578	1.715664	1.84	1.850742
ELOVL5	1.000099	0.973813	0.865413	1.182094	1.605583	1.686931	1.642934	1.797077	1.569033	1.917485	1.871337	1.979943	1.111939	0.99392	0.959612	1.200012
ACSL4	1.082439	0.94489	1.08947	1.039475	1.583946	1.508402	1.536558	1.623205	1.515656	1.602922	1.568682	1.651946	1.148045	1.071091	0.934553	0.88222
GPX4	0.994295	1.016561	1.072206	0.913639	0.474836	0.354343	0.320088	0.387583	0.505026	0.516291	0.5068	0.5216	1.140482	1.114587	1.130598	0.809702

	si-Con				si-HG				STAT1-OE				STAT1-OE+si-ELOVL5			
STAT1	0.955629	0.991263	1.049719	0.978442	1.787194	1.72325	1.761708	1.860504	1.953502	1.776348	1.642867	1.703876	1.940578	1.715664	1.84	1.850742
ELOVL5	1.000099	0.973813	0.865413	1.182094	1.605583	1.686931	1.642934	1.797077	1.569033	1.917485	1.871337	1.979943	1.111939	0.99392	0.959612	1.200012
ACSL4	1.082439	0.94489	1.08947	1.039475	1.583946	1.508402	1.536558	1.623205	1.515656	1.602922	1.568682	1.651946	1.148045	1.071091	0.934553	0.88222
GPX4	0.994295	1.016561	1.072206	0.913639	0.474836	0.354343	0.320088	0.387583	0.505026	0.516291	0.5068	0.5216	1.140482	1.114587	1.130598	0.809702

s-fig 14

	Con				HG				HG+1 Flu				HG+3 Flu				HG+5 Flu				HG+7 Flu			
HGECs	1.002066	0.948824	0.996362	0.998747	1.627534	1.747469	1.807296	1.873679	1.529205	1.606236	1.700089	1.832718	1.278444	1.521928	1.520089	1.592814	0.836478	1.208081	1.273194	1.064344	0.725635	0.818542	1.065947	0.924295
HK-2	1.009045	1.003466	1.029427	1.033409	2.115521	1.725902	1.983048	1.841309	1.989603	1.613189	1.697416	1.819352	1.674856	1.188551	1.63683	1.701824	1.553296	0.851746	1.214155	1.209291	1.037963	0.892204	0.864176	1.059025

	Con				HG				HG+24 h				HG+48 h				HG+72 h			
HGECs	1.001685	1.190912	1.028165	0.901766	1.96552	1.730099	1.99018	2.045395	1.769777	1.542797	1.644734	1.55669	1.569777	1.542797	1.644734	1.55669	0.636046	1.237973	1.147557	1.091619
HK-2	0.941928	0.894038	1.167449	0.985082	2.016289	1.977904	2.040773	1.83427	1.712912	1.429521	1.628771	1.672408	1.312912	1.429521	1.628771	1.572408	0.892945	0.693654	0.74316	0.936055

	Con				HG				HG+Flu			
STAT1	1.012453	1.084628	1.097143	0.814814	1.56941	1.545941	1.662914	1.656945	1.000616	0.985994	1.052647	0.880748
ELOVL5	0.98926	1.15806	0.986782	0.840355	1.73867	1.520715	1.736032	1.678845	1.17402	0.972414	1.028833	0.897533
ACSL4	1.002997	1.114925	1.162309	0.874798	1.774914	1.589395	1.738676	1.89436	1.148584	1.291138	1.234564	1.18903
GPX4	1.012053	1.102301	1.089301	0.893764	0.530125	0.408182	0.541067	0.568307	0.90094	1.328	1.406778	1.035928

	Con				HG				HG+Flu			
STAT1	0.996108	0.998426	1.003477	0.99527	1.753176	1.538175	1.621305	1.685596	0.993176	0.865923	0.921555	0.92326
ELOVL5	0.846798	0.995418	1.091494	1.001803	1.625813	1.727449	1.728825	1.622254	1.103791	1.061642	1.000825	0.809347
ACSL4	1.001952	0.816298	0.881734	1.063173	1.51266	1.581841	1.605089	1.600742	0.771129	1.172754	1.16445	1.049838
GPX4	1.18316	1.150893	1.017292	0.946215	0.454293	0.384331	0.547468	0.424217	0.854866	0.861016	0.74916	1.018293

s-fig 15

	Con				HG				AARS1-OE				AARS15M-OE			
ACSL4	0.982929	0.779968	1.00321	1.113996	1.510145	1.67669	1.641116	1.837873	1.51312	1.730822	1.578214	1.603919	0.826897	1.229699	1.238745	0.933556
GPX4	0.998862	1.128164	1.145006	1.034969	0.635725	0.419768	0.445331	0.469856	0.524365	0.642674	0.538247	0.614975	0.976563	1.096701	1.141722	1.014108

	Con				HG				AARS1-OE				AARS15M-OE			
ACSL4	1.00465	1.044777	1.213728	0.96265	1.50568	1.475291	1.597859	1.661367	1.495124	1.892096	1.506435	1.645569	1.274257	0.951991	1.066497	0.90235
GPX4	1.004837	1.057688	0.942778	0.948276	0.547479	0.416222	0.506523	0.518132	0.531652	0.586322	0.480015	0.557169	0.917976	1.048639	0.903883	1.05411

s-fig 16

AARS1-OE	AARS1-OE	AARS1-OE	AARS1-OE	AARS1-OE+STAT1-K685R
0	1.007836	0.551538	1.076654	1.171809
0	1.170802	0.499831	1.177558	0.763953
0	0.897443	0.575234	1.154213	0.840933
0	0.91009	0.584684	0.819199	0.855486

AARS1-OE	AARS1-OE	AARS1-OE	AARS1-OE	AARS1-OE+STAT1-K685R
0	1.000315	0.598205	1.03511	1.105953
0	1.1704	0.491455	1.012643	1.082335
0	0.81436	0.574741	1.072041	1.338696
0	0.998269	0.590606	1.311345	1.063405

	STAT1-WT				STAT1-WT+AARS1-OE				STAT1-K193R				STAT1-K193R+AARS1-OE			
p-STAT1	1.055898	0.827805	0.997848	1.103164	1.53405	1.966018	1.987372	1.911989	0.927975	0.732487	0.89928	0.914943	0.915906	0.754667	0.844809	0.719094
STAT1-Kla	0.909283	0.896645	1.182812	1.008163	1.752032	1.911353	1.680877	1.669002	0.939772	0.795068	0.970451	0.831443	0.724061	0.800256	0.731012	0.904955

	STAT1-WT				STAT1-WT+AARS1-OE				STAT1-K193R				STAT1-K193R+AARS1-OE			
p-STAT1	1.016412	0.950279	0.816016	1.253406	1.843358	1.617565	1.549843	1.615979	0.812868	1.138715	1.210826	1.194494	1.22388	1.079597	1.096128	1.163259
STAT1-Kla	1.000214	0.924495	0.930174	1.206375	1.618586	1.894418	1.904378	1.841005	0.8318	1.131076	1.087777	1.295247	0.766914	1.16653	1.124609	1.272102

s-fig 17

H3K18la	0	Lac	Lac+2 ala	Lac+2 ala
	0.971095	1.675512	1.219927	0.769708
	1.01937	2.003013	1.432001	1.019607
	0.76534	2.142391	1.464808	1.178529
	1.220033	1.965536	1.687116	0.945191

STAT1-Kla	0	Lac	Lac+2 ala	Lac+2 ala
	0.987476	1.690035	1.218411	0.844658
	1.189937	1.656639	1.408924	1.061559
	0.714673	1.971965	1.450341	0.927539
	0.909089	2.048337	1.541683	1.120862

	Con				HG				HG+50 ala				HG+100 ala				HG+150 ala				HG+200 ala			
STAT1-Kla	1.000301	0.998945	0.995892	1.003935	2.088609	1.558475	1.534515	2.235092	1.893334	1.443696	1.429617	2.423575	1.220996	1.050124	0.928815	1.420837	0.867233	0.947055	0.571812	1.137746	0.938837	1.106733	0.507969	1.173588
H3K18la	1.013641	1.050153	1.026668	0.867599	2.258261	2.373987	2.380802	2.226097	2.264846	2.569325	2.091595	2.308759	1.583962	1.639066	1.764369	1.783133	1.182646	1.163059	1.126752	0.858419	0.985047	1.099726	0.847559	0.677089

	Con				HG				HG+24h				HG+48h				HG+72h			
STAT1-Kla	1.019492	0.888057	1.254574	0.911606	2.243091	2.094269	1.406251	1.636079	1.603778	1.539478	1.164277	1.102779	1.241817	0.942601	0.889525	0.910355	1.483445	0.705919	0.532564	0.449798
H3K18la	1.0058	1.119436	1.03324	0.836028	1.52647	1.840086	1.644801	1.546353	1.343379	1.449753	1.625252	1.409323	0.921785	1.18869	1.279469	0.893381	1.044816	1.00177	0.959207	1.062766

	Con				HG				HG+50 ala				HG+100 ala				HG+150 ala				HG+200 ala			
STAT1-Kla	1.000906	0.931308	1.007606	1.001913	2.579596	2.239348	1.838437	1.570106	2.127149	2.270305	1.607483	1.347313	1.616861	1.302555	0.791553	1.064607	1.331308	0.81433	0.511628	0.821585	1.335656	0.777563	0.543105	0.975838
H3K18la	1.011182	1.003944	0.989044	1.006254	2.339829	1.698668	2.103383	2.391677	2.400354	1.599646	1.866	2.517234	1.649264	0.876901	1.172909	1.698203	0.86761	0.852099	0.623632	1.250178	0.898148	0.79073	0.422591	0.976335

	Con				HG				HG+24h				HG+48h				HG+72h			
STAT1-Kla	0.997649	0.929927	1.010849	1.060295	1.563577	1.622408	1.521829	1.60068	1.134367	1.445393	2.086448	1.584604	0.742663	0.531129	0.792025	0.771276	0.510069	0.598775	0.745027	0.757283
H3K18la	1.003538	1.04614	1.029609	0.924844	1.565979	1.510959	1.831105	1.584213	1.610233	1.386371	1.512415	1.466013	1.024316	0.754033	0.64283	0.499673	0.861379	0.971534	0.788308	0.673449

s-fig 18

			Con				β-alanine				HG				HG+β-alanine	
AARS1	1.001691	1.060696	1.018602	0.973185	0.947525	0.976148	1.115196	1.079463	1.76714	2.111072	1.887879	2.048484	1.165639	0.897325	0.929187	1.051733
STAT1	0.939446	0.900396	0.841833	1.032254	0.949163	1.11124	1.177053	0.85357	2.061837	1.857269	1.834101	1.836064	0.843017	1.127845	1.142885	1.102656
H3K18la	0.999394	0.894137	1.035287	1.035547	1.157638	1.098053	0.869443	1.183906	2.00449	2.162571	1.996655	1.992556	0.992062	1.101119	1.075394	0.825685
ELOVL5	1.054991	0.759922	1.100805	0.87226	1.071994	0.814843	0.956798	0.775853	1.67834	1.84325	1.563558	1.518494	1.065978	1.012339	0.834563	1.14519
ACSL4	1.023258	0.897132	0.940744	0.97797	0.977021	0.916189	1.188853	1.129007	1.584534	1.661355	1.551081	1.659652	0.966732	1.003307	0.989448	0.987553
GPX4	1.011677	0.932505	0.866325	1.080004	1.012129	0.838919	0.992417	0.902836	0.332548	0.425503	0.484523	0.40406	1.027438	1.084762	0.956378	1.015942

			Con				β-alanine				HG				HG+β-alanine	
AARS1	1.001691	1.060696	1.018602	0.973185	0.947525	0.976148	1.115196	1.279463	1.76714	2.111072	1.887879	2.048484	1.165639	0.897325	0.929187	1.051733
STAT1	0.939446	0.900396	0.841833	0.803225	0.949163	1.01124	1.177053	1.185357	2.061837	1.657269	1.534101	1.716064	0.843017	1.218278	1.142885	1.202656
H3K18la	0.999394	0.894137	1.035287	1.035547	1.157638	1.098053	0.869443	1.183906	2.00449	2.162571	1.996655	1.992556	0.992062	1.101119	1.075394	0.825685
ELOVL5	1.054991	0.759922	1.100805	0.87226	1.071994	0.814843	0.956798	0.775853	1.67834	1.84325	1.563558	1.518494	1.065978	1.012339	0.834563	1.14519
ACSL4	1.023258	1.197132	0.940744	0.77797	0.977021	0.916189	0.888853	1.129007	1.584534	1.661355	1.951081	1.859652	0.966732	1.203307	1.189448	1.217553
GPX4	1.011677	0.932505	0.866325	0.900036	1.012129	0.838919	0.902417	0.902836	0.532548	0.425503	0.484523	0.540598	1.027438	0.804762	0.956378	1.015942
